# Advancing Fundamental Understanding of Retention Interactions in Supercritical Fluid Chromatography Using Artificial Neural Networks: Polar Stationary Phases with –OH Moieties

**DOI:** 10.1021/acs.analchem.4c01811

**Published:** 2024-07-29

**Authors:** Kateřina Plachká, Veronika Pilařová, Tat’ána Gazárková, František Švec, Jean-Christophe Garrigues, Lucie Nováková

**Affiliations:** † Department of Analytical Chemistry, Faculty of Pharmacy in Hradec Králové, Charles University, 500 05 Hradec Králové, Czechia; ‡ SOFTMAT (IMRCP) Laboratory, SMODD Team, CNRS, Toulouse III Paul Sabatier University, 31400 Toulouse, France

## Abstract

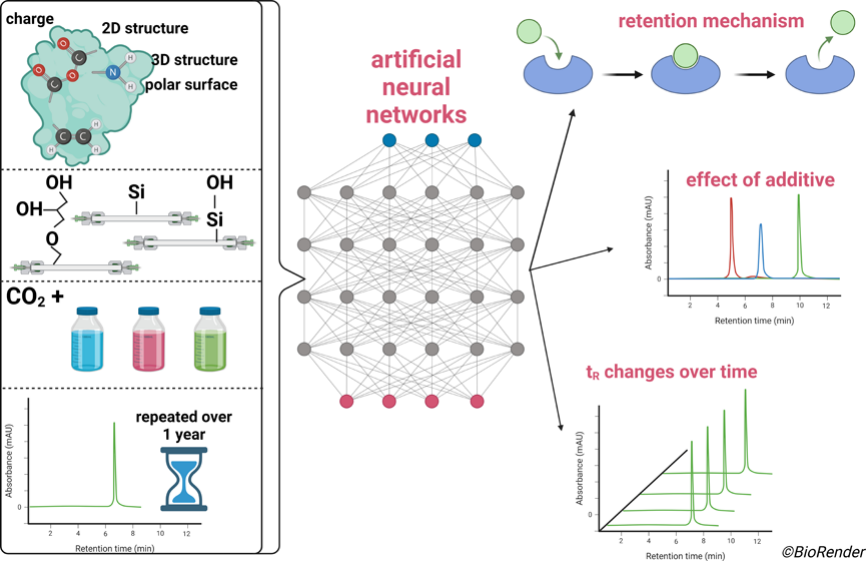

The retention behavior in supercritical fluid chromatography and its stability over time are still unsatisfactorily explained phenomena despite many important contributions in recent years, especially focusing on linear solvation energy relationship modeling. We studied polar stationary phases with predominant –OH functionalities, i.e., silica, hybrid silica, and diol columns, and their retention behavior over time. We correlated molecular descriptors of analytes with their retention using three organic modifiers of the CO_2_-based mobile phase. The differences in retention behavior caused by using additives, namely, 10 mmol/L NH_3_ and 2% H_2_O in methanol, were described in correlation to analyte properties and compared with the CO_2_/methanol mobile phase. The structure of >100 molecules included in this study was optimized by semiempirical AM1 quantum mechanical calculations and subsequently described by 226 molecular descriptors including topological, constitutional, hybrid, electronic, and geometric descriptors. An artificial neural networks simulator with deep learning toolbox was trained on this extensive set of experimental data and subsequently used to determine key molecular descriptors affecting the retention by the highest extent. After comprehensive statistical analysis of the experimental data collected during one year of column use, the retention on different stationary phases was fundamentally described. The changes in the retention behavior during one year of column use were described and their explanation with a proposed interpretation of changes on the stationary phase surface was suggested. The effect of the regeneration procedure on the retention was also evaluated. This fundamental understanding of interactions responsible for retention in SFC can be used for the evidence-based selection of stationary phases suitable for the separation of particular analytes based on their specific physicochemical properties.

Supercritical fluid chromatography (SFC) has undergone an important transformation over the years to increase its applicability in various fields.^1^ As a result, SFC evolved from a marginal method used primarily for the analysis of nonpolar compounds to the method of choice for the analysis of compounds with a wide range of polarity and physicochemical properties.^1^ However, this technique is still primarily considered a research tool rather than a routine method, even though the causes of the technique’s negative reputation, including a lack of method robustness, instrument unreliability, and complex technology transfer, were already mitigated.^2^ In recent years, several studies on interlaboratory validation have been carried out confirming the robustness and repeatability of SFC methods.^3−5^ Nevertheless, several negative aspects related to long-term retention time (*t*_R_) stability have also been described for SFC.^6,7^

Current state-of-the-art SFC typically uses a mobile phase containing CO_2_ and organic modifiers such as methanol (MeOH) or other alcohols. The organic modifier can interact with the free acidic silanols on the silica surface of the stationary phase to form silyl ethers.^6^ Silyl ether formation (SEF) reduces the number of free silanols that are no longer involved in the interactions between the stationary phase surface and analytes, causing changes in selectivity over time. In addition, the SEF reaction forms water as a byproduct, which acts as a polar additive and affects the retention and separation selectivity. SEF can be catalyzed by acid and base, commonly used as additives to the mobile phase. The reaction kinetics can also be correlated with the modifier composition.^6^ The SEF is a condensation reaction that can be reversed by water. Therefore, a small percentage of water in the organic modifier, i.e., 2–5%, could shift the equilibrium toward the free silanols and mitigate the SEF. Thus, the process of regeneration, where the column is washed with a large volume of water, is suggested to reverse the SEF.^6−9^ However, the SEF phenomenon is still not well understood, and more detailed studies are necessary. Furthermore, organic acids, ammonia, water, and/or buffers can be added to the mobile phase, affecting the separation of acidic/basic analytes and their peak shapes. This additive can be adsorbed on the silica surfaces of the stationary phase.^10^ Its removal presents an additional problem, resulting in a change in selectivity over time, especially when different additives are used on the same column.^11−13^

Polar stationary phases with predominant –OH functionalities have been used in more than 35% of published works.^9^ At the same time, the –OH functionalities of these phases are more prone to SEF.^9^ The well-established linear solvation energy relationship (LSER) classification^14^ sorts these columns among the polar stationary phases in two clusters (Supporting Information Figure S1): (i) nonbonded silica and hybrid silica and (ii) polar ligands bonded to the silica surface. Polar ligands also include other functional groups such as amino groups, cyano groups, and 2-ethylpyridine groups which will be discussed within the following paper. Looking at the individual parameters of the LSER equation (Supporting Information, Figure S1B), all columns discussed in this paper have strong dipole–dipole and π–π interactions (terms *e* and *s*), hydrogen bonding with acids and bases (*a* and *b* terms), and interactions with cations (*d*^+^ term). The main differences can be seen in the magnitude of the terms. Furthermore, the differences in retention behavior can be correlated to different physicochemical properties of –OH functionalities. Free silanols in bare silica are easily ionizable and thus affected by the mobile phase pH. Their p*K*_a_ has been estimated to be 4 – 7.^15,16^ p*K*_a_ values vary for different types of silanols, including geminal, isolated, and vicinal. Some silanols can form strong hydrogen bonds with water via proton sharing, indicating a higher acidity (p*K*_a_ ≈ 2.9 – 4.6). In contrast, some silanol groups with p*K*_a_ ≈ 8.9 can be deprotonated, i.e., forming SiO^–^, and be stabilized by nearby –OH.^15,16^ For hybrid stationary phases, different acidities are expected as the free unreacted silanols are sterically and hydrophobically hindered by methyl groups to prevent further attack of the silica surface by the mobile phase. Additionally, a less acidic support is used. In fact, the p*K*_a_ for the first generation of hybrids (XTerra) was estimated to be ≈ 9 – 11 based on the mobile phase composition. For the diol column, the p*K*_a_ of –OH functionalities was estimated to be around 14.^15−18^ Later, two other terms, sphericity (*gG*) and flexibility (*Ff*), were added to the LSER classification.^19^ The positive contribution of *g*, indicating a higher retention of spherical molecules, and the negative contribution of *f*, indicating a lower retention of flexible molecules, were described.^19^ However, no model taking into account also the localization of the charge and detailed parameters of the 2D and 3D analyte structure has been proposed, yet.

Our study focuses on the determination of the differences between bare silica, hybrid silica, and diol columns and increases the knowledge of SEF. Three mobile phase compositions were tested, including CO_2_ with (i) neat MeOH with apparent pH ≈ 5, (ii) MeOH + 2% H_2_O with apparent pH ≈ 1, and (iii) MeOH + 10 mmol/L NH_3_ with apparent pH ≈ 7 – 8.^20^ The tested organic modifiers were selected to cover the most commonly used SFC mobile phases. MeOH enabled us to describe the retention mechanism without interactions caused by the additive. Furthermore, the results obtained using MeOH served as a baseline for the evaluation of SEF. The use of MeOH + H_2_O causes acidic apparent pH of the mobile phase similarly to other acidic additives such as formic acid.^20^ Moreover, the beneficial effect of H_2_O addition on retention stability in SFC has been previously reported.^5^ MeOH + NH_3_ was selected as the most straightforward example of an ammonium-based additive. Indeed, when using ammonium salts as additives, both ions, e.g., ammonia and formate, can affect the retention mechanism. In our study, we can be certain that all of the observed interactions are caused by either MeOH or NH_3_. Furthermore, Ovchinnikov et al. showed that diethylamine and ammonium acetate caused identical changes of LSER parameters.^21^ All experiments within our study were carried out under typical SFC conditions to enable easy transfer of the results.^12,22^ Structures of >100 analytes were described by topological, constitutional, hybrid, electronic, and geometric descriptors. This extensive set of experimental data was used to train the artificial neural networks (ANN) simulator with deep learning toolbox which then linked the structure of the analytes to the observed retention. The aims of the study included the following: (i) a fundamental description of the retention behavior on polar stationary phases related to specific molecular features of the analytes, (ii) quantitative description of the changes in retention behavior during one year of column use and their explanation with a proposed interpretation of changes on the stationary phase surface, and (iii) the investigation of the effect of the regeneration procedure.

## Experimental Section

### Chemicals

Methanol (MeOH), acetonitrile (ACN), 2-propanol (IPA), and water of LC/MS grade quality were provided by VWR International (Prague, Czech Republic). Ammonia (4 mol/L) solution in MeOH for LC/MS was purchased from Sigma-Aldrich (Steinheim, Germany). Pressurized liquid CO_2_ 4.5 grade (99.9995%) was purchased from Messer (Prague, Czech Republic). Most of 107 reference standards listed in Supporting Information Table S1 were purchased from Sigma-Aldrich (Prague, Czech Republic). Several standards were kindly donated by Zentiva, k.s. (Prague, Czech Republic).

### Standard Solutions

Standard solutions of all reference standards were prepared by dissolving each compound in MeOH. The reference standards were then divided into 12 mixtures specific for each column and organic modifier and diluted to the final concentration of 50 μg/mL by ACN.

### Analytical Instrumentation and Procedure

The experiments were carried out using an Acquity UPC^2^ SFC system (Waters, Milford, MA, USA) equipped with a binary pump, an autosampler, a column thermostat, a back pressure regulator (BPR), and a PDA detector. The system was coupled to a single quadrupole detector (QDa, Waters) via a commercial SFC-MS dedicated pre-BPR splitter device with an additional isocratic pump for the make-up solvent delivery (Waters).

A generic gradient method was used with a mobile phase consisting of (A) CO_2_ and (B) organic modifier at a flow rate of 1.5 mL/min and following gradient program: 2% B for 1 min, 2–45% B in 1–5 min, followed by 1 min of isocratic step at 45% B and 1.5 min of equilibration at initial conditions. Three organic modifiers were tested: MeOH, MeOH + 10 mmol/L NH_3_, and MeOH + 2% H_2_O. The column temperature was 40 °C and the BPR pressure was 13 MPa. The BPR was adjusted for each measurement sequence to avoid *t*_R_ variations due to changes in the system pressure. The BPR was manually adjusted before each sequence so that the system pressure for the blank injection overlapped the system pressure of the first sequence within 0.07 MPa. The autosampler temperature was 10 °C and the injection volume was 2 μL. Peak detection and integration was carried out using the PDA detector, with data collected in the range of 210 to 400 nm. The MS detector with electrospray ionization in positive and negative modes enabled the confirmation of each analyte. MeOH + 10 mmol/L NH_3_ was used as a make-up solvent at a flow rate of 0.3 mL/min.

### Columns and Regeneration Procedure

Three stationary phases with the same dimensions (100 × 3.0 mm) were tested: nonbonded silica (Zorbax HILIC Plus, Agilent Technologies, Inc., CA, USA, *silica*), bridged ethylene hybrid (Viridis BEH, Waters, *BEH*), and high density diol with pure propanediol linker (Torus Diol, Waters, *diol*). All columns were packed with 1.7 μm particles except for the silica column with 1.8 μm particles. Prior to the first injection, the columns were flushed with CO_2_/MeOH (50/50) at 1.5 mL/min for 35 min to eliminate further retention shifts.^23^ A separate column was used for each organic modifier, but the three columns were always from the same batch to mitigate interbatch variability and ensure the same retention properties. Column regeneration was carried out on an Acquity UPLC system, Waters (Milford, USA). The procedure, in agreement with previous findings and Waters Column Care & Manual Guide,^6,7^ included washing with >200 column volumes of H_2_O at 0.6 mL/min for 280 min, followed by >10 column volumes of IPA/H_2_O (9/1, v/v) at 0.5 mL/min for 20 min, and >10 column volumes of IPA at 0.5 mL/min for 20 min.

### Study Design

Eight data points were collected for each column at defined time periods: first injection (month 0), month 1 (1M), 2M, 3M, 6M, 9M, and 12M. The column was then regenerated according to the regeneration procedure, and the last data point (R) was collected. Prior to measurement at each data point, the column was flushed with the CO_2_/organic modifier (55/45) at 1.5 mL/min for 15 min and then equilibrated with the CO_2_/organic modifier (98/2) at 1.5 mL/min for 15 min. Blank, standard mixtures, and blank were injected within the sequence, each in triplicate. After the use, the column was washed with CO_2_/MeOH (55/45) at 1.5 mL/min for 30 min (>20 column volumes) and neat CO_2_ at 0.6 mL/min for 30 min. CO_2_ was used as the storage solvent^6^ to avoid column aging and to eliminate *t*_R_ shifts.

### Data Evaluation

Raw data were processed using Empower 3 to collect *t*_R_ and peak widths at 5% of peak height. The % change in *t*_R_ over time was calculated for each analyte, column, and organic modifier (Microsoft Excel, version 2302). The 3D structures of analytes were optimized by semiempirical AM1 quantum mechanical calculations using the MOPAC application of Chem 3D Pro version 14.0 software (CambridgeSoft). A root-mean-square gradient of 0.100 was used to minimize the energy for all of the compounds. These optimized structures were then used for computing 2D and 3D molecular descriptors (CDK Descriptor Calculator, v.1.4.8). The 226 calculated molecular descriptors included topological, constitutional, hybrid, electronic, and geometric descriptors of the 2D and 3D structure of the molecule and are listed in Supporting Information Table S2 and categorized in Supporting Information Table S3. Molecular descriptors and retention factors were normalized by dividing by the maximal value.

To identify key molecular descriptors linking the structure of analytes to their retention on different stationary phases, ANN were created using the neural network simulator in Matlab R2023a with the deep learning toolbox V.23.2 (The MathWorks, Inc., Massachusetts, USA) and a sigmoid activation function, a back-propagation learning algorithm with 500 learning cycles. These ANN were structured with an input layer connected to the 226 molecular descriptors and an output layer linked to the retention factor (*k*′) of each analyte. After 500 training cycles, the weights assigned to each input neuron were extracted, and the key molecular descriptors, with weights greater than 1.5 in absolute value, were examined. The higher the weight assigned by the ANN, the more the descriptor affects retention.^24^ The differences related to the organic modifier used were determined. The standard deviation (SD) of the molecular descriptor weights at each data point were calculated and correlated with the observed changes in retention behavior. The molecular descriptors with the largest changes in weight over time were determined and used to describe changes in the stationary phase surface over time.

To determine the adequacy of the regeneration procedure, the % error between the *t*_R_ at the first injection and after the regeneration were calculated: %-error = (*t*_R_ at the first injection – *t*_R_ after regeneration)/*t*_R_ at the first injection. The effectiveness of the regeneration procedure used was obtained by comparing two % differences between: (1) at the first injection and at 12M versus (2) at the first injection and after regeneration. If the (2) % difference is lower than (1), then it means that the regeneration procedure resulted in a *t*_R_ closer to the first injection than the *t*_R_ observed at 12M.

## Results and Discussion

Our study was conducted in agreement with previous findings.^7,11^ (i) Switching additives within one column should be avoided to maintain repeatable *t*_R_. Therefore, a separate column was used for each organic modifier. This also allowed us to distinguish between the effects of additive and organic modifier on the *t*_R_ shifts. (ii) A longer equilibration of the stationary phase with a higher proportion (> 20%) of organic modifier with the additive is recommended to cover the stationary phase surface and ensure efficient *t*_R_ repeatability. In our study, 45% of organic modifier was used. (iii) It is strongly recommended to rinse the column after use with large volumes of organic solvent and neat CO_2_ (> 30 column volumes) and to store the columns in CO_2_ to prevent SEF.^6^

**Figure 1:**
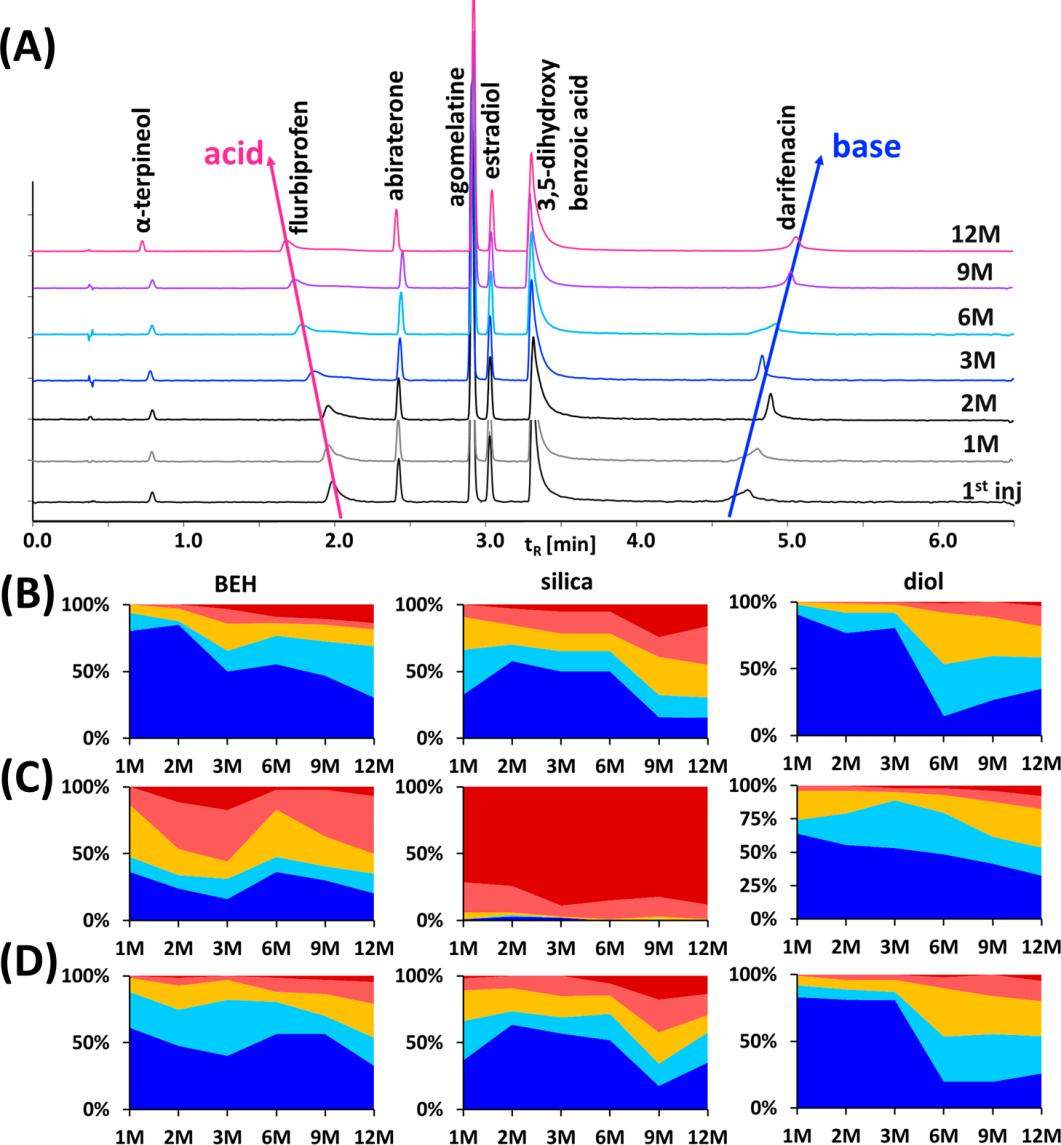
(A) Overlay of chromatograms for selected compounds analyzed on the BEH column using MeOH + 10 mmol/L NH_3_ at different data points and retention time shifts over time on selected stationary phases using (B) methanol, (C) MeOH + 10 mmol/L NH_3_, and (D) MeOH + 2% H_2_O as organic modifier, expressed as %-difference: less than 0.5% (dark blue), 0.5–1.0% (light blue), 1.0–2.0% (yellow), 2.0–5.0% (light red), and over 5.0% (dark red).

Despite following these recommendations, instability of *t*_R_ was observed. First, a simple comparison of % changes in *t*_R_ over time was carried out where the first injection (0M) was considered 100%. Figure 1A shows a comparison of the chromatograms obtained at each data point for one of the standard mixtures measured on the BEH column. When using the same set of compounds for the comparison, the *t*_R_ shift between the injections at 0 and 1M was >1% for more than 33% of the compounds eluting on the silica column when using MeOH as an organic modifier. This variation was smaller in the case of BEH and diol. Here, only 6 and 2% of the compounds had a *t*_R_ shift exceeding 1% after 1 month, respectively (Figure 1B). However, this percentage progressively worsened over time. The *t*_R_ shift was also dependent on the organic modifier used, especially in case of the silica column. Overall, the *t*_R_ instability increased when NH_3_ was added to the organic modifier, contrary to the better *t*_R_ stability observed with MeOH + H_2_O.

The observed differences in *t*_R_ shifts were related to the physicochemical properties of the analytes. The tested standard mixture shown in Figure 1A contains neutral, acidic, and basic compounds. Although the *t*_R_ values were mostly stable for neutral compounds such as estradiol, a decrease in retention was observed for acidic flurbiprofen, in contrast to an increased *t*_R_ of basic darifenacin. Thus, all analytes were described using 226 molecular descriptors to fully understand the correlation between the physicochemical properties of analytes and the retention behavior over time.

### Retention Mechanisms on Polar Stationary Phases with –OH groups

The retention factors calculated from the *t*_R_ obtained at 0M on the new column were used to describe the retention mechanism using ANN for the data evaluation. In total, three different compound sets were used throughout the study. The (i) original set included all 107 analytes measured on all tested stationary phases. However, not all compounds eluted using all tested analytical conditions. Thus, the (ii) narrowed set included the 52 analytes eluting on all three stationary phases and the (iii) extended sets contained all compounds eluting on each stationary phase. The (ii) narrowed set was used for the evaluation of retention using MeOH. Subsequently, three (iii) extended sets, each specific to the stationary phase, were used for detailed description of the retention behavior using each organic modifier.

### Retention Mechanisms Using Methanol as Organic Modifier

In the first step, the narrowed set was used for the evaluation to allow a direct comparison of the retention mechanism between tested stationary phases. The ANN determined the weights by which each molecular descriptor affected the retention. Positive values of the weights correspond to increasing retention, while negative values correspond to decreasing retention. The heatmap showing the overview of the weights obtained for each molecular descriptor is shown in Supporting Information, Figures S2–S4. The top 20 molecular descriptors affecting retention to the largest extent on each stationary phase are listed in Supporting Information, Table S4. These molecular descriptors were selected as the descriptors with the highest values of weights in positive (10 descriptors) and negative (10 descriptors). In most cases, decrease in values of assigned weights was observed after these top 10 + 10 molecular descriptors. These molecular descriptors for each column/organic modifier are discussed within this paper in detail, and the reader is referred to Supporting Information, Figures S2–S4, for the effect of the other descriptors.

Four molecular descriptors that decreased retention were the same for all three stationary phases (Supporting Information, Table S4): LipinskiFailures, BCUTc-1h, BCUTc-1l, and RPCG. LipinskiFailures is a 2D descriptor consisting of a set of five rules originally related to the solubility and pharmacokinetic properties of drugs.^25^ This parameter takes into account failures with respect to the defined limits of five criteria: < 5 hydrogen donors, < 10 hydrogen acceptors, < 500 Da, and log *P* < 5. BCUT is a weighted version of the Burden matrix that considers both the connectivity and the atomic properties of a molecule. The BCUTc parameters describe the highest (−1h) and lowest (−1l) partial charge in the molecule. The BCUTc-1h descriptor gives positive values based on the partial charge of the molecule, i.e., the higher the partial charge, the higher the value of BCUTc-1h. In contrast, BCUTC-1l is calculated in negative values. The RPCG descriptor also confirmed that the retention on these stationary phases is strongly affected by the charge state of the molecule. RPCG calculates a relative positive charge of the molecule, i.e., most positive charge/total positive charge. Higher values of RPCG are observed for compounds where the positive charge is localized in a small part of the molecule, as opposed to multiple functional groups with positive charge throughout the molecule resulting in lower RPCG values. Lower retention of positively charged compounds on silica and diol columns was observed also by Si-Hung et al.^26^

Most of the molecular descriptors strongly decreasing retention on silica and BEH columns were the same as expected. Retention decreased with increasing hydrophobicity expressed as *X*Log*P* corresponding to previous results,^26^ increasing distance edge between all primary oxygens (MDEO-11), and with the presence of S atoms bonded through two double and two single bonds (khs.ddssS). Weta2.unity, as a descriptor decreasing the retention, is a holistic WHIM (weighted holistic invariant molecular) descriptor related to the density of atom distribution, i.e., the amount of unfilled space per projected atom.^27^ In general, WHIM descriptors based on a number of atom weightings are informing about the 3D molecular structure in terms of size, shape, symmetry, and atom distribution.^27^

The RHSA parameter again confirms the reduced retention for compounds not in a charged state. RHSA is calculated as the sum of the solvent-accessible surface areas of atoms with an absolute value of partial charges less than 0.2/total surface area. Thus, a higher value of RHSA is expected for compounds with no or a low partial charge on the surface of the molecule. The combination of RPCG and RHSA shows that compounds with positive charge that is not localized but covers most of the molecular surface are more retained (Supporting Information, Figure S5) which corresponds with interaction with protonated charged analytes observed for silica stationary phase in LSER.^14,21^ The effect of the number of hydrogen bond acceptors (nHBAcc) was more pronounced on the silica stationary phase. The presence of keto oxygens also decreased retention, especially on the BEH and diol column (khs.dO). In addition, other descriptors such as polarizability (BCUTp-1l), topological shape (topoShape), and simple cluster chi chain descriptor (SC-4) decreased the retention on the diol column by weights higher than those on silica and BEH columns. Retention on diol column was also affected by valence electrons as shown by HybRatio, which describes the fraction of sp^3^ carbons to sp^2^ carbons.

Overall, the retention on all three stationary phases increased with increasing values of several molecular descriptors that define the 3D structure of the analytes (Supporting Information, Table S4 and Figures S2–S4), such as molecular distance edge between oxygens, carbons, and nitrogens. That suggests that the branching of the molecule related to the secondary, tertiary, and quaternary carbons (MDEC-13 and MDEC-24) and higher number of functional groups with both primary (MDEO-11) and secondary oxygens (MDEO-22) and amines (MDEN-13) increases retention. The increasing effect of RNCS, i.e., relative negative charge surface area calculated as most negative surface area × RNCG (RNCG: relative negative charge–most negative charge/total negative charge) was observed. This confirms the decreasing effect of positive charge as expressed by RPCG and shows different preference for charge localization. While the delocalization of the positive charge increased the retention, the negative charge located on a specific part of the surface area, i.e., the most negative surface area, had an increasing effect on *t*_R_ (Supporting Information, Figures S5 and S6). High polar surface area also increased retention on silica and BEH columns, as shown by TPSA (total polar surface area) on BEH and RPSA (relative polar surface area) and tpsaEfficiency (polar surface area/molecular size) on silica.

However, some differences were also observed in the retention behavior on the three stationary phases. The retention on the silica column increased also with the number of basic groups in the molecule (nBase), which was also confirmed by the increasing number of –NH_2_ groups (khs.sNH2). That corresponds to the finding of Muteki et al.^28^ and Si-Hung et al.,^26^ who also reported strong affinity of silica stationary phases to basic compounds. Surprisingly, stronger interactions with bases were observed on BEH contrary to silica in LSER^14^ (Supporting Information, Figure S1B). However, in our study, nBase was the third most influential molecular descriptor on silica and only 48th on BEH. Aromatically bonded nitrogen atoms (khs.aaNH) increased the *t*_R_ on BEH instead of –NH_2_. On the other hand, the retention on BEH increased with the number of acidic groups (nAcid) in the molecule, which was also confirmed by the nHBDon descriptor that calculated the hydrogen bond donors in the molecule. nAcid had stronger effect on the retention on BEH as 11th molecular descriptor, but increased retention also on silica (24th) which corresponds with LSER results^14^ (Supporting Information, Figure S1B). The difference in retention mechanism could be explained by differences in silanol groups on the surface of silica versus BEH. Indeed, the –OH functionalities on bare silica are expected to be free and easily ionizable with p*K*_a_ around 4 – 7 in contrast to sterically hindered –OH on hybrid silica with p*K*_a_ around 10.^15,16^ Based on the molecular descriptors increasing retention, we hypothesize that –OH on the silica stationary phase behaves as an acid, i.e., as a proton donor. Thus, the retention is increased here when the analyte contains basic groups such as –NH_2_. On the other hand, –OH on the hybrid silica BEH stationary phase behaves more like a proton acceptor, resulting in increased retention of molecules with acidic groups and a high number of hydrogen bond donors. Similarly, the p*K*_a_ of propanediol on the diol column was estimated to be ≈ 14, corresponding to a similar retention mechanism as on BEH. Indeed, the nAcid was the 13th most influential molecular descriptor on diol whereas nBase was 87th. Therefore, the *t*_R_ of analytes with pronounced acidic groups increased in the row: silica > hybrid silica > diol as shown for six compounds in Supporting Information, Figure S7A. Conversely, the *t*_R_ of analytes with pronounced alkaline groups increased in diol > hybrid silica > silica (Supporting Information, Figure S7B).

Additionally, the retention on the diol column was mainly affected by the moment of inertia of the analytes (MOMI-XZ and MOMI-YZ) and the molecular framework (FMF). Both of these descriptors are related to the shape of the molecule. The FMF descriptor characterizes the complexity of a molecule, i.e., the ratio of heavy atoms in the framework to the total number of heavy atoms in the molecule. Acyclic molecules do not have frameworks and therefore have a value of 0 for FMF. This means that molecules with cycles in the structure are more strongly retained on the diol and BEH columns (Supporting Information, Table S4). Molecular descriptor topoShape describes the 2D shape of the molecule assigning value of 1 to acyclic molecules and values of 0 to cyclic molecules. Thus, the contrary weights assigned by ANN to FMF and topoShape confirmed stronger retention of cyclic molecules contrary to previous findings.^26^ The moment of inertia (MOMI) describes the 3D shape of the molecule. It is calculated based on three perpendicular axes passing through the center of mass and the mass distribution from these axes. Three descriptors, i.e., MOMI-X, MOMI-Y, and MOMI-Z, and their ratios can be calculated, describing four types of 3D shapes: linear molecules, symmetric top molecules, spherical molecules, and asymmetric top molecules. High values of MOMI-XZ and MOMI-YZ correspond to compounds with low mass distribution from the *z* axis, for example, prolate molecules, which will be more retained on diol column. A higher retention of spherical compounds was further confirmed by the molecular descriptor geomShape. This descriptor is also related to the 3D shape, and full circular/spherical compounds have value equal to 1 contrary to linear molecules with a value of 0.

This evaluation was based on the results obtained for 52 compounds eluting on all three stationary phases. However, 117 analytes were tested overall. Therefore, the differences caused by the higher affinity of the stationary phase for other analytes must be described for all individual columns.

Only five additional compounds eluted on the BEH column. All of them had an acidic character, further confirming the high affinity of BEH for analytes with acidic/H donor groups. This extended set of analytes was used for ANN evaluation and new weights were assigned to the molecular descriptors. Based on the calculated SD between the original and the new weights, most weights remained comparable with SD < 0.8 (Supporting Information, Figure S8A). The molecular descriptors with the largest weight changes are shown in Supporting Information, Figure S8B. The decreasing effect of keto oxygen (khs.dO) was reduced, but the decreasing effect of single bond oxygen (khs.ssO) became more pronounced. The negative effect of nHBAcc and molecular edge descriptors on retention became even stronger as did the increasing effect of –NH groups. The descriptors nAtomLC and nAtomLAC corresponding to the number of atoms in the largest chain and in the longest aliphatic chain, respectively, had opposite effects on the retention on BEH with long aliphatic chains increasing the retention. The molecular descriptors that positively and negatively affected the retention by highest valued weight are shown in Supporting Information, Table S4. In general, the descriptors increasing the retention remained mostly the same, i.e., MDEO-22, TPSA, nHBDon, the presence of –NH groups, and the 3D geometric shape of the molecule when focusing on anisotropy and sphericity (geomShape). Retention increased with a negative charge, but instead of RNCS, the retention is more affected by FNSA-3, i.e., a ratio of charge-weighted partial negative surface area to total molecular surface area. FNSA-1, i.e., partial negative surface area/total molecular surface area, had the opposite effect. This is simply due to the calculated values of these descriptors. In fact, FNSA-3 values are negative, meaning that the molecule with the highest charge-weighted partial negative surface area/total molecular surface area ratio was assigned values the lowest (most negative) values. In contrast, FNSA-1 is calculated in positive values. This again confirms that the coverage of the molecular surface by the negative charge plays a crucial role in the retention on the BEH column.

In contrast to BEH, 42 and 46 additional compounds were eluted on the silica and diol columns, respectively. These compounds include neutral molecules as well as molecules with acidic, basic, and/or both functional groups and are basically the same for both columns. Principal component analysis (PCA) of the two sets of analytes, i.e., the original set of 52 compounds and the additionally eluting compounds, did not reveal any significant differences between these two groups (Supporting Information, Figure S9). Since this addition nearly doubled the number of analytes used in the ANN calculations, significant changes in the molecular descriptor weights were expected. However, in case of the silica column, the main descriptors affecting retention remained the same. Retention decreased with the increasing number of H acceptors, –S– groups, and polar surface area. The *t*_R_ was further decreased with the presence of aromatic rings, methyl groups (C1SP3), and valence electrons (VC-5, VC-6, and VPC-6). On the other hand, increasing values of nBase, tpsaEfficiency, and aromatically bonded N increased the *t*_R_. Retention was also strongly affected by the value of the distance edge between oxygens and carbons, where the retention increased with the increasing distance.

The largest differences in molecular descriptor weights were observed on the diol column. The electron state remained the main parameter, decreasing the retention. The HybRatio as well as the chi chain descriptors of simple and valence clusters (SC, VC, and VP) decreased *t*_R_. However, the value of nHBAcc became a major parameter decreasing retention similarly to silica and BEH. The negative surface area of the molecule still increased the retention, although it was expressed more profoundly by FNSA-2 instead of the original RNCS. The weight of the Wlambda.unity parameter related to the molecular size along a principal axis increased, while the effect of MOMI decreased. The main difference was observed in the number of acidic and basic groups in the molecule. While the nAcid increased the retention in the original set of 52 compounds (13th descriptor), its effect became negligible (217th descriptor) and the nBase became a main parameter increasing the retention in the new set of 98 compounds (19th descriptor instead of 87th). This is mainly due to the addition of a higher number of compounds with acidic/basic properties and compounds containing both acidic and basic groups.

**Figure 2:**
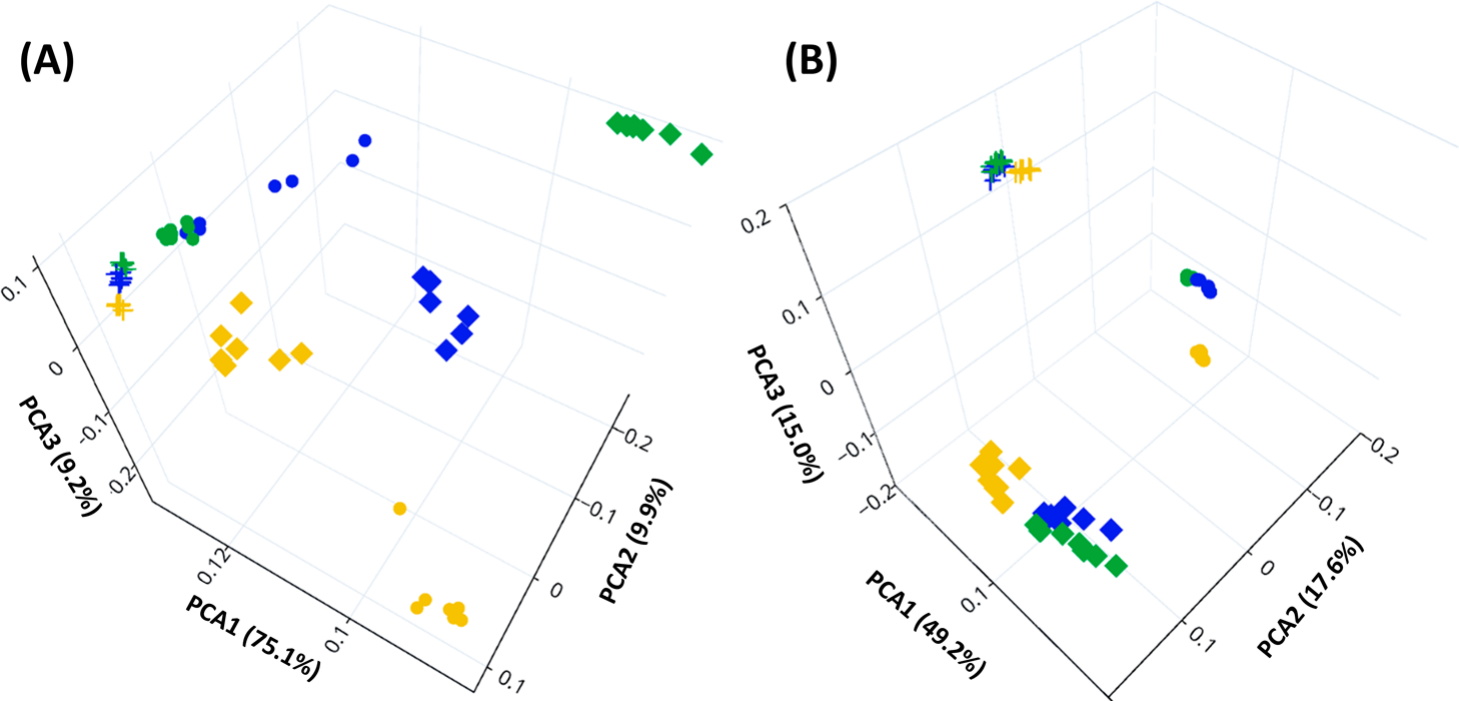
Principal component analysis of weights assigned by ANN to molecular descriptors based on (A) narrow and (B) extended set of compounds analyzed on silica (■), hybrid silica (●), and diol (×) stationary phase using MeOH (blue), MeOH + 2% H_2_O (green), and MeOH + 10 mmol/L NH_3_ (yellow). Multiple marks correspond to different data points.

To comprehensively compare the retention behavior of the tested stationary phases, PCA of the weights assigned to the molecular descriptors for each set of experimental data, i.e., stationary phase/organic modifier, was carried out (Figure 2). When the results obtained from the narrowed set of analytes were evaluated, the retention mechanisms of BEH and diol were similar, especially using MeOH and MeOH + H_2_O (Figure 2A). When the affinity of the stationary phase to specific analytes is included in the evaluation, the retention differed more significantly with three distinguished clusters (Figure 2B). Thus, only the results based on the extended sets are discussed in the following text to fully show the effect of the additive on each stationary phase.

### Effect of Additives on the Retention on Silica Stationary Phase

**Figure 3:**
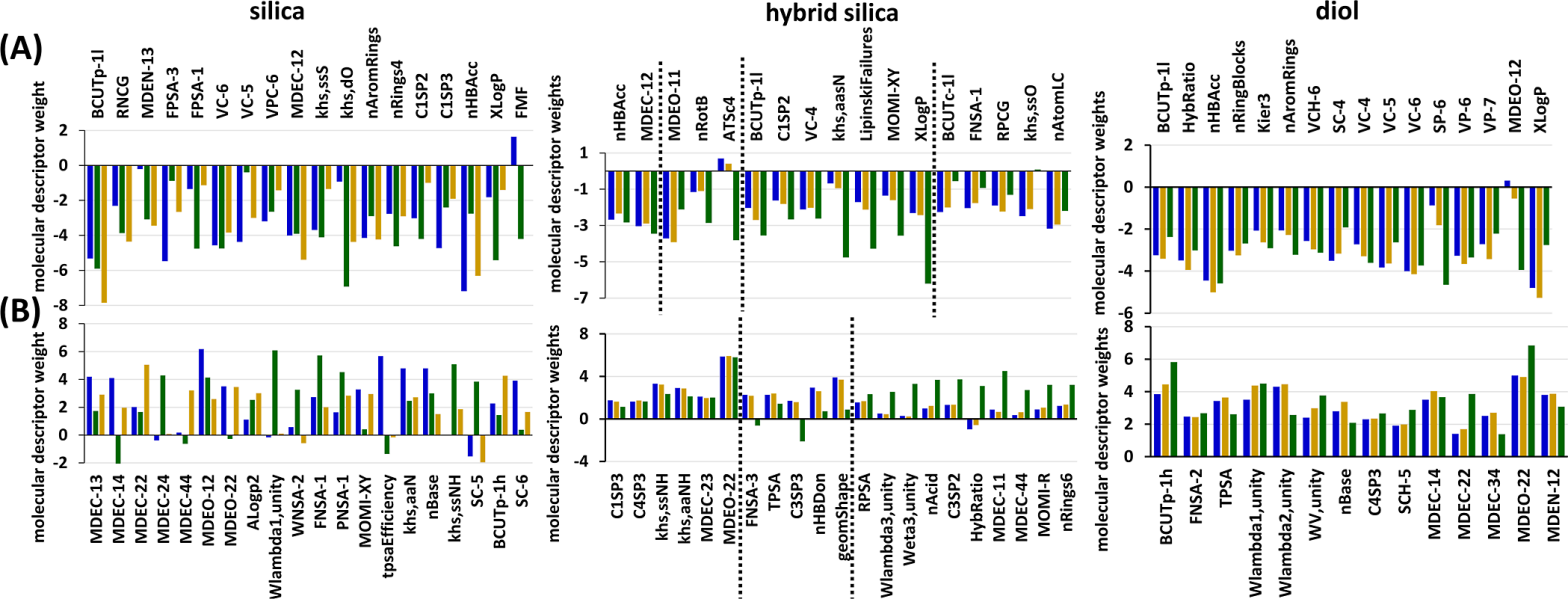
Molecular descriptor weights (A) decreasing and (B) increasing retention on tested columns determined by ANN, the organic modifier used: blue—MeOH, green—MeOH + 2% H_2_O, and yellow—MeOH + 10 mmol/L NH_3_.

The use of additives not only changes the apparent pH of the mobile phase but also can interact with the analytes and stationary phase surface. Thus, different retention interactions can be observed. That was true especially for silica stationary phase (Figure 2). The negative effect of several molecular descriptors became more pronounced when additives were used (Figure 3A). The decreasing effect of low polarizability (BCUTp-1l), relative negative charge (RNCG), and MDEN-13 increased in the following order: MeOH < H_2_O < NH_3_. In contrast to the negative charge (RNCG), molecular descriptors describing the positive surface area were more affected by the changes in the mobile phase composition. Indeed, FPSA-3 had a more significant negative effect on the retention when using pure MeOH and MeOH + NH_3_. On the other hand, the high value of FPSA-1 of the analyte decreased retention especially when MeOH + H_2_O was used. This strong effect of water addition on interactions of silica with positively charged analytes was shown also in LSER.^21^ FPSA-1 is calculated as partial positive surface area/total molecular surface area, which means that the high value of FPSA-1 corresponds to the molecules with most of the molecular surface area covered by partial positive charge. This positive surface is then available for interaction with H_2_O molecules in the mobile phase, resulting in lower retention.

The weights of VC-6, which provides information about the connectivity of various atoms in the molecule, and MDEC-12 related to the carbon connectivity remained basically unchanged. The effect of topological descriptors VC-5 and VPC-6 was more dependent on the composition of the mobile phase (Figure 3A). These descriptors account for the presence of heteroatoms in a molecule, as well as double and triple bonds, molecular size, degree of branching, and flexibility.^29^ The effect of covalently bonded S (–S–, khs.ssS) was similar in the case of MeOH and MeOH + H_2_O. However, its effect on retention decreased significantly when NH_3_ was added to the mobile phase. On the other hand, the presence of keto oxygen (khs.dO) had negligible effect on retention with MeOH, but significantly decreased the retention of analytes when H_2_O and/or NH_3_ were used in the mobile phase. Thus, an additive should be used in the mobile phase if the retention of compounds with keto functional groups needed to be decreased. The presence of aromatic rings (nAromRings) in the molecule decreased retention regardless of the mobile phase composition. However, its effect was lower using MeOH + H_2_O where the effect of four-atom rings was slightly more pronounced.

The effect of carbon connectivity in terms of hybridization, described by parameters C1SP2 (methylene, CH_2_=C-R) and C1SP3 (methyl, CH_3_=C-R), decreased significantly when NH_3_ was added to the mobile phase (Figure 3A). The addition of water mitigated the decreasing effect of the number of H-bond acceptors (nHBAcc). On the other hand, the negative effect of lipophilicity (*X*Log*P*) became more pronounced. The highest changes in the weight of important descriptors, decreasing retention on the silica column, were observed for FMF. FMF had a slightly positive effect on retention using MeOH. Its effect was canceled when water was added to the MeOH. In addition, the retention of molecules with high FMF values, i.e., cyclic compounds, decreased significantly when MeOH + NH_3_ was used in the mobile phase. Supporting Information Figure S10 shows a comparison of the *t*_R_ of the five analytes with the highest values of FMF in our set. The positive effect of FMF in case of MeOH is negligible compared to MeOH + H_2_O and other molecular descriptors play a more important role. However, the difference in FMF weights for MeOH and MeOH + NH_3_ was significant enough to relate to the decreased *t*_R_ using MeOH + NH_3_. Thus, MeOH + NH_3_ should be used to reduce the *t*_R_ of cyclic compounds.

The differences in weight of molecular descriptors increasing retention on silica stationary phase are shown in Figure 3B. The edge distances between carbons (MDEC) and oxygens (MDEO) played a significant role, and their effect was strongly dependent on the organic modifier. Similar dependence on mobile phase composition was observed for the simple cluster chi chain descriptors SC-5 and SC-6. The molecular descriptor of log*P*^2^ increased retention in the following order: MeOH < H_2_O < NH_3_. This means that strongly hydrophilic and strongly lipophilic compounds were more retained by using MeOH + NH_3_. High values of Wlambda.unity significantly increased retention using MeOH + H_2_O in contrast to other organic modifiers tested. Similarly, the effect of molecular descriptors of negative surface, namely, WNSA-2 and FNSA-1, was more pronounced when using MeOH + H_2_O. The strong dependence of retention on silica on the negative surface area was further confirmed by PNSA-1. The apparent pH of ≈ 1 of the CO_2_/MeOH/H_2_O mobile phase causes the silanols with p*K*_a_ ≈ 4 – 7 to be mostly in –OH form and thus strongly interact with analytes having negative surface. The negative surface area of the molecule increased its retention on the silica column. However, the distribution of the negative parts of the molecule affected the retention behavior differently when using different organic modifiers.

The presence of water in the organic modifier reduced the effect of the moment of inertia of the molecule (MOMI-XY, Figure 3B). The polar surface area, expressed as tpsaEfficiency, was a critical parameter of the molecule, increasing its retention on the silica column using MeOH. However, its effect was completely negated by the additive. Similarly, the presence of the additive decreases the weights of the khs.aaN descriptor counting the aromatically bonded nitrogens and the number of basic groups in the molecule (nBase). On the other hand, molecules with the covalently bonded –NH– in them (khs.ssNH) had significantly higher retention when using additives and especially H_2_O. At first glance, the opposite behavior of the descriptors khs.ssNH and nBase seems strange. However, the khs.ssNH descriptor calculates all covalently bonded –NH groups regardless of other atoms bonded to the carbons. Thus, both the secondary amino group and the amide group are counted within this parameter. On the other hand, nBase counts only groups with alkaline properties, i.e., in this case, the secondary amino groups (Supporting Information, Figure S11). Hence, the presence of –NH plays a more important role when using an organic modifier with an additive regardless of the alkaline properties of the –NH groups.

### Effect of Additives on the Retention on Hybrid Silica BEH Stationary Phase

Based on the differences in weights caused by using additives, the molecular descriptors decreasing *t*_R_ on hybrid silica column can be divided into four groups (Figure 3A): (i) nHBAcc and MDEC-12 were not affected by the change in the mobile phase composition and their weights remained similar regardless of the organic modifier used. (ii) The weights and thus the effect of MDEO-11, nRotB, and ATSc4 were similar when using MeOH and MeOH + H_2_O. However, significant changes were observed when MeOH + NH_3_ was used. The decreasing effect of the number of rotatable bonds (nRotB) on the retention became more pronounced with MeOH + NH_3_ in contrast to MDEO-11 (Figure 3A). The Moreau–Broto autocorrelation descriptors using partial charges (ATSc4) had slightly positive but mostly negligible effect on the retention with MeOH and MeOH + H_2_O. The retention of the molecule with a high value of ATSc4 was significantly decreased when using MeOH + NH_3_ (Figure 3A). (iii) The molecular descriptors with increasing weights in the order of MeOH < H_2_O < NH_3_ included BCUTp-1l, C1SP2, VC-4, khs.aasN, LipinskiFailure, MOMI-XY, and *X*Log*P*. Here, the changes between MeOH and MeOH + H_2_O were significantly less pronounced compared to MeOH + NH_3_. Thus, oblate and asymmetric (MOMI-XY) lipophilic (log*P*) molecules with methylene groups and aromatic rings with tertiary nitrogen will be more strongly retained using MeOH as opposed to MeOH + NH_3_. (iv) In contrast, the effect of BCUTc, RPCG, and FNSA-1 related to the charge, the number of covalently bonded oxygens (–O–, khs.ssO), and the number of atoms in the largest chain (nAtomLC), was more pronounced for MeOH > H_2_O > NH_3_. Again, the difference was observed especially for MeOH + NH_3_ contrary to MeOH and MeOH + H_2_O. Thus, molecules with highly chargeable and negative surface area with –O– parts in the molecule and long chain are retained more strongly on BEH using MeOH + NH_3_ compared to MeOH. Indeed, the effect of –O– parts in the molecule on *t*_R_ was strongly negative using MeOH and MeOH + H_2_O. However, this negative effect was substantially mitigated by the addition of NH_3_ (Figure 3A).

Only minor differences were observed in the weights of molecular descriptors, increasing retention on the BEH stationary phase when using MeOH and MeOH + H_2_O, while the addition of NH_3_ significantly changed the retention mechanism (Figure 3B). The presence of covalently and aromatically bonded NH (khs.ssNH and khs.aaNH), distance edges between secondary carbons and oxygens (MDEC-23 and MDEC-22), and the presence of C1SP3 and C4SP3 in the molecule had a similar effect on the retention using all three organic modifiers. The addition of NH_3_ to the organic modifier reduced the effect of TPSA, nHBDon, and geomShape. Indeed, the positive effect of the spherical shape of the molecule on the retention observed for MeOH and MeOH + H_2_O was mitigated by MeOH + NH_3_. The use of MeOH + NH_3_ also resulted in the switch from positive to negative effects of molecular descriptors FNSA-3 and C3SP3. In contrast, the effect of the molecular descriptors in group 3 in Figure 3B became more pronounced with MeOH + NH_3_. Retention was more affected by nAcid when using MeOH + NH_3_. These acidic groups may interact with the NH_3_ ions in the mobile phase. Subsequently, the analyte-NH_3_ adducts can interact with silanol, resulting in higher retentivity of analytes. The use of MeOH + NH_3_ also increased the effect of HybRatio, MDEC-11, and MDEC-44. Overall, the retention mechanism on BEH was similar using MeOH and MeOH + H_2_O, whereas the use of MeOH + NH_3_ resulted in different interactions (Figure 2).

### Effect of Additives on the Retention on Diol Stationary Phase

In contrast to silica and BEH stationary phases, the weights of the molecular descriptors decreasing the retention on the diol column were less affected by the organic modifier composition used (Figure 3A) corresponding to a similar retention mechanism (Figure 2). The only notable changes were in the parameters SP-6, a simple path of order 6, and the distance edge between primary and secondary oxygens (MDEO-12). Their negative effect on retention increased with the addition of NH_3_. The increasing effect of polarizability (BCUTp-1h) increased with the use of additives, similarly to the parameters MDEC-22 and MDEO-22.

Overall, although all three tested stationary phases contain –OH functionalities, the effect of the used organic modifier varies. We assume that this variance can be correlated to the p*K*_a_ of the –OH functionalities. The –OH functionalities on the silica surface have p*K*_a_ values of around 4 – 7 and thus are more sensitive to the changes in mobile phase pH. Indeed, we observed significant differences in the retention behavior with all three organic modifiers. On the other hand, the retention on hybrid silica BEH with –OH functionalities with p*K*_a_ ≈ 10 was similar for MeOH and MeOH + H_2_O, and significant changes were observed only in the case of MeOH + NH_3_. The p*K*_a_ of –OH functionalities on the diol column is ≈ 14, which means that their ionization state should be stable. Thus, we observed very similar retention behavior with close weights for most of the molecular descriptors.

### Changes in Retention Over Time

The stability of the molecular descriptor weight over time was determined to indicate changes in retention mechanisms. Based on the stationary phase chemistry, diol columns were expected to have the most stable *t*_R_ over time. This was confirmed by the % *t*_R_ shifts, which were mostly < 5% even after 12M. Overall, mostly increase in *t*_R_ was observed after 6M regardless of the organic modifier (Supporting Information, Figure S12). This shows that SEF is not responsible for the *t*_R_ shifts as SEF typically causes decreases of *t*_R_.^6^ The stable *t*_R_ was expected on the diol column since the free silanols are shielded by the propanediol linker and the diol –OH functionalities are quite stable with p*K*_a_ 14 and not susceptible to the reaction with the MeOH forming silyl ethers. A closer look at the weights of the molecular descriptors over time shows that most of them, and thus, the interactions affecting retention, remained stable over 12M and even after the regeneration procedure with SD < 0.66. This lowest obtained SD value of 0.66 was considered as a limit for a stable weight of molecular parameters over time for other columns. The molecular descriptors with the most variable weights over time are shown in Supporting Information, Figure S13. No trend was observed, only small fluctuations close to the same value. That corresponds with the overall evaluation in Figure 2 where all data points clustered closely together.

BEH column contains –OH functionalities with p*K*_a_ around 10 and ethylene bridges. Thus, their susceptibility to SEF should be lower compared to that of the conventional silica stationary phase. Mostly decreased *t*_R_ were observed over the year of measurements, especially with MeOH + NH_3_ (Supporting Information, Figure S14). Using MeOH, only two molecular descriptors khs.sNH2 and MDEO-22 changed significantly with SD values of 0.93 and 1.06, respectively. The addition of NH_3_ to the mobile phase further stabilized the retention mechanism, as all molecular descriptors had stable weights over the year with SD < 0.30. The use of MeOH + H_2_O resulted in similar stability with SD < 0.29. The differences in the weights of these molecular descriptors at all data points are shown in Supporting Information, Figure S15. The effect of khs.sNH2 on retention with MeOH + H_2_O is negligible with weights <1. Therefore, its fluctuation over time has no real effect on the retention. Khs.sNH2 has the opposite effect on the retention on the BEH column using MeOH and MeOH + NH_3_. Its decreasing effect when MeOH + NH_3_ was used was stable over time. On the other hand, khs.sNH2 had a small increasing effect on retention using MeOH at 0M and 1M. Subsequently, its effect increased more than twofold. This suggests that a change on the surface of the stationary phase occurred between 1 and 2M. This change resulted in more sites on the stationary phase surface available for interactions with –NH_2_ groups. MDEO-22 had a positive effect on retention with all three organic modifiers. While this effect remained the same over time using MeOH + H_2_O and MeOH + NH_3_, it gradually decreased when using MeOH. MDEO-22 shows that the retention increased with the larger distance edge between the secondary oxygens. This suggests that analytes with secondary oxygens at different parts of the molecule interacted more strongly with the stationary phase. However, the number of sites for this interaction decreased over time using MeOH.

Looking at individual analytes in more detail, compounds with strongly acidic or alkaline properties were most affected by changes in the stationary phase surface over time. Indeed, a strong decrease in retention was observed for acidic compounds contrary to a strong increase of retention for alkaline compounds (Supporting Information Figure S16). Several differences were observed in molecular descriptors between these two groups. A significant change was observed for DPSA-1, which described the difference between partial positive surface and partial negative surface. As expected, the acidic compounds had lower DPSA-1 values, all < 66, in contrast to DPSA-1 values for the alkaline group, all > 275. This means that compounds with mostly positive surface area were more strongly retained over time suggesting that –OH functionalities were more available for the interactions. Other strongly differing molecular descriptors included the Moreau–Broto autocorrelation descriptors using partial charges (ATSc3 and ATSc5), methyl groups (C1SP3), and especially HybRatio. We conclude that the acidic group contained more sp^2^ carbons since the value of HybRatio was < 0.13 and > 0.32 in the acidic and alkaline group, respectively. However, the average value of the whole set of analytes was 0.38 and even compounds with > 0.8 were included. Therefore, it could not be marked as a differing descriptor between the discussed groups.

The use of a silica stationary phase resulted in the most unstable *t*_R_. Using MeOH, MeOH + H_2_O, and MeOH + NH_3_, SD of molecular descriptor weights > 0.66 were observed for 45, 46, and 11 descriptors, respectively. This suggests that the surface of the silica stationary phase changes over time, resulting in changes in retention behavior. Overall, a decrease in *t*_R_ over time was observed for most of the analytes (Supporting Information, Figure S17). This can be correlated with the possibility of SEF, in which case fewer –OH groups were available to interact with the analytes, resulting in reduced retention. The addition of water to the organic modifier did not have a positive effect on the *t*_R_ stability (Figure 1). Furthermore, from the comparison shown in Figure 1, it appears that the largest *t*_R_ shift occurred between the 0M and 1M, followed by fairly stable *t*_R_. In fact, when the *t*_R_ shifts were calculated from the *t*_R_ at 1M as 100% as opposed to the original first injection (0M), the stability was quite different (Supporting Information, Figure S18). This suggests that the equilibration procedure used for new columns was not sufficient for this specific silica stationary phase, contrary to diol and BEH. As a consequence, we suggest to use at least two times more column volumes to flush and cover the silica stationary phase with the organic modifier than previously recommended for SFC-dedicated columns.^12^ It seems that even after using the postanalysis washing procedure, the stationary phase surface remained covered by the organic modifier and, more importantly, by the additive. Indeed, the same equilibration protocol was then sufficient to ensure repeatable retention in the following data points (Supporting Information, Figure S18). The most stable retention over time was observed with the MeOH + NH_3_. These results suggest that the silanols on the surface of the stationary phase were indeed covered by NH_3_ ions even during storage and were not available for the SEF. The molecular descriptors with the highest weights change over time are shown in Supporting Information, Figure S19 for all three organic modifiers.

### Effect of Regeneration Procedure

The regeneration procedure should restore the stationary phase and return it to its original state. The regeneration procedure was quite effective on the BEH and diol column, where over 79% of compounds had *t*_R_ within ±2% of the original *t*_R_ (Supporting Information, Figure S20). However, these two stationary phases also had significantly more stable *t*_R_ values over time. The *t*_R_ value at 12M must also be considered to properly determine the effectiveness of the regeneration procedure. The *t*_R_ on the diol column were mostly unaffected by the regeneration or even worsened as in the case of MeOH (Supporting Information, Figure S21). There were only three cases where the regeneration had a significant beneficial effect: (i) silica column with MeOH + NH_3_. However, the shift in the *t*_R_ after the regeneration is probably caused by the washing out of NH_3_ ions from the silanols. Thus, after another equilibration and measurement sequence, the *t*_R_ shifted back to those observed in 2M–12M. (ii) Similar behavior was also observed on the BEH column. Here, the difference between *t*_R_ at 1M versus 2M was not as critical as in case of the silica column, but it was still significant for over 50% of the compounds. Thus, we expect a similar behavior after another measurement series, as on silica column. As opposed to MeOH + NH_3_, the obtained with MeOH and MeOH + H_2_O on BEH remained mostly unaffected by the regeneration procedure. The value of *t*_R_ is not the only chromatographic parameter that can be affected by changes in the surface of the stationary phase. (iii) The beneficial effect of regeneration was also observed for peak widths on silica and BEH stationary phases, independently of the organic modifier used. Most peaks were narrower after regeneration than at 12M. The same was true for the diol column when using MeOH. This suggests that the column efficiency improved.

Nevertheless, the regeneration procedure did not meet expectations, as it did not return the stationary phase to its original state, and the results obtained during the first injection could not be completely reproduced. Thus, further research and a suggestion for a modified regeneration procedure are needed to ensure the reproducibility of the SFC methods over time.

## Conclusions

ANN have been used for the first time to comprehensively define compound properties expressed as molecular descriptors responsible for retention in SFC, specifically on polar stationary phases with predominant –OH functionalities. The key molecular descriptors affecting the retention to the highest extent were defined separately for three different organic modifiers. Overall, the retention behavior on all tested columns could be correlated with the p*K*_a_ of the respective –OH functionalities and the apparent pH of the SFC mobile phase based on the organic modifier used. For the first time, we also quantitatively described the changes in the interactions when using 10 mmol/L NH_3_ and 2% water as additives compared to pure methanol as an organic modifier. For the hybrid silica column, a high retention of analytes with acidic groups, H bond donor groups, –NH, and a negative charge was observed. The coverage of the molecular surface by the negative charge and its localization played a crucial role in the retention behavior. Changing the organic modifier resulted in significant changes in molecular descriptor weights, especially when using MeOH + NH_3_ compared to pure MeOH. Even stronger effect of additive was observed on the silica column. Several important molecular descriptors including the presence of keto oxygen, the number of H bond acceptors, lipophilicity, the ratio of heavy atoms in the framework to the total number of heavy atoms in the molecule, negative surface, and especially –NH groups, and the number of basic groups, played an important role in describing retention behavior on silica using different organic modifiers. For the diol column, the number of H bond acceptor decreased the retention, while it increased with increasing negative surface area and number of basic groups. Here, the retention was significantly less affected by changing the organic modifier.

The detailed description of retention interactions enables the selection of a suitable organic modifier for increasing and/or decreasing retention of particular analytes. This fundamental understanding of interactions responsible for retention in SFC can be used for the separation of analytes based on their properties. Thus, a lower number of time-consuming experiments will be necessary for the development of SFC methods, further increasing the environmental friendliness of the SFC technique.

The best stability of *t*_R_ over one year of use was observed for a diol column with –OH functionalities not prone to SEF. For the BEH column, mostly increased *t*_R_ values were observed. The addition of NH_3_ and/or H_2_O to the mobile phase further stabilized the retention. However, a strong decrease in retention was observed for acidic compounds, in contrast to a strong increase in retention for alkaline compounds. The highest instability of *t*_R_ was observed on the silica column with a predominant decrease in *t*_R_ over time. This can be correlated with the possibility of SEF, as in this case, less –OH is available for the interactions, resulting in lower retention. In addition, the need for longer equilibration was noted when using an organic modifier with an additive on a silica column. A combination of SEF and additive adsorption on the stationary phase surface was responsible for the column aging over time when using MeOH + NH_3_. The regeneration procedure used did not have a significant positive effect on the *k*′ but had a positive effect on peak width, especially on the BEH column. Nevertheless, the regeneration procedure did not meet the expectations, as it did not return the stationary phase to its original state, and the results obtained at the first injection could not be reproduced.

## Data Availability

The original data used in this publication are openly available in Zenodo under the DOI: 10.5281/zenodo.12707608.

## Supporting Information Available

List of analytes; list of molecular descriptors; chemical structures and LSER parameters; descriptors with the highest effect on the retention; heatmaps of molecular descriptor weights; illustration of RPCG, RHSA, RNCG, and RNCS; retention of acidic and alkaline compounds; SDs of molecular descriptor weights on BEH; retention time of analytes with the highest values of FMF; chemical structure of acebutolol; percentage of analytes with increased/decreased *t*_R_ over time; weights of khs.sNH2 and MDEO-22 descriptors determined by ANN on the BEH column; comparison of *t*_R_ shifts of alkaline and acidic compounds on the BEH column; weights of the molecular descriptors most affected over time on diol and silica columns; and comparison of retention time shifts over time on the silica column (PDF)
