# Supplementary material for: Advancing Fundamental Understanding of Retention Interactions in Supercritical Fluid Chromatography Using Artificial Neural Networks: Polar Stationary Phases with –OH Moieties
Source: Anal Chem. 2024 Jul 29;96(31):12748–59. doi: 10.1021/acs.analchem.4c01811 (PMC11307250; doi:10.1021/acs.analchem.4c01811)
Supplement: Supplementary file 1 — ac4c01811_si_001.pdf [file ac4c01811_si_001.pdf]

Supporting Information: Advancing Fundamental Understanding of Retention Interactions in Supercritical Fluid Chromatography Using Artificial Neural Networks: Polar Stationary Phases with -OH moieties

Kateřina Plachká[1], Veronika Pilařová[1], Taťána Gazárková[1], František Švec[1], Jean-Christophe Garrigues[2], Lucie Nováková[1*]

[1] Department of Analytical Chemistry, Faculty of Pharmacy in Hradec Králové, Charles University, 500 05Hradec Králové, Czechia

2 SOFTMAT (IMRCP) Laboratory, SMODD Team, CNRS, Toulouse III Paul Sabatier University, 31400 Toulouse, France

* Email: nol@email.cz (Lucie Nováková)

S1. Experimental Part

Table S1: List of analytes; their physicochemical properties and elution on tested columns (BEH, silica, diol) using three tested organic modifiers, namely MeOH, MeOH + 10 mM NH_3, and MeOH + 2% H_2O.

Table S2: Molecular descriptors used in the study, calculated with CDK Descriptor Calculator (v.1.4.8).

Table S3: Molecular descriptors used in the study grouped by primary category.

Figure S1. (A) The chemical structure of tested -OH stationary phases, (B) the effect of LSER parameters on silica, BEH, and diol columns, and (C) a spider diagram characterizing stationary phases based on LSER.

S2. Results

Table S4. Molecular descriptors with the highest effect on the retention of compounds identified from the weights assigned by the ANNs after 500 training cycles.

Figure S2. Heatmap of molecular descriptor weights representing their effect on the retention on silica column.

Figure S3. Heatmap of molecular descriptor weights representing their effect on the retention on hybrid silica.

Figure S4. Heatmap of molecular descriptor weights representing their effect on the retention on diol column.

Figure S5. General illustration of molecules with high and low values of RPCG and RHSA.

Figure S6. General illustration of molecules with high and low values of RNCG and RNCS.

Figure S7. Retention times of selected acidic and alkaline compounds on silica, BEH, diol column using MeOH.

Figure S8. (A) Histogram of standard deviations (SD) between weights of molecular descriptors calculated by ANN using the original and extended set of analytes measured on BEH column using MeOH as organic modifier. (B) Comparison of weights of molecular descriptors with the highest SD.

Figure S9. Principal Component Analysis of original set of 52 compounds (blue) and additional compounds eluting on silica and diol column (red).

Figure S10. Retention time of the 5 analytes with the highest values of FMF measured on silica column using MeOH (blue), MeOH+2%H_2O (yellow), and MeOh+10 mmol/L NH_3 (green).

Figure S11. Chemical structure of acebutolol with highlighted -NH- groups.

Figure S12. Percentage of analytes with increased (purple) and decreased (blue) retention times at selected data points when compared to the 1st injection on diol column.

Figure S13. Comparison of weights of the molecular descriptors most affected over time on diol column.

Figure S14. Percentage of analytes with increased (purple) and decreased (blue) retention times at selected data points when compared to the 1st injection on BEH column.

Figure S15. Weights of khs,sNH2 and MDEO-22 molecular descriptors determined by ANN based on the analyses on BEH column at the 1st injection (0M), after 1, 2, 3, 6, 9, and 12 months (M), and after regeneration (R).

Figure S16. Comparison of retention time shifts over time for alkaline and acidic compounds analyzed on a BEH column using MeOH as organic modifier.

Figure S17. Percentage of analytes with increased (purple) and decreased (blue) retention times at selected data points when compared to the 1st injection on silica column.

Figure S18. Comparison of retention time shifts over time on silica column using (A) methanol, (B) MeOH+10 mmol/L NH_3, and (C) MeOH+ 2% H_2O as organic modifier.

Figure S19. Comparison of weights of the molecular descriptors most affected over time on silica column.

Figure S20. Comparison of % errors calculated between tR after regeneration and tR at the first injection on silica, BEH, and diol columns. %-error less than 0.5% (dark blue), 0.5 – 1.0% (light blue), 1.0 -2.0% (yellow), 2.0 -5.0% (pink), and over 5% (red).

Figure S21: Comparison of changes in retention time and peak width after regeneration procedure. k′ closer to the value of the first injection after regeneration (blue) vs. after 12 months (red).

S1. Experimental part

Table S1: List of analytes; their physicochemical properties, including molecular weight (MW), acidic/basic properties (pKa), lipophilicity (logP), number of H acceptors and donors, and molecular formula; and elution on tested columns (BEH, silica, diol) using three tested organic modifiers, namely MeOH, MeOH + 10 mM NH_3, and MeOH + 2% H_2O. n/a means, that analyte did not elute on any of the columns.

n.	CAS No.	analyte	Mw	pKa acid	pKa basic	log P	H acc.	H donor	molecular formula	MeOH	MeOH + 10 mM NH_4	MeOH + 2% H_2O
**1**	841-67-8	(-)-thalidomide	258.23	10.7	-2.55	0.33	1	7	$C_{13}H_{10}N_2O_4$	BEH; silica; diol	BEH; silica; diol	BEH; silica; diol
**2**	2614-06-4	(+)-thalidomide	258.23	10.7	-2.55	0.33	1	7	$C_{13}H_{10}N_2O_4$	BEH; silica; diol	BEH; silica; diol	BEH; silica; diol
**3**	303-38-8	2,3-dihydroxy benzoic acid	154.12	2.96	-	1.62	4	3	$C_7H_6O_4$	silica	BEH; silica	n/a
**4**	89-86-1	2,4-dihydroxy benzoic acid	154.12	3.32	-	1.77	4	3	$C_7H_6O_4$	silica; diol	BEH; silica; diol	silica; diol
**5**	490-79-9	2,5-dihydroxy benzoic acid	154.12	3.01	-	1.40	4	3	$C_7H_6O_4$	silica; diol	BEH; silica; diol	silica; diol
**6**	303-07-1	2,6-dihydroxy benzoic acid	154.12	1.3	-	2.38	4	3	$C_7H_6O_4$	n/a	BEH; silica; diol	n/a
**7**	583-17-5	2-hydroxy cinnamic acid	164.16	4.51	-	1.02	3	2	$C_9H_8O_3$	BEH; silica; diol	BEH; silica; diol	BEH; silica; diol
**8**	362-07-2	2-methoxyestradiol	302.41	10.29	-	3.84	3	2	$C_{19}H_{26}O_3$	BEH; diol	BEH; silica; diol	BEH; diol
**9**	99-50-3	3,4-dihydroxy benzoic acid	154.12	4.45	-	1.01	4	3	$C_7H_6O_4$	silica; diol	n/a	silica; diol
**10**	99-10-5	3,5-dihydroxy benzoic acid	154.12	3.96	-	0.81	4	3	$C_7H_6O_4$	BEH; diol	BEH; silica; diol	BEH; silica; diol
**11**	530-59-6	4-hydroxy-3,5-dimethoxy-cinnamic acid	224.21	4.53	-	1.00	5	2	$C_{11}H_{12}O_5$	BEH; silica; diol	BEH; silica; diol	BEH; silica; diol
**12**	588-30-7	3-hydroxy cinnamic acid	164.16	4.38	-	0.93	3	2	$C_9H_8O_3$	BEH; silica; diol	BEH; silica; diol	BEH; silica; diol
**13**	537-73-5	3-Hydroxy-4-methoxycinnamic acid	194.18	4.53	-	0.79	4	2	$C_{10}H_{10}O_4$	BEH; diol	BEH; diol	BEH; silica; diol
**14**	530-57-4	4-hydroxy-3,5-dimethoxybenzoic acid	198.17	4.33	-	1.28	5	2	$C_9H_{10}O_5$	BEH; silica; diol	BEH; silica; diol	BEH; silica; diol
**15**	121-34-6	4-hydroxy-3-methoxy benzoic acid	168.15	4.45	-	1.30	4	2	$C_8H_8O_4$	BEH; silica; diol	BEH; silica; diol	BEH; silica; diol
**16**	99-96-7	4-hydroxybenzoic acid	138.12	4.57	-	1.40	3	2	$C_7H_6O_3$	BEH; silica; diol	BEH; silica; diol	BEH; silica; diol
**17**	100-09-4	4-methoxy benzoic acid	152.15	4.47	-	1.78	3	1	$C_8H_8O_3$	BEH; silica; diol	BEH; silica; diol	silica; diol
**18**	154229-18-2	abiraterone acetate	391.55	5.31	-	6.58	3	0	$C_{26}H_{33}NO_2$	silica; diol	BEH; silica; diol	BEH; silica; diol
**19**	37517-30-9	acebutolol	336.43	13.78	9.4	1.77	6	3	$C_{18}H_{28}N_2O_4$	silica; diol	BEH; silica; diol	silica; diol
**20**	138112-76-2	agomelatine	243.3	16.17	-0.53	2.47	3	1	$C_{15}H_{17}NO_2$	BEH; silica; diol	BEH; silica; diol	BEH; silica; diol

n.	CAS No.	analyte	Mw	pKa acid	pKa basic	log P	H acc.	H donor	molecular formula	MeOH	MeOH + 10 mM NH_4	MeOH + 2% H_2O
**21**	52-39-1	aldosterone	360.44	12.98	-	0.70	2	7	$C_{21}H_{28}O_5$	BEH; silica; diol	BEH; silica; diol	BEH; silica; diol
**22**	59-02-9	alpha-tocopherol	430.71	11.4	-	10.96	2	1	$C_{29}H_{50}O_2$	silica; diol	BEH; silica; diol	BEH; silica; diol
**23**	1721-51-3	alpha-tocotrienol	424.66	11.4	-	9.70	2	1	$C_{29}H_{44}O_2$	BEH; silica; diol	BEH; silica; diol	silica; diol
**24**	520-36-5	apigenin	270.24	6.53	-	2.13	5	3	$C_{15}H_{10}O_5$	silica; diol	BEH; silica; diol	silica; diol
**25**	29122-68-7	atenolol	266.34	13.88	9.43	0.36	5	4	$C_{14}H_{22}N_2O_3$	silica; diol	BEH; silica; diol	silica; diol
**26**	98-55-5	a-terpineol	154.25	14.94	-	2.54	1	2	$C_{10}H_{18}O$	BEH; silica; diol	BEH; silica; diol	BEH; silica; diol
**27**	83015-26-3	atomoxetine	255.35	10.15	-	3.36	2	1	$C_{17}H_{21}NO$	silica; diol	BEH; silica; diol	BEH; silica; diol
**28**	134523-00-5	atorvastatin	558.64	4.29	0.38	3.85	7	4	$C_{33}H_{35}FN_2O_5$	silica; diol	silica; diol	silica; diol
**29**	148-03-8	beta tocopherol	416.68	11.05	-	10.72	2	1	$C_{28}H_{48}O_2$	silica; diol	silica; diol	silica; diol
**30**	490-23-3	beta tocotrienol	410.63	11.05	-	9.46	2	1	$C_{28}H_{42}O_2$	silica; diol	silica; diol	silica; diol
**31**	378-44-9	betamethasone	392.46	12.13	-	2.03	5	3	$C_{22}H_{29}FO_5$	BEH; silica; diol	BEH; silica; diol	BEH; silica; diol
**32**	62658-63-3	bopindolol	380.48	17.59	9.4	4.82	5	2	$C_{23}H_{28}N_2O_3$	BEH; silica; diol	BEH; silica; diol	silica; diol
**33**	51-20-7	bromo uracil	190.98	6.77	-	-0.21	4	2	$C_4H_3BrN_2O_2$	BEH; silica; diol	BEH; silica; diol	BEH; silica; diol
**34**	58-08-2	caffein	194.19	0.52	-	-0.63	6	0	$C_8H_{10}N_4O_2$	BEH; silica; diol	BEH; silica; diol	BEH; silica; diol
**35**	154-23-4	catechin	290.27	9.54	-	0.61	5	11	$C_{15}H_{14}O_6$	silica; diol	silica; diol	silica; diol
**36**	83881-51-0	cetirizin	388.89	3.46	6.71	1.62	5	1	$C_{21}H_{25}ClN_2O_3$	silica; diol	BEH; silica; diol	BEH; silica; diol
**37**	50-22-6	cortikosterone	346.46	12.98	-	1.95	2	6	$C_{21}H_{30}O_4$	BEH; silica; diol	BEH; silica; diol	BEH; silica; diol
**38**	71-30-7	cytosine	111.1	9	4.18	-1.96	4	3	$C_4H_5N_3O$	silica; diol	BEH; silica; diol	BEH; silica; diol
**39**	1009119-64-5	daclatasvir	738.88	10.92	6.56	2.51	14	4	$C_{40}H_{50}N_8O_6$	BEH; silica; diol	BEH; silica; diol	BEH; silica; diol
**40**	133099-04-4	darifenacin	426.55	15.7	9.32	3.78	4	2	$C_{28}H_{30}N_2O_2$	BEH; silica; diol	BEH; silica; diol	BEH; silica; diol
**41**	302962-49-8	dasatinib	488.01	10.94	7.29	0.14	9	3	$C_{22}H_{26}ClN_7O_2S$	silica; diol	BEH; silica; diol	silica; diol
**42**	53-43-0	dehydro-epi-androsterone	288.43	15.02	-	3.31	1	3	$C_{19}H_{28}O_2$	BEH; silica; diol	BEH; silica; diol	BEH; silica; diol

n.	CAS No.	analyte	Mw	pKa acid	pKa basic	log P	H acc.	H donor	molecular formula	MeOH	MeOH + 10 mM NH_4	MeOH + 2% H_2O
**43**	119-13-1	delta tocopherol	402.65	10.7	-	10.49	2	1	$C_{27}H_{46}O_2$	silica; diol	silica; diol	silica; diol
**44**	25612-59-3	delta tocotrienol	396.61	10.69	-	9.23	2	1	$C_{27}H_{40}O_2$	BEH; silica; diol	BEH; silica; diol	silica; diol
**45**	50-47-5	desipramin	266.38	-	10.4	3.97	2	1	$C_{18}H_{22}N_2$	BEH; silica; diol	BEH; silica; diol	BEH; silica; diol
**46**	50-02-2	dexamethasone	392.46	12.13	-	2.03	5	3	$C_{22}H_{29}FO_5$	BEH; silica; diol	BEH; silica; diol	BEH; silica; diol
**47**	915087-33-1	enzalutamide	464.44	13.88	-1.99	2.98	6	1	$C_{21}H_{16}F_4N_4O_2S$	BEH; silica; diol	BEH; silica; diol	BEH; silica; diol
**48**	57-91-0	estradiol	272.38	10.27	-	4.15	2	2	$C_{18}H_{24}O_2$	BEH; silica; diol	BEH; silica; diol	BEH; silica; diol
**49**	4245-41-4	estradiol acetate	314.42	15.06	-	4.46	3	1	$C_{20}H_{26}O_3$	BEH; silica; diol	BEH; silica; diol	BEH; silica; diol
**50**	50-27-1	estriol	288.38	10.25	-	2.53	3	3	$C_{18}H_{24}O_3$	BEH; silica; diol	BEH; silica; diol	BEH; silica; diol
**51**	53-16-7	estron	270.37	10.25	-	3.62	2	1	$C_{18}H_{22}O_2$	BEH; silica; diol	BEH; silica; diol	BEH; silica; diol
**52**	774-40-3	(±)-ethyl mandelate	194.23	12.31	-	1.76	3	1	$C_{10}H_{12}O_3$	BEH; silica; diol	BEH; silica; diol	BEH; silica; diol
**53**	470-82-6	eucalyptol (1,8-cineole)	154.25	-	-4.2	2.80	0	1	$C_{10}H_{18}O$	n/a	n/a	n/a
**54**	163222-33-1	ezetimibe	409.43	9.72	-0.2	3.96	4	2	$C_{24}H_{21}F_2NO_3$	BEH; silica; diol	BEH; silica; diol	BEH; silica; diol
**55**	4602-84-0	farnesol	222.37	14.69	-	4.83	1	1	$C_{15}H_{26}O$	BEH; silica; diol	BEH; silica; diol	BEH; silica; diol
**56**	29679-58-1	fenoprofen	242.27	4.2	-	3.72	3	1	$C_{15}H_{14}O_3$	silica; diol	BEH; silica; diol	silica; diol
**57**	5104-49-4	flurbiprofen	244.26	4.14	-	3.66	2	1	$C_{15}H_{13}FO_2$	BEH; silica; diol	BEH; silica; diol	BEH; silica; diol
**58**	93957-54-1	fluvastatin	411.47	4.27	-	4.57	5	3	$C_{24}H_{26}FNO_4$	silica; diol	silica; diol	silica; diol
**59**	54-28-4	gamma-tocopherol	416.68	11.05	-	10.72	2	1	$C_{28}H_{48}O_2$	silica; diol	silica; diol	silica; diol
**60**	14101-61-2	gamma-tocotrienol	410.63	11.05	-	9.46	2	1	$C_{28}H_{42}O_2$	silica; diol	silica; diol	silica; diol
**61**	520-33-2	hesperetin	302.28	7.49	-	1.94	6	3	$C_{16}H_{16}O_6$	BEH; silica; diol	BEH; silica; diol	BEH; silica; diol
**62**	520-26-3	hesperidin	610.56	7.15	-	-1.21	15	8	$C_{28}H_{34}O_{15}$	silica; diol	silica; diol	silica; diol
**63**	4270-27-3	chlorouracil (4-Chloro-2,6-dihydroxypyrimidine)	146.53	6.24	-	0.03	4	2	$C_4H_3ClN_2O_2$	BEH; silica; diol	BEH; silica; diol	BEH; silica; diol
**64**	15687-27-1	ibuprofen	206.28	4.41	-	3.50	2	1	$C_{13}H_{18}O_2$	silica; diol	BEH; silica; diol	silica; diol
**65**	50-49-7	imipramine	280.41	-	9.49	4.36	2	0	$C_{19}H_{24}N_2$	silica; diol	BEH; silica; diol	BEH; silica; diol

n.	CAS No.	analyte	Mw	pKa acid	pKa basic	log P	H acc.	H donor	molecular formula	MeOH	MeOH + 10 mM NH_4	MeOH + 2% H_2O
**66**	1516864-05-3	atorvastatin impurity A	540.65	4.29	0.39	3.91	7	4	$C_{33}H_{36}N_2O_5$	silica; diol	silica; diol	silica; diol
**67**	842103-12-2	atorvastatin impurity B	558.64	4.29	0.38	3.85	7	4	$C_{33}H_{35}FN_2O_5$	BEH; silica; diol	silica; diol	BEH; silica; diol
**68**	693793-53-2	atorvastatin impurity C	576.63	4.29	0.37	3.78	7	4	$C_{33}H_{34}F_2N_2O_5$	silica; diol	silica; diol	silica; diol
**69**	53-86-1	indomethacin	357.79	3.96	-	4.25	5	1	$C_{19}H_{16}ClNO_4$	BEH; diol	BEH; silica; diol	BEH; silica; diol
**70**	480-19-3	isorhamnetine	316.26	6.31	-	2.79	4	11	$C_{16}H_{12}O_7$	n/a	diol	n/a
**71**	22071-15-4	ketoprofen	254.28	4.23	-	2.91	3	1	$C_{16}H_{14}O_3$	BEH; silica; diol	BEH; silica; diol	BEH; silica; diol
**72**	36894-69-6	labetalol	328.41	8.21	9.3	2.72	5	5	$C_{19}H_{24}N_2O_3$	silica; diol	BEH; silica; diol	silica; diol
**73**	125995-03-1	atorvastatin lactone	540.62	13.39	0.38	3.90	6	2	$C_{33}H_{33}FN_2O_4$	BEH; silica; diol	BEH; silica; diol	BEH; silica; diol
**74**	1256388-51-8	ledipasvir	889	11.2	5.42	4.54	14	4	$C_{49}H_{54}F_2N_8O_6$	BEH; silica; diol	BEH; silica; diol	BEH; silica; diol
**75**	75330-75-5	lovastatin	404.54	13.49	-	4.31	5	1	$C_{24}H_{36}O_5$	BEH; silica; diol	BEH; silica; diol	BEH; silica; diol
**76**	491-70-3	luteolin	286.24	6.5	-	2.70	6	4	$C_{15}H_{10}O_6$	silica	n/a	silica
**77**	376348-65-1	maraviroc	513.67	14.8	10.24	5.30	6	1	$C_{29}H_{41}F_2N_5O$	silica; diol	BEH; silica; diol	silica; diol
**78**	51384-51-1	metoprolol	267.36	13.89	9.43	1.63	4	2	$C_{15}H_{25}NO_3$	silica; diol	BEH; silica; diol	BEH; silica; diol
**79**	480-41-1	naringenin	272.25	7.52	-	2.63	3	8	$C_{15}H_{12}O_5$	BEH; silica; diol	BEH; silica; diol	BEH; silica; diol
**80**	10236-47-2	naringin	580.54	7.17	-	-0.20	8	22	$C_{27}H_{32}O_{14}$	BEH; silica; diol	BEH; silica; diol	BEH; silica; diol
**81**	99-87-6	p-cymene	134.22	14	-	4.01	0	0	$C_{10}H_{14}$	n/a	n/a	n/a
**82**	60-81-1	phloridzine	436.41	7.15	-	-0.37	7	17	$C_{21}H_{24}O_{10}$	BEH; silica; diol	BEH; silica; diol	BEH; silica; diol
**83**	13523-86-9	pindolol	248.32	13.94	9.54	1.68	4	3	$C_{14}H_{20}N_2O_2$	BEH; silica; diol	BEH; silica; diol	BEH; silica; diol
**84**	147511-69-1	pitavastatin	421.46	4.24	4.68	1.92	3	8	$C_{25}H_{24}FNO_4$	BEH; silica; diol	BEH; silica; diol	silica; diol
**85**	81093-37-0	pravastatin	424.53	4.31	-	2.21	4	11	$C_{23}H_{36}O_7$	BEH; silica; diol	BEH; silica; diol	BEH; silica; diol
**86**	525-66-6	propranolol	259.34	13.84	9.5	2.90	2	5	$C_{16}H_{21}NO_2$	BEH; silica; diol	BEH; silica; diol	BEH; silica; diol
**87**	501-36-0	resveratrol	228.24	9.22	-	3.02	3	3	$C_{14}H_{12}O_3$	BEH; silica; diol	BEH; silica; diol	BEH; silica; diol

n.	CAS No.	analyte	Mw	pKa acid	pKa basic	log P	H acc.	H donor	molecular formula	MeOH	MeOH + 10 mM NH_4	MeOH + 2% H_2O
**88**	155213-67-5	ritonavir	720.94	11.47	2.51	2.33	11	4	$C_{37}H_{48}N_6O_5S_2$	BEH; silica; diol	BEH; silica; diol	BEH; silica; diol
**89**	123441-03-2	S-rivastigmine	250.34	-	8.62	2.06	0	4	$C_{14}H_{22}N_2O_2$	silica; diol	BEH; silica; diol	silica; diol
**90**	415973-05-6	R-rivastigmine	250.34	-	8.62	2.06	0	4	$C_{14}H_{22}N_2O_2$	silica; diol	BEH; silica; diol	silica; diol
**91**	18559-94-9	salbutamol	239.31	9.99	9.62	0.69	4	8	$C_{13}H_{21}NO_3$	silica; diol	silica; diol	silica; diol
**92**	92-61-5	scopoletin	192.17	7.91	-	1.38	4	1	$C_{10}H_8O_4$	BEH; silica; diol	BEH; silica; diol	BEH; silica; diol
**93**	138-59-0	shikimic acid	174.15	4.48	-	-2.22	5	4	$C_7H_{10}O_5$	BEH; silica; diol	BEH; silica; diol	BEH; silica; diol
**94**	923604-59-5	simeprevir	749.94	4.47	3.01	6.10	12	2	$C_{38}H_{47}N_5O_7S_2$	BEH; silica; diol	BEH; silica; diol	BEH; silica; diol
**95**	79902-63-9	simvastatin	418.57	13.49	-	4.72	5	1	$C_{25}H_{38}O_5$	BEH; silica; diol	BEH; silica; diol	BEH; silica; diol
**96**	486460-32-6	sitagliptin	407.31	-	7.2	2.06	6	2	$C_{16}H_{15}F_6N_5O$	silica	BEH; silica	silica
**97**	1190307-88-0	sofosbuvir	529.45	9.39	-3.26	2.21	12	3	$C_{22}H_{29}FN_3O_9P$	BEH; silica; diol	BEH; silica; diol	BEH; silica; diol
**98**	603-61-2	tamarixetin	316.26	6.31	-	2.67	7	4	$C_{16}H_{12}O_7$	silica	silica; diol	silica; diol
**99**	611-40-5	tectoridin	464.4	6.1	-	0.54	11	6	$C_{22}H_{22}O_{11}$	silica; diol	silica; diol	silica; diol
**100**	548-77-6	tectorigenin	300.26	6.49	-	2.84	3	9	$C_{16}H_{12}O_6$	silica; diol	BEH; silica; diol	silica; diol
**101**	58-22-0	testosterone	288.43	15.06	-	3.18	1	3	$C_{19}H_{28}O_2$	BEH; silica; diol	BEH; silica; diol	BEH; silica; diol
**102**	274693-27-5	ticagrelor	522.57	13.26	3.05	2.02	4	14	$C_{23}H_{28}F_2N_6O_4S$	BEH; silica; diol	BEH; silica; diol	BEH; silica; diol
**103**	140-10-3	trans-cinnamic acid	148.16	4.34	-	1.21	2	1	$C_9H_8O_2$	BEH; silica; diol	BEH; silica; diol	BEH; silica; diol
**104**	501-94-0	tyrosol	138.16	10.17	-	0.85	2	2	$C_8H_{10}O_2$	BEH; silica; diol	BEH; silica; diol	BEH; silica; diol
**105**	66-22-8	uracil	112.09	8.95	-4.19	-1.04	4	2	$C_4H_4N_2O_2$	silica; diol	BEH; silica; diol	BEH; silica; diol
**106**	121-33-5	vanillin	152.15	7.78	-	1.21	3	1	$C_8H_8O_3$	BEH; silica; diol	BEH; silica; diol	BEH; silica; diol
**107**	224785-90-4	vardenafil	488.6	9.86	7.15	3.64	10	1	$C_{23}H_{32}N_6O_4S$	BEH; silica; diol	BEH; silica; diol	BEH; silica; diol

Table S2: Molecular descriptors used in the study, calculated with CDK Descriptor Calculator (v.1.4.8).

type and class of molecular descriptors	individual descriptors (abbreviation)	meaning
**ALOGP**	ALogP	Ghose-Crippen LogKow (octanol-water coefficient)
Constitutional Descriptor	AlogP2	Ghose-Crippen octanol water coefficient squared
(atom additive logP and molar refractivity values; described by Ghose and Crippen)	AMR	Ghose-Crippen molar refractivity
**APol** ***Electronic Descriptor***	Apol	sum of the atomic polarizabilities (including implicit hydrogens)
**AcidicGroupContent** ***Constitutional Descriptor***	nAcid	number of acidic groups
**BCUT**	BCUTw-1l	nhigh (number of highest eigenvalue) lowest atom weighted BCUTS
Hybrid Descriptor	BCUTw-1h	nlow (number of lowest eigenvalue) highest atom weighted BCUTS
(eigenvalue-based descriptor noted for its utility in chemical diversity described by Pearlman et al; a weighted version of the Burden matrix)	BCUTc-1l	nhigh (number of highest eigenvalue) lowest partial charge weighted BCUTS
	BCUTc-1h	nlow (number of lowest eigenvalue) highest partial charge weighted BCUTS
	BCUTp-1l	nhigh (number of highest eigenvalue) lowest polarizability weighted BCUTS
	BCUTp-1h	nlow (number of lowest eigenvalue) highest polarizability weighted BCUTS
**CPSA**	PPSA-1	partial positive surface area; sum of surface area on positive parts of molecule
Electronic and Geometrical Descriptor	PPSA-2	partial positive surface area * total positive charge on the molecule
(29 Charged Partial Surface Area Descriptors)	PPSA-3	charge weighted partial positive surface area
	PNSA-1	partial negative surface area; sum of surface area on negative parts of molecule
	PNSA-2	partial negative surface area * total negative charge on the molecule
	PNSA-3	charge weighted partial negative surface area
	DPSA-1	difference of PPSA-1 and PNSA-1
	DPSA-2	difference of FPSA-2 and PNSA-2
	DPSA-3	difference of PPSA-3 and PNSA-3
	FPSA-1	PPSA-1 / total molecular surface area
	FPSA-2	PPSA-2 / total molecular surface area
	FPSA-3	PPSA-3 / total molecular surface area
	FNSA-1	PNSA-1 / total molecular surface area
	FNSA-2	PNSA-2 / total molecular surface area
	FNSA-3	PNSA-3 / total molecular surface area
	WPSA-1	PPSA-1 * total molecular surface area / 1000
	WPSA-2	PPSA-2 * total molecular surface area / 1000
	WPSA-3	PPSA-3 * total molecular surface area / 1000
	WNSA-1	PNSA-1 * total molecular surface area / 1000
	WNSA-2	PNSA-2 * total molecular surface area / 1000
	WNSA-3	PNSA-3 * total molecular surface area / 1000
	RPCG	relative positive charge; most positive charge / total positive charge
	RNCG	relative negative charge; most negative charge / total negative charge

type and class of molecular descriptors	individual descriptors (abbreviation)	meaning
	RPCS	relative positive charge surface area; most positive surface area * RPCG
	RNCS	relative negative charge surface area; most negative surface area * RNCG
	THSA	sum of solvent accessible surface areas of atoms with absolute value of partial charges less than 0.2
	TPSA	sum of solvent accessible surface areas of atoms with absolute value of partial charges less than 0.2
	RHSA	THSA / total molecular surface area
	RPSA	TPSA / total molecular surface area
**WHIM** *Hybrid Descriptor* ***(Weighted Holistic Invariant Molecular descriptors; based on a number of atom weightings)***	Wlambda1.unity;	directional descriptor; related to molecular size
	Wlambda2.unity	directional descriptor; related to molecular size
	Wlambda3.unity	directional descriptor; related to molecular size
	Wnu1.unity	directional descriptor; related to molecular shape
	Wnu2.unity	directional descriptor; related to molecular shape
	Weta1.unity	directional descriptor; related to density of the atoms distribution
	Weta2.unity	directional descriptor; related to density of the atoms distribution
	Weta3.unity	directional descriptor; related to density of the atoms distribution
	WT.unity	non-directional descriptor; related to linear contributions to the total molecular dimension
	WA.unity	non-directional descriptor; related to quadratic contributions to the total molecular dimension
	WV.unity	non-directional descriptor; contains also the third-order term;
	WK.unity	non-directional descriptor; molecular shape
	WD.unity	non-directional descriptor; the total molecular density
**MDE** *Topological Descriptor* (***Molecular Distance Edge Descriptors for C, O, and N***)	MDEC-11	molecular distance edge between all primary carbons
	MDEC-12	molecular distance edge between all primary and secondary carbons
	MDEC-13	molecular distance edge between all primary and tertiary carbons
	MDEC-14	molecular distance edge between all primary and quaternary carbons
	MDEC-22	molecular distance edge between all secondary carbons
	MDEC-23	molecular distance edge between all secondary and tertiary carbons
	MDEC-24	molecular distance edge between all secondary and quaternary carbons
	MDEC-33	molecular distance edge between all tertiary carbons
	MDEC-34	molecular distance edge between all tertiary and quaternary carbons
	MDEC-44	molecular distance edge between all quaternary carbons
	MDEO-11	molecular distance edge between all primary oxygens
	MDEO-12	molecular distance edge between all primary and secondary oxygens
	MDEO-22	molecular distance edge between all secondary oxygens
	MDEN-12	molecular distance edge between all primary and secondary nitrogens
	MDEN-13	molecular distance edge between all primary and tertiary nitrogens
	MDEN-22	molecular distance edge between all secondary nitrogens
	MDEN-23	molecular distance edge between all secondary and tertiary nitrogens
	MDEN-33	molecular distance edge between all tertiary nitrogens
**AromaticAtomsCount**	naAromAtom	number of aromatic atoms in an atom container

type and class of molecular descriptors	individual descriptors (abbreviation)	meaning
Constitutional Descriptor		
**AromaticBonsCount** ***Constitutional Descriptor***	nAromBond	number of aromatic atoms in an AtomContainer; based on the number of aromatic bounds
**AtomCount** ***Constitutional Descriptor***	nAtom	number of atoms of a certain element type
**AutocorrelationCharge** ***Topological Descriptor*** ***(the Moreau-Broto autocorrelation descriptors using partial charges)***	ATSc1	ATS autocorrelation descriptor, weighted by charges
	ATSc2	ATS autocorrelation descriptor, weighted by charges
	ATSc3	ATS autocorrelation descriptor, weighted by charges
	ATSc4	ATS autocorrelation descriptor, weighted by charges
	ATSc5	ATS autocorrelation descriptor, weighted by charges
**AutocorrelationMass** ***Topological Descriptor*** ***(the Moreau-Broto autocorrelation descriptors using atomic weight)***	ATSm1	ATS autocorrelation descriptor, weighted by scaled atomic mass
	ATSm2	ATS autocorrelation descriptor, weighted by scaled atomic mass
	ATSm3	ATS autocorrelation descriptor, weighted by scaled atomic mass
	ATSm4	ATS autocorrelation descriptor, weighted by scaled atomic mass
	ATSm5	ATS autocorrelation descriptor, weighted by scaled atomic mass
**AutocorrelationPolarizability** ***Topological Descriptor*** ***(the Moreau-Broto autocorrelation descriptors using polarizability)***	ATSp1	ATS autocorrelation descriptor, weighted by polarizability
	ATSp2	ATS autocorrelation descriptor, weighted by polarizability
	ATSp3	ATS autocorrelation descriptor, weighted by polarizability
	ATSp4	ATS autocorrelation descriptor, weighted by polarizability
	ATSp5	ATS autocorrelation descriptor, weighted by polarizability
**BPol** ***Electronic Descriptor***	bpol	sum of the absolute value of the difference between atomic polarizabilities of all bonded atoms in the molecule (including implicit hydrogens)
**BasicGroupCount** ***Constitutional Descriptor***	nBase	number of basic groups
**BondCount** ***Constitutional Descriptor***	nBx	single value with name nBX where X can be s(single bond), d (double), t (triple), a (aromatic), “”(all)
**CarbonTypes** ***Topological Descriptor*** ***(carbon connectivity in the terms of hybridization)***	C1SP1	triply bound carbon bound to one other carbon
	C1SP2	triply bound carbon bound to two other carbons
	C2SP2	doubly bound carbon bound to two other carbons
	C3SP2	doubly bound carbon bound to three other carbons
	C1SP3	singly bound carbon bound to one other carbon
	C2SP3	singly bound carbon bound to two other carbons
	C3SP3	singly bound carbon bound to three other carbons
	C4SP3	singly bound carbon bound to four other carbons
**ChiChain** ***Topological Descriptor***	SCH-3	simple chain, order 3
	SCH-4	simple chain, order 4
	SCH-5	simple chain, order 5
	SCH-6	simple chain, order 6

type and class of molecular descriptors	individual descriptors (abbreviation)	meaning
(evaluates the Kier & Hall Chi chain indices of orders 3,4,5 and 6; type of chain)	SCH-7	simple chain, order 7
	VCH-3	valence chain, order 3
	VCH-4	valence chain, order 4
	VCH-5	valence chain, order 5
	VCH-6	valence chain, order 6
	VCH-7	valence chain, order 7
**ChiCluster** *Topological Descriptor* ***(evaluates the Kier & Hall Chi cluster indices of orders 3,4,5, and 6)***	SC-3	simple cluster, order 3
	SC-4	simple cluster, order 4
	SC-5	simple cluster, order 5
	SC-6	simple cluster, order 6
	VC-3	valence cluster, order 3
	VC-4	valence cluster, order 4
	VC-5	valence cluster, order 5
	VC-6	valence cluster, order 6
**ChiPathCluster** *Topological Descriptor* ***(evaluates the Kier & Hall Chi path cluster indices of orders 4,5, and 6)***	SPC-4	simple path cluster, order 4
	SPC-5	simple path cluster, order 5
	SPC-6	simple path cluster, order 6
	VPC-4	valence path cluster, order 4
	VPC-5	valence path cluster, order 5
	VPC-6	valence path cluster, order 6
**ChiPath** *Topological Descriptor* ***(evaluates the Kier & Hall Chi path indices of orders 0,1,2,3,4,5,6 and 7)***	SP-0	simple path, order 0
	SP-1	simple path, order 1
	SP-2	simple path, order 2
	SP-3	simple path, order 3
	SP-4	simple path, order 4
	SP-5	simple path, order 5
	SP-6	simple path, order 6
	SP-7	simple path, order 7
	VP-0	valence path, order 0
	VP-1	valence path, order 1
	VP-2	valence path, order 2
	VP-3	valence path, order 3
	VP-4	valence path, order 4
	VP-5	valence path, order 5
	VP-6	valence path, order 6
	VP-7	valence path, order 7
**EccentricConnectivityIndex** *Topological Descriptor*	ECCEN	combining distance and adjacency information

type and class of molecular descriptors	individual descriptors (abbreviation)	meaning
**FMF**<br>***Topological Descriptor***	FMF	ratio of heavy atoms in the framework to the total number of heavy atoms in the molecule; characterize the complexity of the molecule
**FragmentComplexity**<br>***Topological Descriptor***	fragC	Complexity of a system; C=abs(B^2-A^2+A)+H/100 where C=complexity, A=number of non-hydrogen atoms, B=number of bonds and H=number of heteroatoms
**GravitationalIndex**<br>***Geometrical Descriptor***<br>***(mass distribution of the molecule)***	GRAV-1	gravitational index of heavy atoms
	GRAV-2	square root of gravitational index of heavy atoms
	GRAV-3	cube root of gravitational index of heavy atoms
	GRAVH-1	gravitational index - hydrogens included
	GRAVH-2	square root of hydrogen-included gravitational index
	GRAVH-3	cube root of hydrogen-included gravitational index
	GRAV-4	grav1 for all pairs of atoms (not just bonded pairs)
	GRAV-5	grav2 for all pairs of atoms (not just bonded pairs)
	GRAV-6	grav3 for all pairs of atoms (not just bonded pairs)
**HBondAcceptorCount**<br>***Electronic Descriptor***	nHBAcc	the number of H bond acceptors using a slightly simplified version of the PHACIR atom types. The groups counted as acceptors: (i) Any O where the formal charge of the oxygen is non-positive (i.e. formal charge ≤ 0) except an aromatic ether O and an O that is adjacent to a N; (ii) Any N where the formal charge of the N is non-positive (i.e. formal charge ≤ 0) except a N that is adjacent to an O
**HBondDonorCount**<br>***Electronic Descriptor***	nHBDon	number of H bond donors using a slightly simplified version of the PHACIR atom types. The groups counted as donors: (i) Any-OH where the formal charge of the O is non-negative (i.e. formal charge ≥ 0); (ii) Any-NH where the formal charge of the N is non-negative (i.e. formal charge ≥ 0)
**HybridizationRatio**<br>***Topologocial Descriptor***	HybRatio	the fraction of sp3 carbons to sp2 carbons; complexity of the molecule
**KierHallSmarts**<br>***Topological Descriptor***<br>***(it counts the number of occurrences of the E-state fragments;***<br>***- single bond; =double bond; # triple bond; : aromatic bond)***	khs.sCH3	count of atom-type E-state: $-CH_3$
	khs.ssCH2	count of atom-type E-state: $-CH_2-$
	khs.dsCH	count of atom-type E-state: =CH-
	khs.aaCH	count of atom-type E-state: :CH:
	khs.sssCH	count of atom-type E-state: >CH-
	khs.tsC	count of atom-type E-state: #C-
	khs.dssC	count of atom-type E-state: =C<
	khs.aasC	count of atom-type E-state: :C:-
	khs.aaaC	count of atom-type E-state: ::C:
	khs.ssssC	count of atom-type E-state: >C<
	khs.sNH2	count of atom-type E-state: $-NH_2$
	khs.ssNH	count of atom-type E-state: $-NH_2-^+$
	khs.aaNH	count of atom-type E-state: :NH:
	khs.tN	count of atom-type E-state: #N
	khs.aaN	count of atom-type E-state: :N:

type and class of molecular descriptors	individual descriptors (abbreviation)	meaning
	khs.sssN	count of atom-type E-state: >N-
	khs.aasN	count of atom-type E-state: :N:-
	khs.sOH	count of atom-type E-state: -OH
	khs.dO	count of atom-type E-state: =O
	khs.ssO	count of atom-type E-state: -O-
	khs.aaO	count of atom-type E-state: :O:
	khs.sF	count of atom-type E-state: -F
	khs.dsssP	count of atom-type E-state: ->P=
	khs.dS	count of atom-type E-state: =S
	khs.ssS	count of atom-type E-state: -S-
	khs.aaS	count of atom-type E-state: :S:
	khs.ddssS	count of atom-type E-state: >S==
	khs.sCl	count of atom-type E-state: -Cl
	khs.sBr	count of atom-type E-state: -Br
**KappaShapeIndices** *Topological Descriptor* *(Kier and Hall kappa molecular shape indices compare the molecular graph with minimal and maximal molecular graphs)*	Kier1	first kappa shape index
	Kier2	second kappa shape index
	Kier3	third kappa shape index
**MomentOfInertia** *Geometrical Descriptor* *(principal moment of inertia, their ratios, and radius of gyration; characterize the mass distribution of a molecule)*	MOMI-X	moment of inertia along X axis
	MOMI-Y	moment of inertia along Y axis
	MOMI-Z	moment of inertia along Z axis
	MOMI-XY	ratio X/Y
	MOMI-XZ	ratio X/Z
	MOMI-YZ	ratio Y/Z
	MOMI-R	radius of the gyration of the molecule
**WeightedPath** *Topological Descriptor* *(The weighted path (molecular ID) descriptors described by Randic. They characterize molecular branching.)*	WTPT-1	molecular ID
	WTPT-2	molecular ID / number of atoms
	WTPT-3	sum of path lengths starting from heteroatoms
	WTPT-4	sum of path lengths starting from oxygens
	WTPT-5	sum of path lengths starting from nitrogens
**WienerNumbers** *Topological Descriptor* *(described by Randic, characterize molecular branching)*	WPATH	weiner path number
	WPOL	weiner polarity number
**LargestChain** *Constitutional Descriptor*	nAtomLC	number of atoms in the largest chain

type and class of molecular descriptors	individual descriptors (abbreviation)	meaning
**LargestPiSystem** ***Constitutional Descriptor***	nAtomP	number of atoms in the largest pi system
**LongestAliphaticChain** ***Constitutional Descriptor***	nAtomLAC	number of atoms in the longest aliphatic chain
**PetitjeanNumber** ***Topological Descriptor***	PetitjeanNuber	the eccentricity of a vertex corresponds to the distance from that vertex to the most remote vertex in the graph. The distance is obtained from the distance matrix as the count of edges between the two vertices. If r(i) is the largest matrix entry in row i of the distance matrix D, then the radius is defined as the smallest of the r(i). The graph diameter D is defined as the largest vertex eccentricity in the graph
**MannholdLogP** ***Constitutional Descriptor***	MLogP	prediction of logP based on the number of carbon and hetero atoms
**PetitjeanShapeIndex** ***Topological/Geometrical Descriptor (anisotropy of molecule)***	topoShape<br>geomShape	topological shape index<br>geometric shape index
**RuleOfFive** ***Constitutional Descriptor***	LipinskiFailures	number failures of the Lipinski's Rule Of 5
**TPSA** ***Topological/Electronic Descriptor***	TopoPSA	topological polar surface area based on fragment contributions
**VABC** ***Constitutional Descriptor***	VABC	values derived from the van der Waals Volume as a Sum of Atomic and Bond Contributions (VABC)
**VAdjMa** ***Topological Descriptor***	VAdjMat	Vertex adjacency information (magnitude): 1 + log2 m where m is the number of heavy-atom bonds
**Weight** ***Constitutional Descriptor***	MW	based on the weight of atoms of a certain element type
**XLogP** ***Constitutional Descriptor***	XLogP	prediction of logP based on the atom-type method
**ZagrebIndex** ***Topological Descriptor***	Zagreb	the sum of the squares of atom degree over all heavy atoms i
**RotatableBondsCount** ***Constitutional Descriptor***	nRotB	number of rotatable bonds is given by the SMARTS specified by Daylight
Other Constitutional Descriptors	tpsaEfficiency<br>nSmallRings<br>nAromRings<br>nRingBlocks<br>nAromBlocks<br>nRings3<br>nRings4<br>nRings5	Polar surface area expressed as a ratio to molecular size<br>Number of small rings from size 3 to 9<br>Number of aromatic rings<br>Total number of distinct ring blocks<br>Total number of "aromatically connected components"<br>individual breakdown of small ring, size 3<br>individual breakdown of small ring, size 4<br>individual breakdown of small ring, size 5

**type and class of molecular descriptors**	**individual descriptors (abbreviation)**	**meaning**
	nRings6	individual breakdown of small ring, size 6
	nRings7	individual breakdown of small ring, size 7

Table S3: Molecular descriptors used in the study grouped by primary category. For the description of molecular descriptors see SI Table S2.

category	molecular descriptor
lipophility	ALogP
	AlogP2
	MLogP
	XLogP
spectral	AMR
polarizability	Apol
	BCUTp-1l
	BCUTp-1h
	ATSp1
	ATSp2
	ATSp3
	ATSp4
	ATSp5
	bpol
physicochemical properties	nAcid
	nBase
	nHBAcc
	nHBDon
	LipinskiFailures
	VABC
size/mass	MW
	BCUTw-1l
	BCUTw-1h
	Wlambda1.unity;
	Wlambda2.unity
	Wlambda3.unity
	ATSm1
	ATSm2
	ATSm3
	ATSm4
	ATSm5
	GRAV-1
	GRAV-2
	GRAV-3
	GRAVH-1
	GRAVH-2
	GRAVH-3
	GRAV-4
	GRAV-5
	GRAV-6
charge/surface area	BCUTc-1l
	BCUTc-1h
	PPSA-1
	PPSA-2
	PPSA-3
	PNSA-1
	PNSA-2
	PNSA-3
	DPSA-1
	DPSA-2
	DPSA-3
	FPSA-1
	FPSA-2
	FPSA-3

	FPNA-1<br>FPNA-2<br>FPNA-3<br>WPSA-1<br>WPSA-2<br>WPSA-3<br>WPNA-1<br>WPNA-2<br>WPNA-3<br>RPCG<br>RNCG<br>RPCS<br>RNCS<br>THSA<br>TPSA<br>RHSA<br>RPSA<br>ATSc1<br>ATSc2<br>ATSc3<br>ATSc4<br>ATSc5<br>TopoPSA<br>tpsaEfficiency
shape	Wnu1.unity<br>Wnu2.unity<br>WT.unity<br>WA.unity<br>WV.unity<br>WK.unity<br>Kier1<br>Kier2<br>Kier3<br>MOMI-X<br>MOMI-Y<br>MOMI-Z<br>MOMI-XY<br>MOMI-XZ<br>MOMI-YZ<br>MOMI-R<br>FMF<br>PetitjeanNuber<br>topoShape<br>geomShape<br>Zagreb
atom distribution/connectivity	Weta1.unity<br>Weta2.unity<br>Weta3.unity<br>WD.unity<br>ECCEN<br>VAdjMat<br>nBx<br>nRotB<br>khs.sF<br>khs.dsssP<br>khs.dS

	khs.ssS
	khs.aaS
	khs.ddssS
	khs.sCl
	khs.sBr
	nAtomLC
	nAtomP
	nAtomLAC
atom distribution/connectivity - carbon	MDEC-11
	MDEC-12
	MDEC-13
	MDEC-14
	MDEC-22
	MDEC-23
	MDEC-24
	MDEC-33
	MDEC-34
	MDEC-44
	C1SP1
	C1SP2
	C2SP2
	C3SP2
	C1SP3
	C2SP3
	C3SP3
	C4SP3
	fragC
	HybRatio
	khs.sCH3
	khs.ssCH2
	khs.dsCH
	khs.ssssC
	khs.sssCH
	khs.tsC
	khs.dssC
atom distribution/connectivity - oxygen	MDEO-11
	MDEO-12
	MDEO-22
	khs.sOH
	khs.dO
	khs.ssO
atom distribution/connectivity - nitrogen	MDEN-12
	MDEN-13
	MDEN-22
	MDEN-23
	MDEN-33
	khs.sNH2
	khs.ssNH
	khs.tN
	khs.sssN
atom distribution/connectivity - aromatic	naAromAtom
	nAromBond
	nAtom
	nSmallRings
	nAromRings
	nRingBlocks

	nAromBlocks
	nRings3
	nRings4
	nRings5
	nRings6
	nRings7
	khs.aaCH
	khs.aasC
	khs.aaaC
	khs.aaO
	khs.aaNH
	khs.aaN
	khs.aasN
molecular branching	WTPT-1
	WTPT-2
	WTPT-3
	WTPT-4
	WTPT-5
	WPATH
	WPOL
topological descriptors of valence electrons, Chi chains, Chi paths	SCH-3
	SCH-4
	SCH-5
	SCH-6
	SCH-7
	VCH-3
	VCH-4
	VCH-5
	VCH-6
	VCH-7
	SC-3
	SC-4
	SC-5
	SC-6
	VC-3
	VC-4
	VC-5
	VC-6
	SPC-4
	SPC-5
	SPC-6
	VPC-4
	VPC-5
	VPC-6
	SP-0
	SP-1
	SP-2
	SP-3
	SP-4
	SP-5
	SP-6
	SP-7
	VP-0
	VP-1
	VP-2
	VP-3

	VP-4<br>VP-5<br>VP-6<br>VP-7

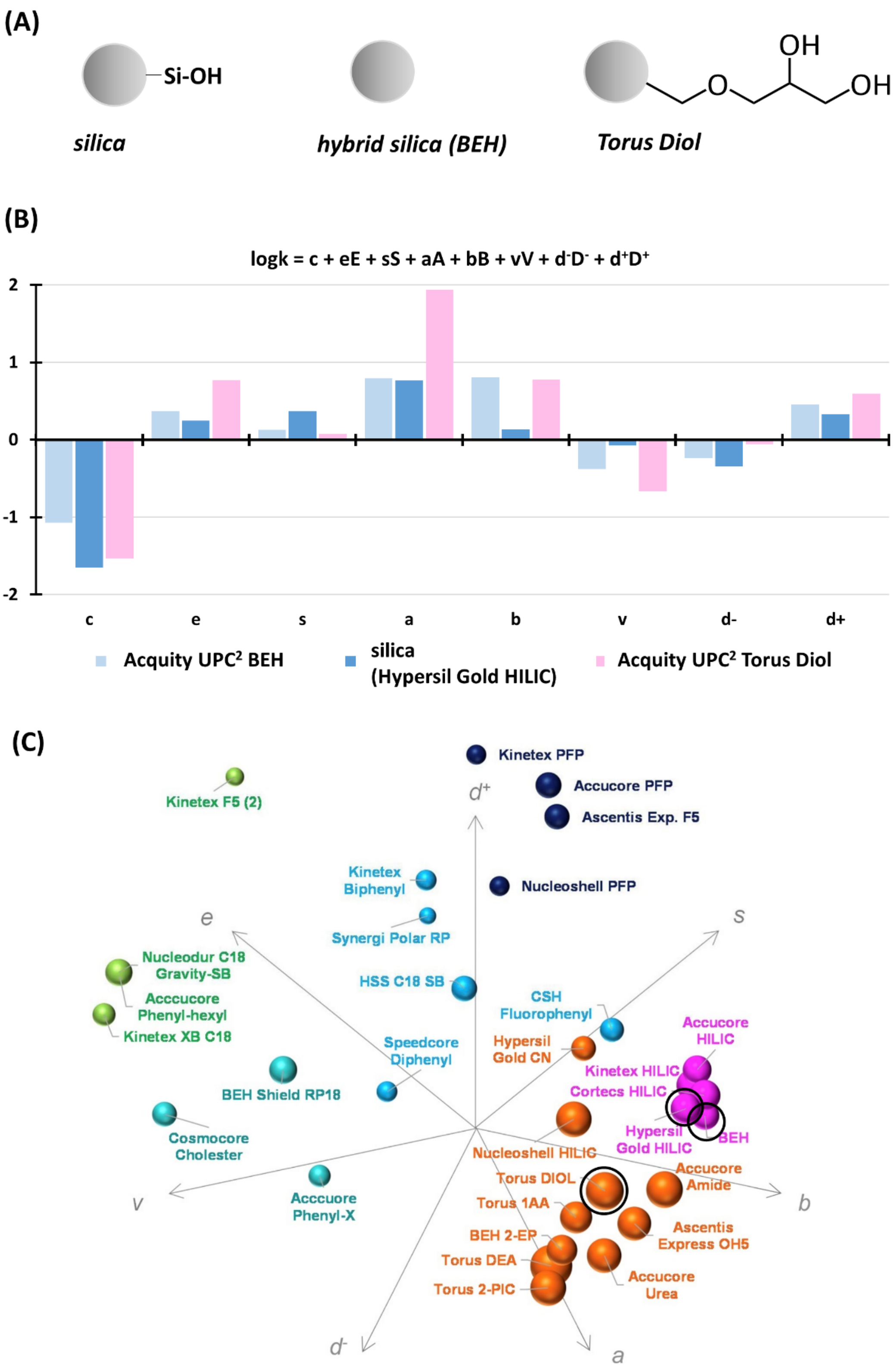

Figure S1. (A) The chemical structure of tested -OH stationary phases, (B) the effect of LSER parameters on silica, BEH, and diol columns, and (C) a spider diagram characterizing stationary phases based on LSER characteristics. Black circles mark columns used for comparison in

(B). The values of LSER parameters were taken from the article published by West et al.[1] The spider diagram was reprinted with permission form ref. 2.[2]

S2. Results

Table S4. Molecular descriptors with the highest effect on the retention using narrowed (52 compounds) and extended sets (57/94/98 compounds) of compounds identified from the weights assigned by the ANNs after 500 training cycles.

	**BEH 52 analytes**	**BEH - *extended* 57 analytes**	**silica 52 analytes**	**silica - *extended* 94 analytes**	**diol 52 analytes**	**diol - *extended* 98 analytes**
**descriptors decreasing the retention**	BCUTc-1l	*BCUTc-1l*	nHBAcc	*nHBAcc*	HybRatio	*HybRatio*
	MDEO-11	*MDEO-11*	RHSA	*FPSA-3*	BCUTc-1l	*BCUTp-1l*
	XLogP	*XLogP*	khs.ddssS	*khs.ssS*	SC-4	*SC-4*
	khs.dO	*khs.ssO*	MDEO-11	*MDEC-12*	RPCG	*VC-6*
	BCUTc-1h	*BCUTp-1l*	RPCG	*BCUTp-1l*	BCUTp-1l	*VC-5*
	RPCG	*nHBAcc*	BCUTc-1l	*VC-6*	BCUTc-1h	*VP-6*
	RHSA	*FNSA-1*	BCUTc-1h	*VC-5*	LipinskiFailures	*XLogP*
	Khs.ddssS	*VC-4*	Weta2.unity	*VPC-6*	khs.dO	*nHBAcc*
	Weta2.unity	*nAtomLC*	LipinskiFailures	*C1SP3*	topoShape	*nRingBlocks*
	LipinskiFailures	*MDEC-12*	XLogP	*nAromRings*	PetitjeanNumber	*ATSc1*
**descriptors increasing the retention**	MDEO-22	*MDEO-22*	tpsaEfficiency	*tpsaEfficiency*	MDEO-22	*MDEO-22*
	TPSA	*TPSA*	MDEO-22	*MDEO-22*	RNCS	*FNSA-2*
	nHBDon	*nHBDon*	nBase	*nBase*	FMF	*TPSA*
	khs.aaNH	*khs.aaNH*	khs.sNH2	*khs.aaN*	MDEC-13	*MDEC-14*
	geomShape	*geomShape*	MDEN-13	*MDEC-13*	MOMI-XZ	*MDEN-12*
	MDEC-13	*MDEC-23*	MDEC-24	*MDEC-23*	MOMI-YZ	*MDEC-34*
	RNCS	*FNSA-3*	Weta3.unity	*MDEC-14*	khs.aaNH	*BCUTp-1h*
	FMF	*C3SP3*	RNCS	*MDEO-12*	geomShape	*Wlambda1.unity*
	nRings7	*C1SP3*	ATSc3	*SC-6*	C4SP3	*Wlambda2.unity*
	nAcid	*khs.ssNH*	RPSA	*C4SP3*	nAcid	*nBase*

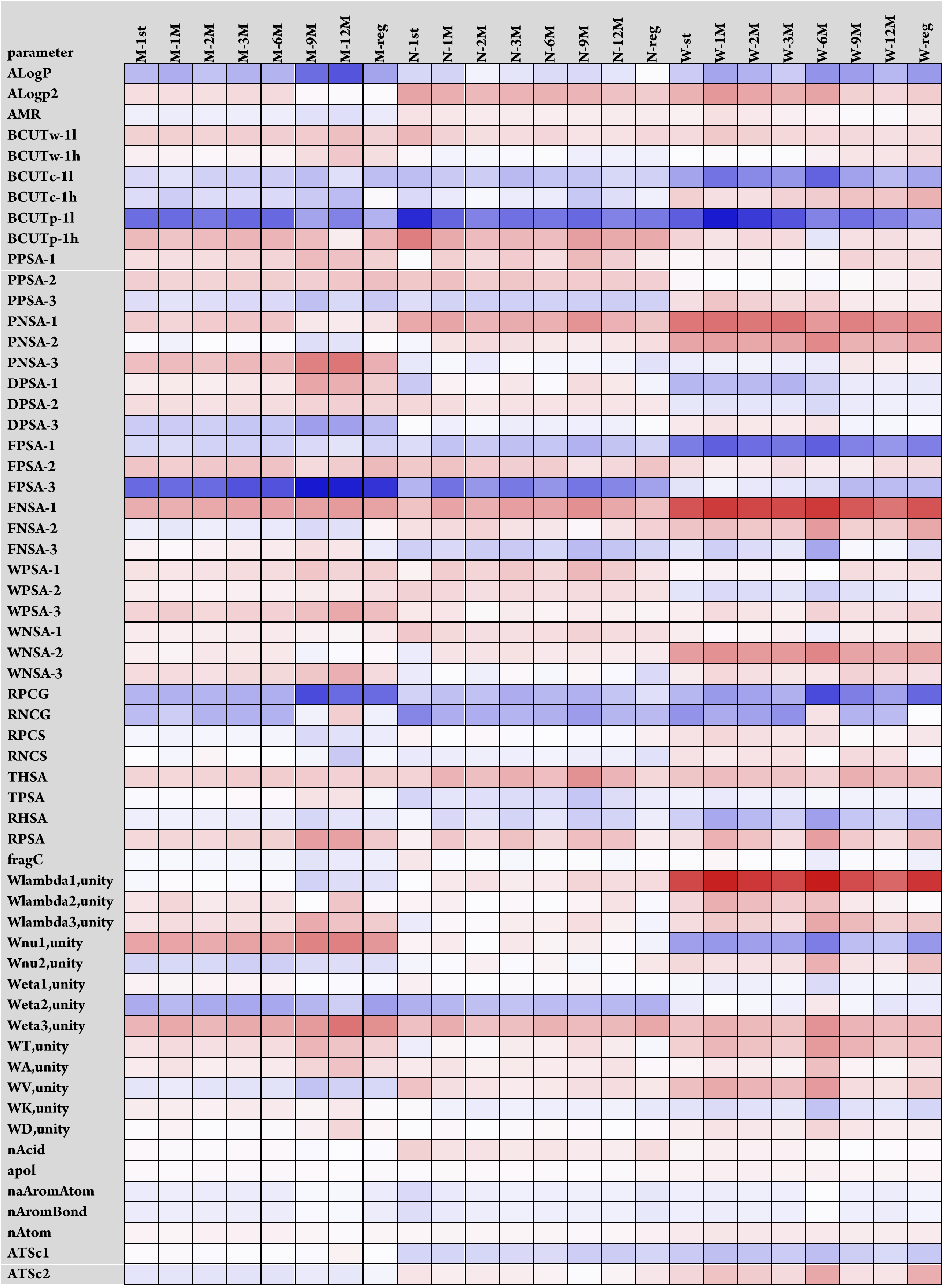
parameter
M-1st
M-1M
M-2M
M-3M
M-6M
M-9M
M-12M
M-reg
N-1st
N-1M
N-2M
N-3M
N-6M
N-9M
N-12M
N-reg
W-st
W-1M
W-2M
W-3M
W-6M
W-9M
W-12M
W-reg
ALogP
ALogp2
AMR
BCUTw-1l
BCUTw-1h
BCUTc-1l
BCUTc-1h
BCUTp-1l
BCUTp-1h
PPSA-1
PPSA-2
PPSA-3
PNSA-1
PNSA-2
PNSA-3
DPSA-1
DPSA-2
DPSA-3
FPSA-1
FPSA-2
FPSA-3
FNSA-1
FNSA-2
FNSA-3
WPSA-1
WPSA-2
WPSA-3
WNSA-1
WNSA-2
WNSA-3
RPCG
RNCG
RPCS
RNCS
THSA
TPSA
RHSA
RPSA
fragC
Wlambda1,unity
Wlambda2,unity
Wlambda3,unity
Wnu1,unity
Wnu2,unity
Weta1,unity
Weta2,unity
Weta3,unity
WT,unity
WA,unity
WV,unity
WK,unity
WD,unity
nAcid
apol
naAromAtom
nAromBond
nAtom
ATSc1
ATSc2

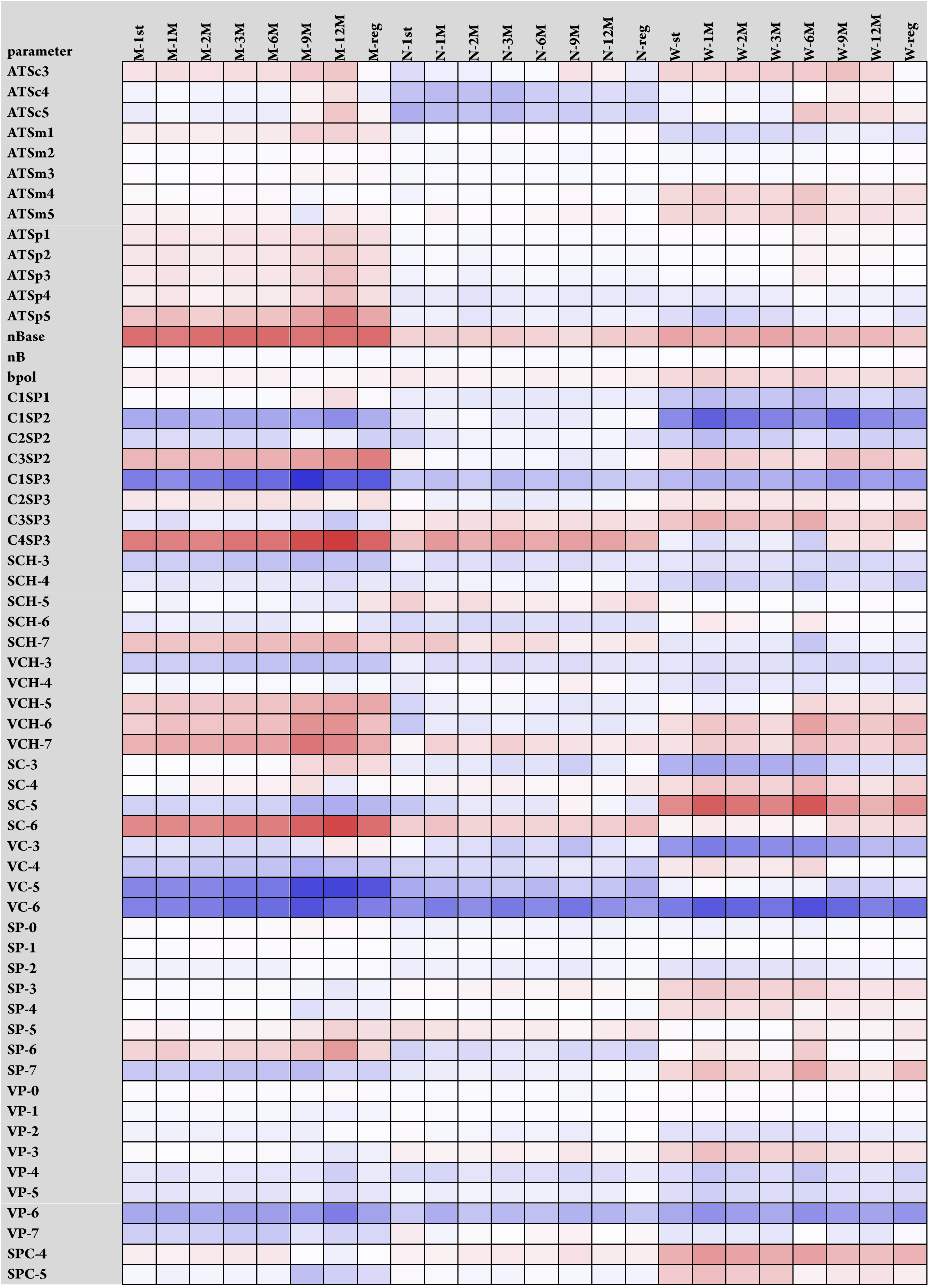

parameter
M-1st
M-1M
M-2M
M-3M
M-6M
M-9M
M-12M
M-reg
N-1st
N-1M
N-2M
N-3M
N-6M
N-9M
N-12M
N-reg
W-st
W-1M
W-2M
W-3M
W-6M
W-9M
W-12M
W-reg
ATSc3
ATSc4
ATSc5
ATSm1
ATSm2
ATSm3
ATSm4
ATSm5
ATSp1
ATSp2
ATSp3
ATSp4
ATSp5
nBase
nB
bpol
C1SP1
C1SP2
C2SP2
C3SP2
C1SP3
C2SP3
C3SP3
C4SP3
SCH-3
SCH-4
SCH-5
SCH-6
SCH-7
VCH-3
VCH-4
VCH-5
VCH-6
VCH-7
SC-3
SC-4
SC-5
SC-6
VC-3
VC-4
VC-5
VC-6
SP-0
SP-1
SP-2
SP-3
SP-4
SP-5
SP-6
SP-7
VP-0
VP-1
VP-2
VP-3
VP-4
VP-5
VP-6
VP-7
SPC-4
SPC-5

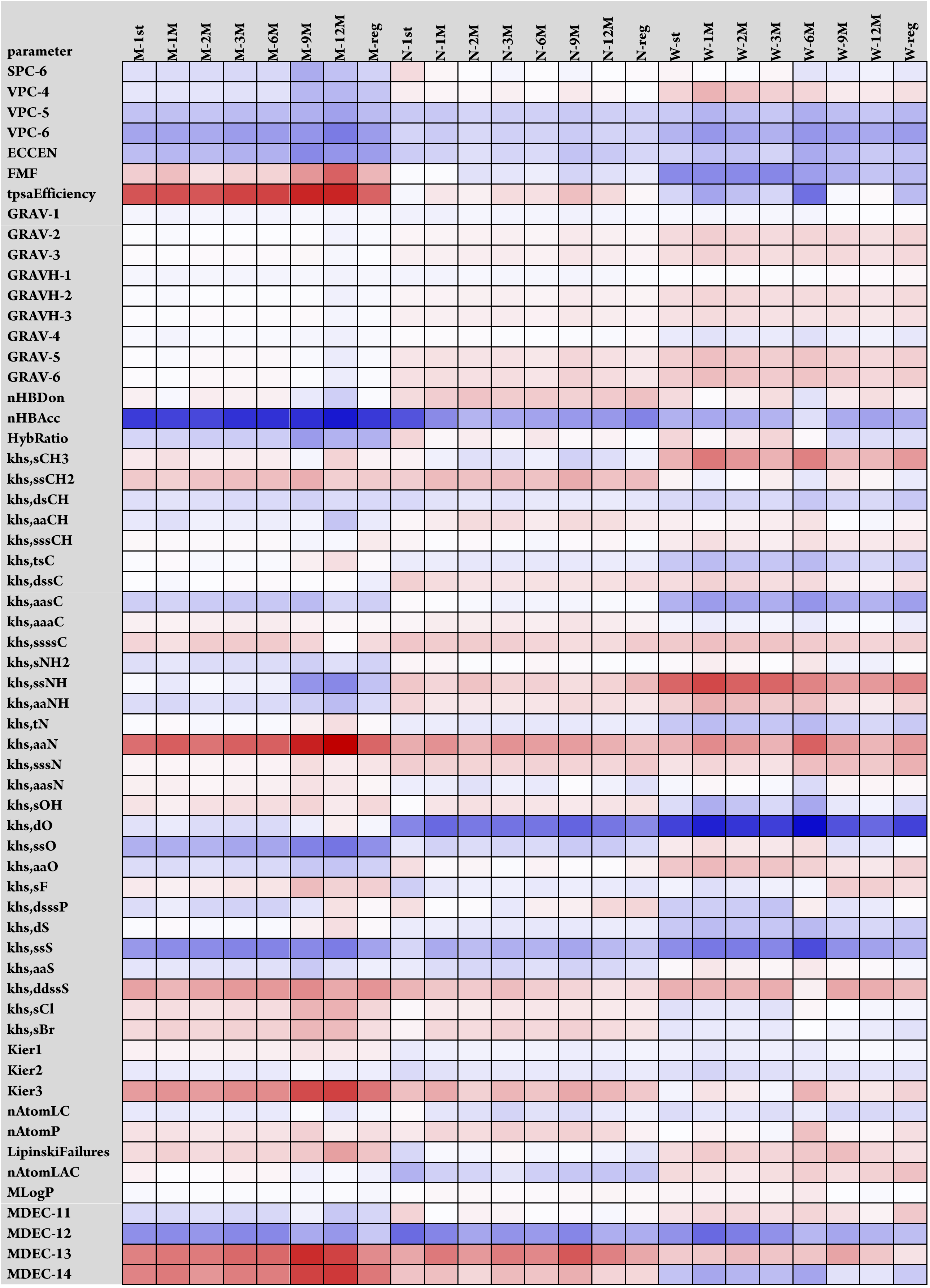
parameter
M-1st
M-1M
M-2M
M-3M
M-6M
M-9M
M-12M
M-reg
N-1st
N-1M
N-2M
N-3M
N-6M
N-9M
N-12M
N-reg
W-st
W-1M
W-2M
W-3M
W-6M
W-9M
W-12M
W-reg
SPC-6
VPC-4
VPC-5
VPC-6
ECCEN
FMF
tpsaEfficiency
GRAV-1
GRAV-2
GRAV-3
GRAVH-1
GRAVH-2
GRAVH-3
GRAV-4
GRAV-5
GRAV-6
nHBDon
nHBAcc
HybRatio
khs,sCH3
khs,ssCH2
khs,dsCH
khs,aaCH
khs,sssCH
khs,tsC
khs,dssC
khs,aasC
khs,aaaC
khs,ssssC
khs,sNH2
khs,ssNH
khs,aaNH
khs,tN
khs,aaN
khs,sssN
khs,aasN
khs,sOH
khs,dO
khs,ssO
khs,aaO
khs,sF
khs,dsssP
khs,dS
khs,ssS
khs,aaS
khs,ddssS
khs,sCl
khs,sBr
Kier1
Kier2
Kier3
nAtomLC
nAtomP
LipinskiFailures
nAtomLAC
MLogP
MDEC-11
MDEC-12
MDEC-13
MDEC-14

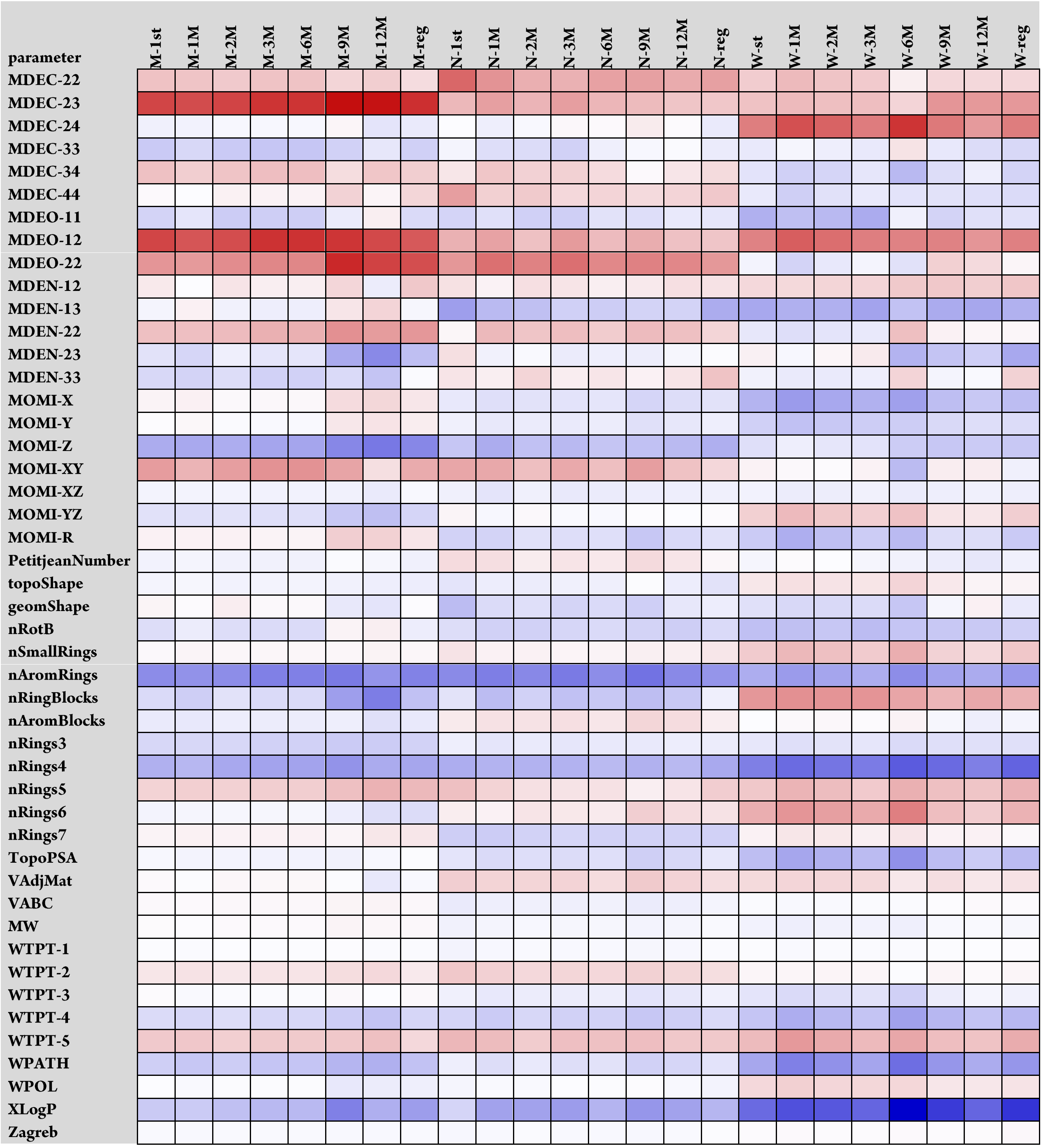

Figure S2. Heatmap of molecular descriptor weights representing their effect on the retention on silica column determined by ANN. Blue – decreasing retention, red – increasing retention. M – MeOH as organic modifier, N – MeOH+10 mmol/L NH_3 as organic modifier, W-MeOH+2% H_2O as organic modifier.

parameter	M-1st	M-1M	M-2M	M-3M	M-6M	M-9M	M-12M	M-reg	N-1st	N-1M	N-2M	N-3M	N-6M	N-9M	N-12M	N-reg	W-1st	W-1M	W-2M	W-3M	W-6M	W-9M	W-12M	W-reg
ALogP																								
ALogp2																								
AMR																								
BCUTw-1l																								
BCUTw-1h																								
BCUTc-1l																								
BCUTc-1h																								
BCUTp-1l																								
BCUTp-1h																								
PPSA-1																								
PPSA-2																								
PPSA-3																								
PNSA-1																								
PNSA-2																								
PNSA-3																								
DPSA-1																								
DPSA-2																								
DPSA-3																								
FPSA-1																								
FPSA-2																								
FPSA-3																								
FNSA-1																								
FNSA-2																								
FNSA-3																								
WPSA-1																								
WPSA-2																								
WPSA-3																								
WNSA-1																								
WNSA-2																								
WNSA-3																								
RPCG																								
RNCG																								
RPCS																								
RNCS																								
THSA																								
TPSA																								
RHSA																								
RPSA																								
fragC																								
Wlambda1,unity																								
Wlambda2,unity																								
Wlambda3,unity																								
Wnu1,unity																								
Wnu2,unity																								
Weta1,unity																								
Weta2,unity																								
Weta3,unity																								
WT,unity																								
WA,unity																								
WV,unity																								
WK,unity																								
WD,unity																								
nAcid																								
apol																								
naAromAtom																								
nAromBond																								
nAtom																								
ATSc1																								
ATSc2																								
ATSc3																								

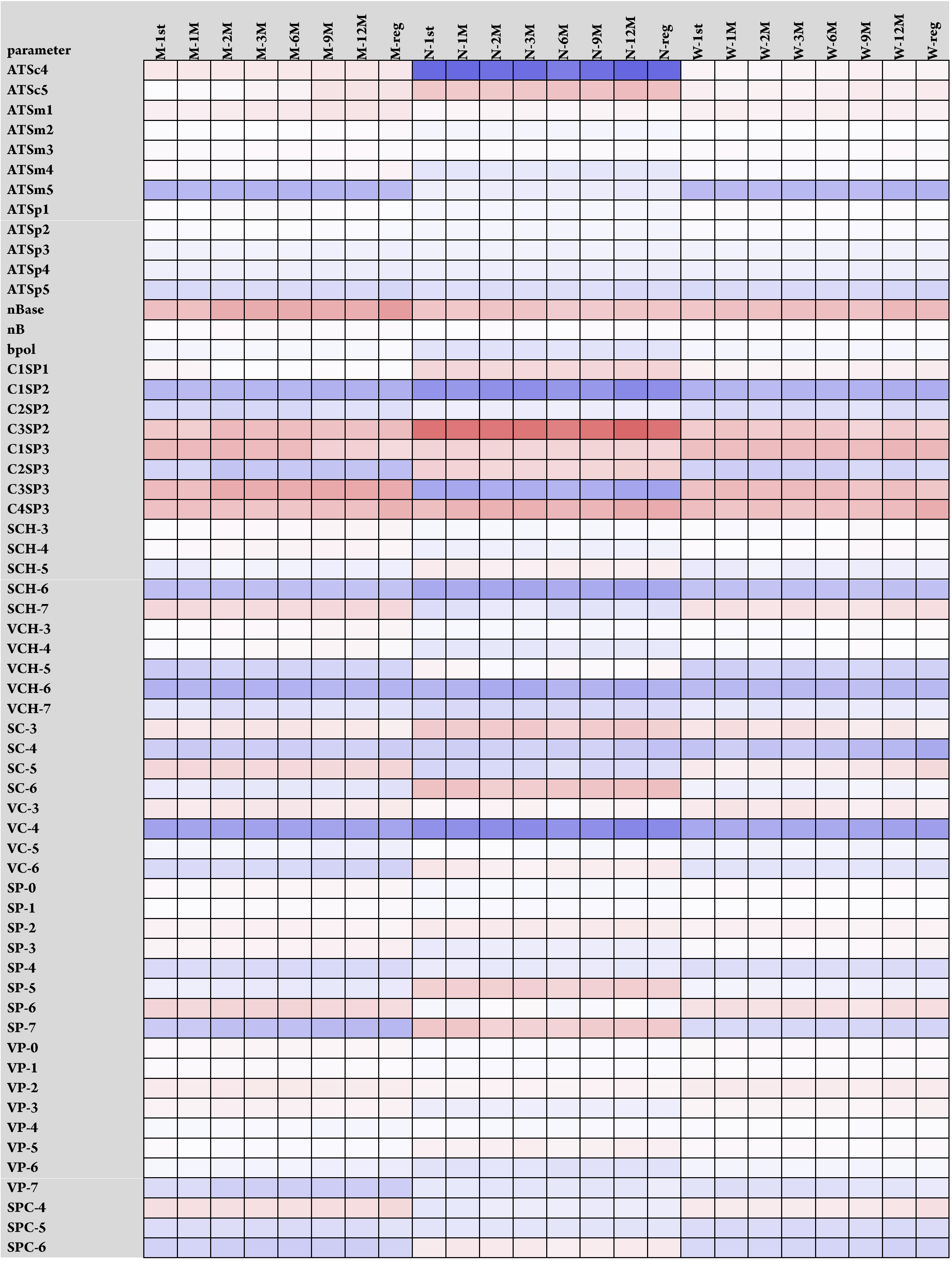
parameter
M-1st
M-1M
M-2M
M-3M
M-6M
M-9M
M-12M
M-reg
N-1st
N-1M
N-2M
N-3M
N-6M
N-9M
N-12M
N-reg
W-1st
W-1M
W-2M
W-3M
W-6M
W-9M
W-12M
W-reg
ATSc4
ATSc5
ATSm1
ATSm2
ATSm3
ATSm4
ATSm5
ATSp1
ATSp2
ATSp3
ATSp4
ATSp5
nBase
nB
bpol
C1SP1
C1SP2
C2SP2
C3SP2
C1SP3
C2SP3
C3SP3
C4SP3
SCH-3
SCH-4
SCH-5
SCH-6
SCH-7
VCH-3
VCH-4
VCH-5
VCH-6
VCH-7
SC-3
SC-4
SC-5
SC-6
VC-3
VC-4
VC-5
VC-6
SP-0
SP-1
SP-2
SP-3
SP-4
SP-5
SP-6
SP-7
VP-0
VP-1
VP-2
VP-3
VP-4
VP-5
VP-6
VP-7
SPC-4
SPC-5
SPC-6

parameter	M-1st	M-1M	M-2M	M-3M	M-6M	M-9M	M-12M	M-reg	N-1st	N-1M	N-2M	N-3M	N-6M	N-9M	N-12M	N-reg	W-1st	W-1M	W-2M	W-3M	W-6M	W-9M	W-12M	W-reg
VPC-4																								
VPC-5																								
VPC-6																								
ECCEN																								
FMF																								
tpsaEfficiency																								
GRAV-1																								
GRAV-2																								
GRAV-3																								
GRAVH-1																								
GRAVH-2																								
GRAVH-3																								
GRAV-4																								
GRAV-5																								
GRAV-6																								
nHBDon																								
nHBAcc																								
HybRatio																								
khs,sCH3																								
khs,ssCH2																								
khs,dsCH																								
khs,aaCH																								
khs,sssCH																								
khs,tsC																								
khs,dssC																								
khs,aasC																								
khs,aaaC																								
khs,ssssC																								
khs,sNH2																								
khs,ssNH																								
khs,aaNH																								
khs,tN																								
khs,aaN																								
khs,sssN																								
khs,aasN																								
khs,sOH																								
khs,dO																								
khs,ssO																								
khs,aaO																								
khs,sF																								
khs,dsssP																								
khs,dS																								
khs,ssS																								
khs,aaS																								
khs,ddssS																								
khs,sCl																								
khs,sBr																								
Kier1																								
Kier2																								
Kier3																								
nAtomLC																								
nAtomP																								
LipinskiFailures																								
nAtomLAC																								
MLogP																								
MDEC-11																								
MDEC-12																								
MDEC-13																								
MDEC-14																								
MDEC-22																								

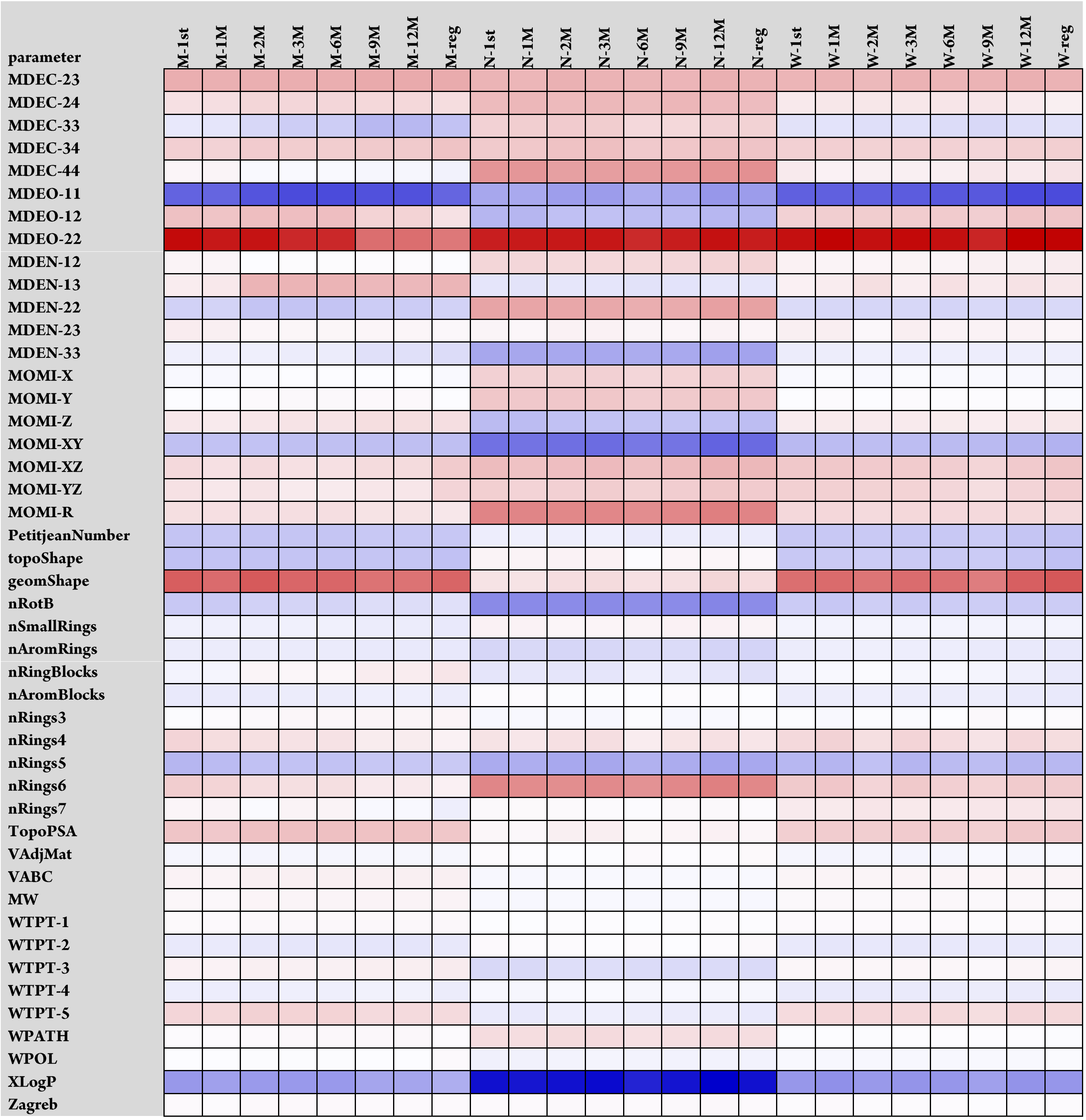

Figure S3. Heatmap of molecular descriptor weights representing their effect on the retention on BEH column determined by ANN. Blue – decreasing retention, red – increasing retention. M – MeOH as organic modifier, N – MeOH+10 mmol/L NH_3 as organic modifier, W-MeOH+2% H_2O as organic modifier.

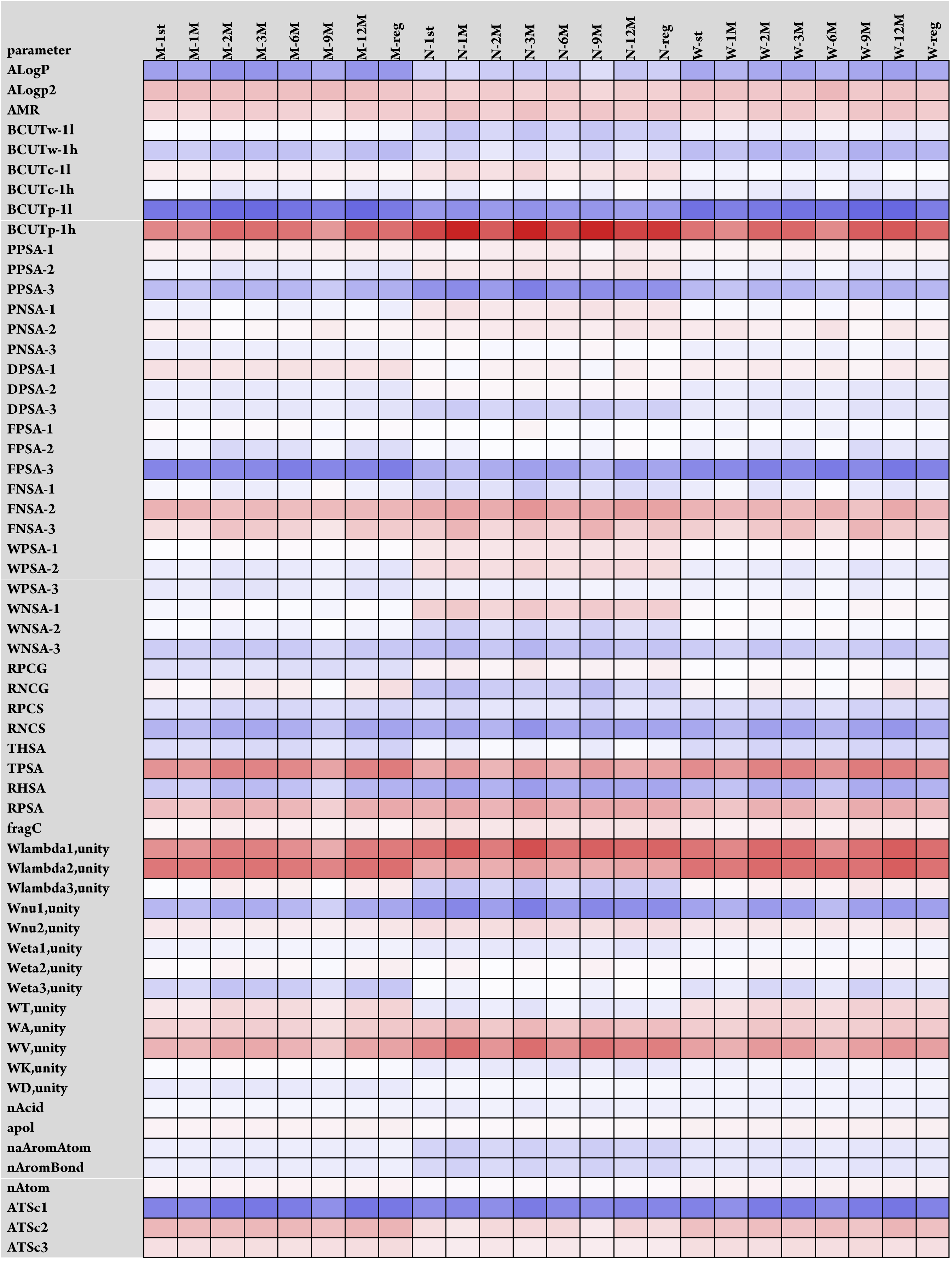
parameter
M-1st
M-1M
M-2M
M-3M
M-6M
M-9M
M-12M
M-reg
N-1st
N-1M
N-2M
N-3M
N-6M
N-9M
N-12M
N-reg
W-st
W-1M
W-2M
W-3M
W-6M
W-9M
W-12M
W-reg
ALogP
ALogp2
AMR
BCUTw-1l
BCUTw-1h
BCUTc-1l
BCUTc-1h
BCUTp-1l
BCUTp-1h
PPSA-1
PPSA-2
PPSA-3
PNSA-1
PNSA-2
PNSA-3
DPSA-1
DPSA-2
DPSA-3
FPSA-1
FPSA-2
FPSA-3
FNSA-1
FNSA-2
FNSA-3
WPSA-1
WPSA-2
WPSA-3
WNSA-1
WNSA-2
WNSA-3
RPCG
RNCG
RPCS
RNCS
THSA
TPSA
RHSA
RPSA
fragC
Wlambda1,unity
Wlambda2,unity
Wlambda3,unity
Wnu1,unity
Wnu2,unity
Weta1,unity
Weta2,unity
Weta3,unity
WT,unity
WA,unity
WV,unity
WK,unity
WD,unity
nAcid
apol
naAromAtom
nAromBond
nAtom
ATSc1
ATSc2
ATSc3

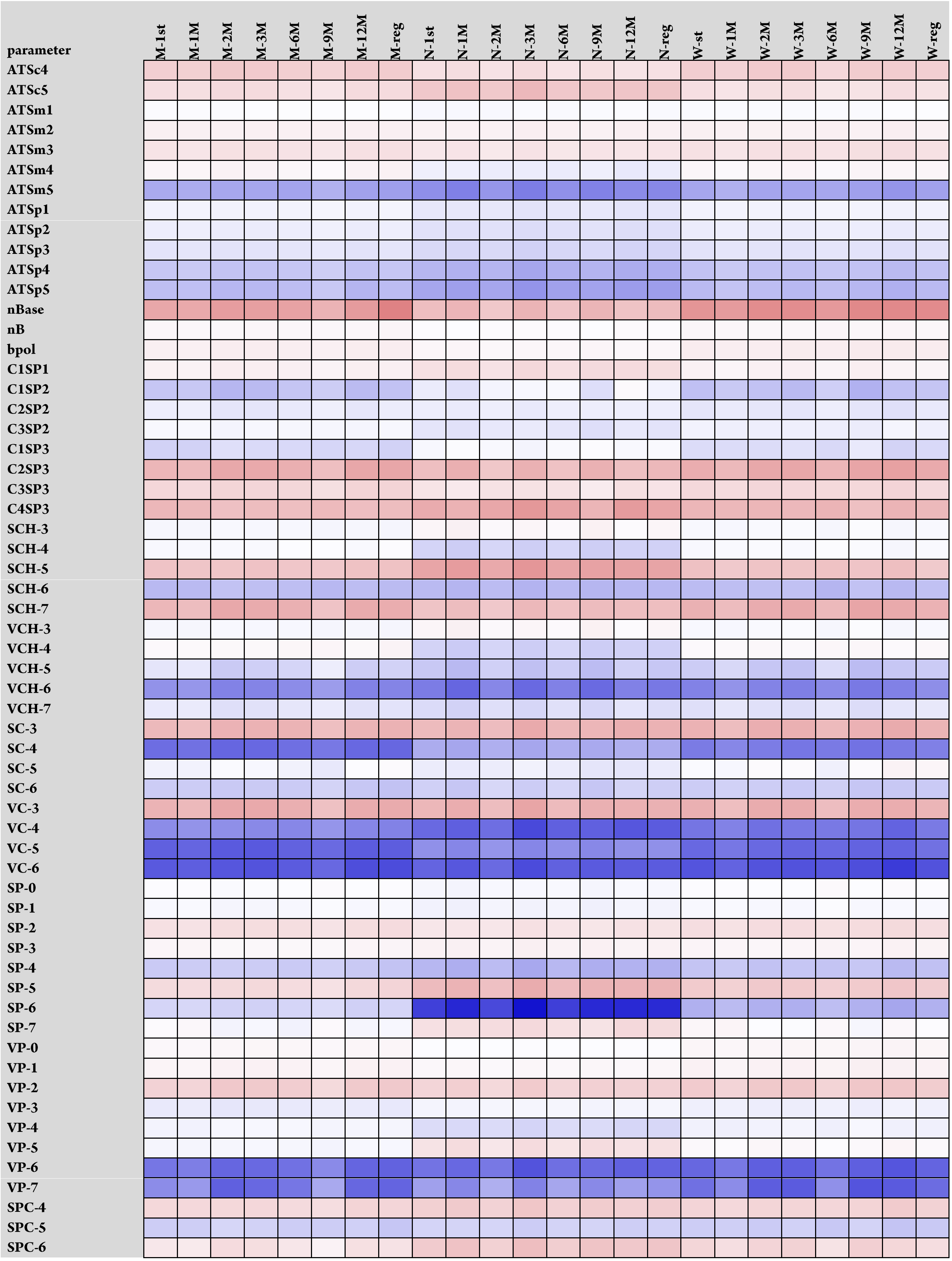
parameter
M-1st
M-1M
M-2M
M-3M
M-6M
M-9M
M-12M
M-reg
N-1st
N-1M
N-2M
N-3M
N-6M
N-9M
N-12M
N-reg
W-st
W-1M
W-2M
W-3M
W-6M
W-9M
W-12M
W-reg
ATSc4
ATSc5
ATSm1
ATSm2
ATSm3
ATSm4
ATSm5
ATSp1
ATSp2
ATSp3
ATSp4
ATSp5
nBase
nB
bpol
C1SP1
C1SP2
C2SP2
C3SP2
C1SP3
C2SP3
C3SP3
C4SP3
SCH-3
SCH-4
SCH-5
SCH-6
SCH-7
VCH-3
VCH-4
VCH-5
VCH-6
VCH-7
SC-3
SC-4
SC-5
SC-6
VC-3
VC-4
VC-5
VC-6
SP-0
SP-1
SP-2
SP-3
SP-4
SP-5
SP-6
SP-7
VP-0
VP-1
VP-2
VP-3
VP-4
VP-5
VP-6
VP-7
SPC-4
SPC-5
SPC-6

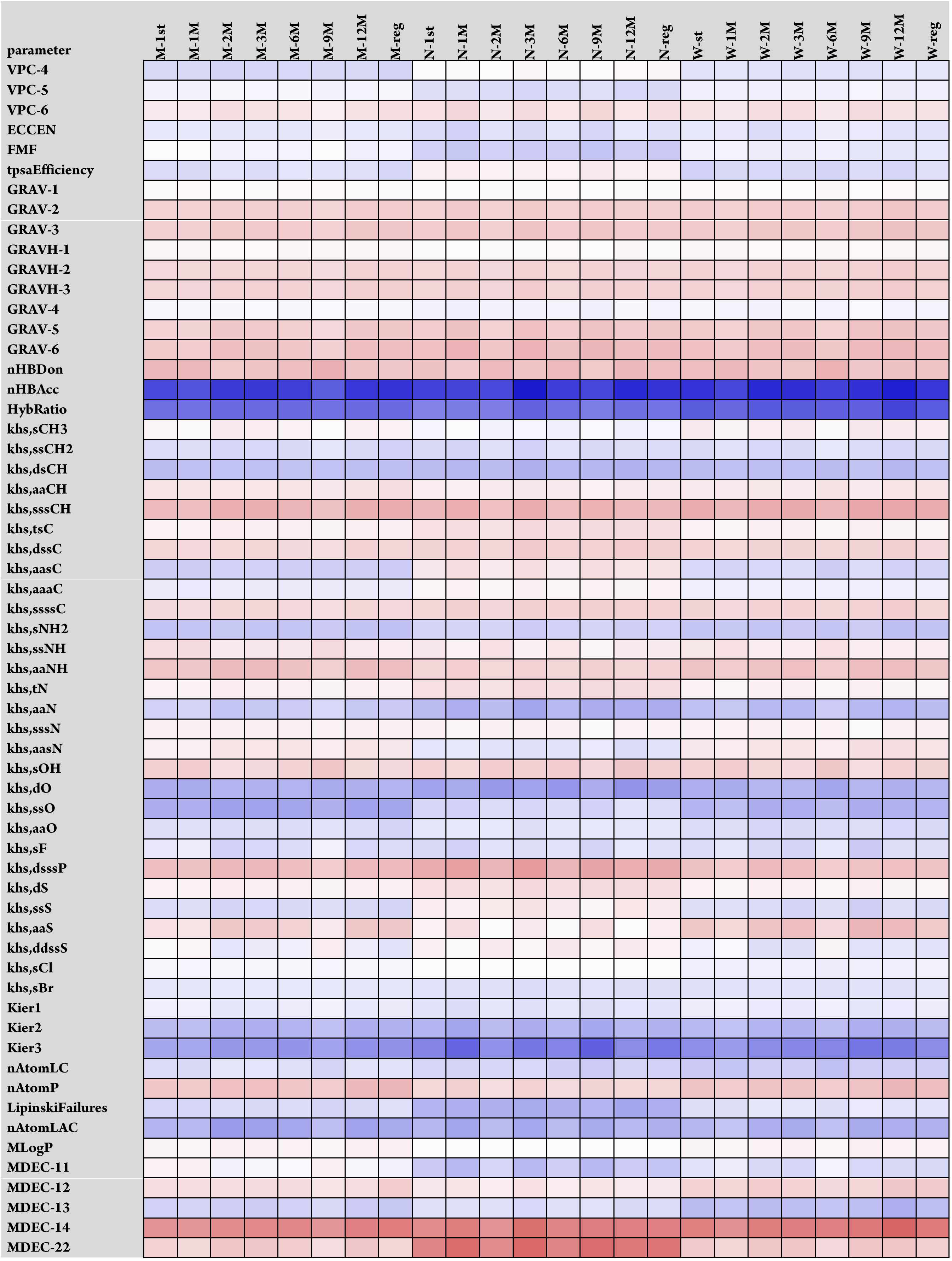
parameter
M-1st
M-1M
M-2M
M-3M
M-6M
M-9M
M-12M
M-reg
N-1st
N-1M
N-2M
N-3M
N-6M
N-9M
N-12M
N-reg
W-st
W-1M
W-2M
W-3M
W-6M
W-9M
W-12M
W-reg
VPC-4
VPC-5
VPC-6
ECCEN
FMF
tpsaEfficiency
GRAV-1
GRAV-2
GRAV-3
GRAVH-1
GRAVH-2
GRAVH-3
GRAV-4
GRAV-5
GRAV-6
nHBDon
nHBAcc
HybRatio
khs,sCH3
khs,ssCH2
khs,dsCH
khs,aaCH
khs,sssCH
khs,tsC
khs,dssC
khs,aasC
khs,aaaC
khs,ssssC
khs,sNH2
khs,ssNH
khs,aaNH
khs,tN
khs,aaN
khs,sssN
khs,aasN
khs,sOH
khs,dO
khs,ssO
khs,aaO
khs,sF
khs,dsssP
khs,dS
khs,ssS
khs,aaS
khs,ddssS
khs,sCl
khs,sBr
Kier1
Kier2
Kier3
nAtomLC
nAtomP
LipinskiFailures
nAtomLAC
MLogP
MDEC-11
MDEC-12
MDEC-13
MDEC-14
MDEC-22

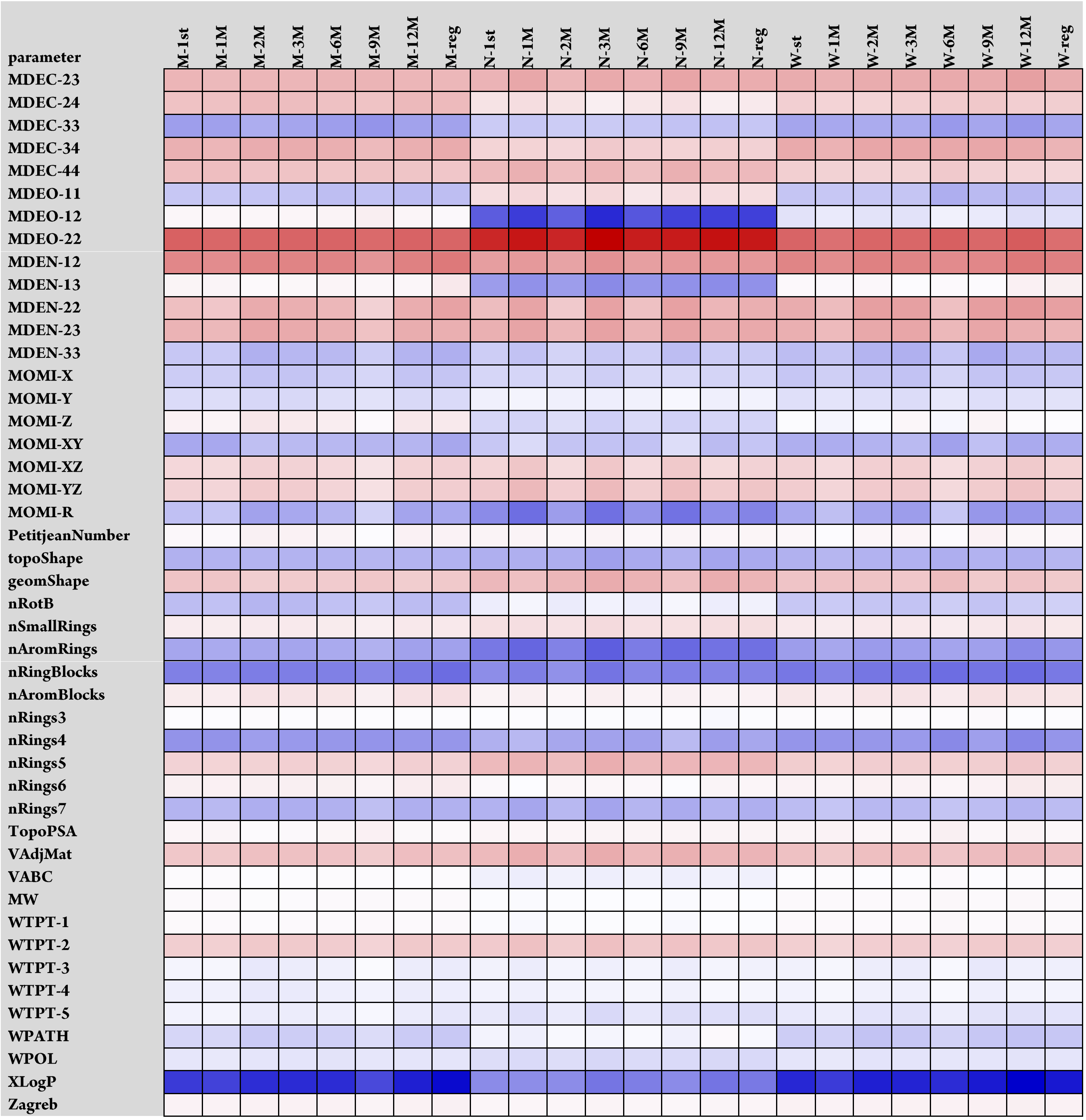

Figure S4. Heatmap of molecular descriptor weights representing their effect on the retention on diol column determined by ANN. Blue – decreasing retention, red – increasing retention. M – MeOH as organic modifier, N – MeOH+10 mmol/L NH_3 as organic modifier, W- MeOH+2% H_2O as organic modifier.

RPCG = the maximal positive charge (blue)/total positive charge (blue+black)

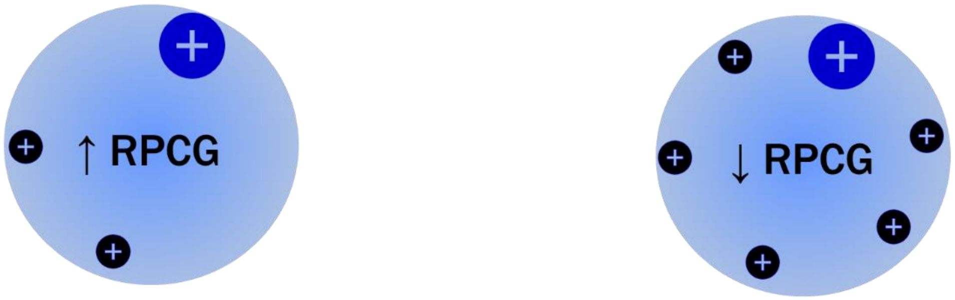

RHSA = sum of solvent accessible surface areas of atoms with absolute value of partial charges less than< 0.2 (red) /total surface area (blue)

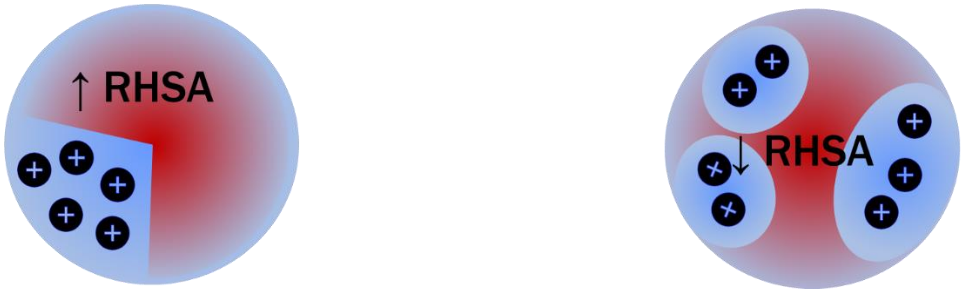

Figure S5. General illustration of molecules with high and low values of RPCG and RHSA.

RNCG = the maximal negative charge (blue)/total negative charge (blue+black)

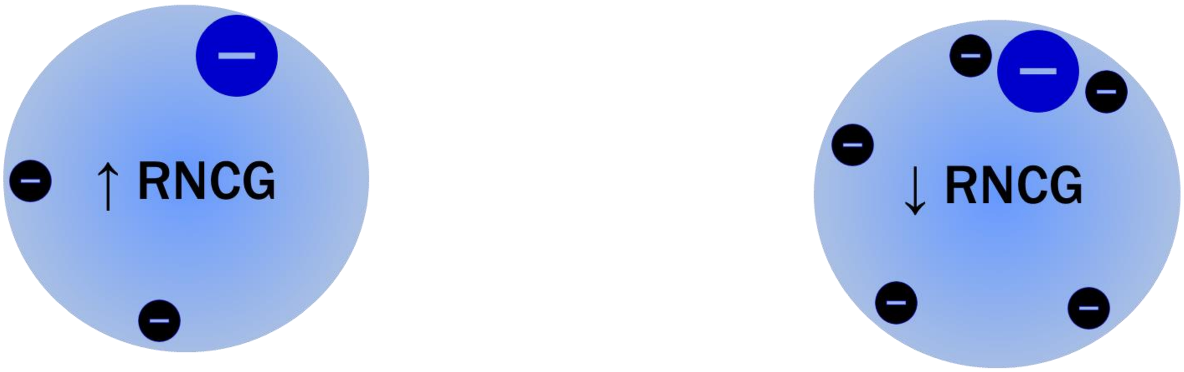

RNCS = most negative surface area (red) * RNCG

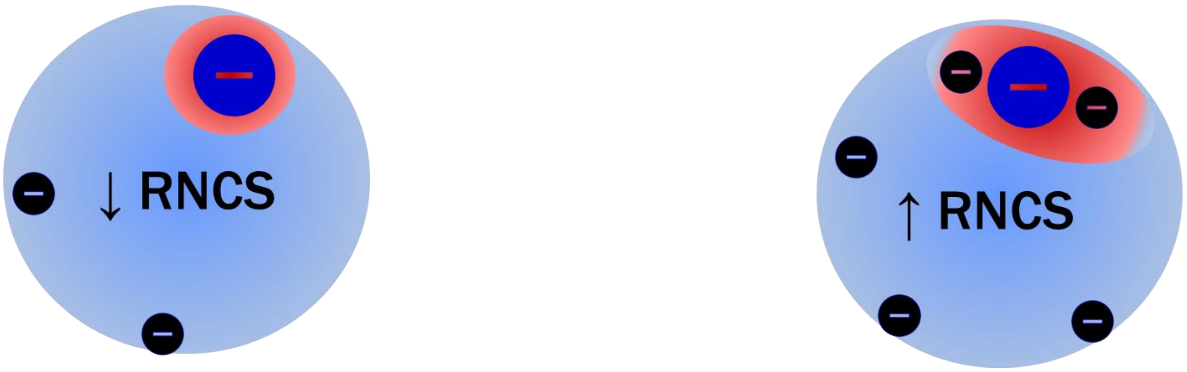

Figure S6. General illustration of molecules with high and low values of RNCG and RNCS.

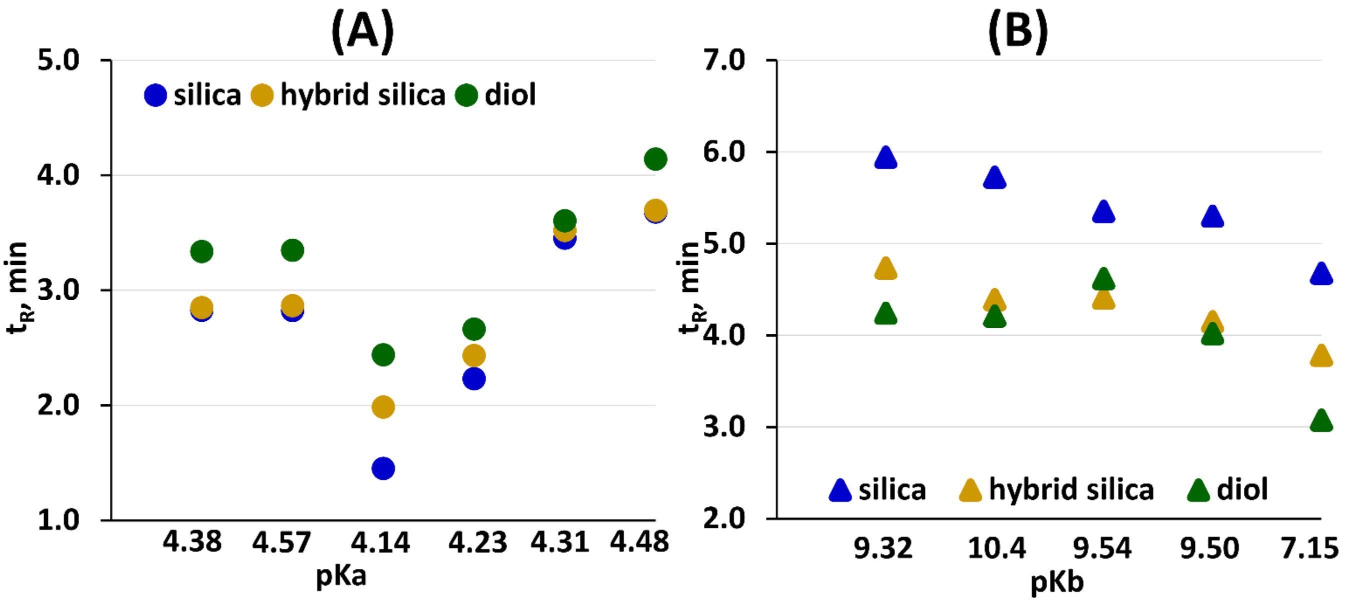

Figure S7. Retention times of selected acidic and alkaline compounds on silica, BEH, and diol column using MeOH as organic modifier. (A) t_R of analytes with pronounced acidic groups, (B) t_R of analytes with pronounced alkaline groups. 4.38 – 3-hydroxycinnamic acid, 4.57 – 4-hydroxybenzoic acid, 4.14 – flurbiprofen, 4.23 – ketoprofen, 4.31 – pravastatin, 4.48 – shikimic acid, 9.32 – darifenacin, 10.4 – desipramine, 9.54 – pindolol, 9.50 – propranolol, 7.15 – vardenafil.

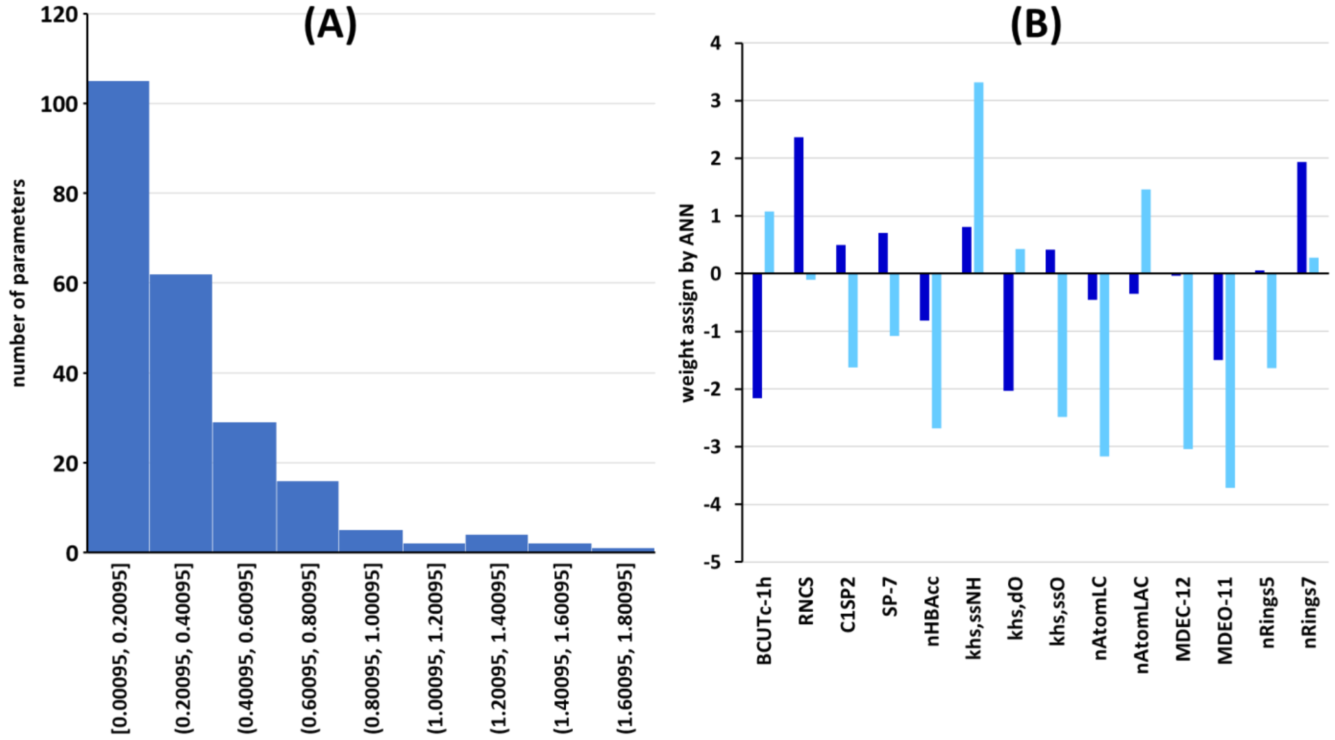

Figure S8. (A) Histogram of standard deviations (SD) between weights of molecular descriptors calculated by ANN using the original and extended set of analytes measured on BEH column using MeOH as organic modifier. (B) Comparison of weights of molecular descriptors with the highest SD.

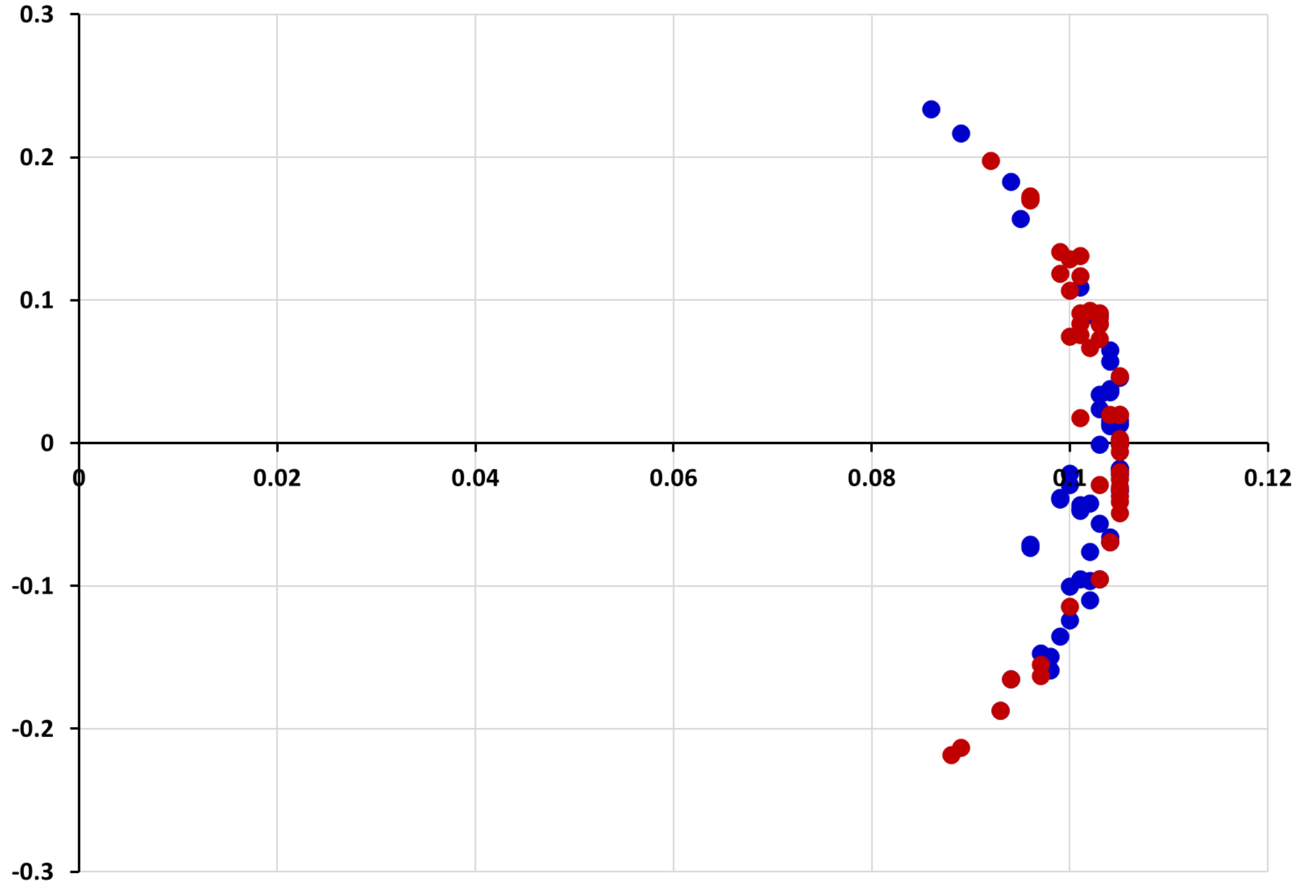

Figure S9. Principal Component Analysis of original set of 52 compounds (blue) and additional compounds eluting on silica and diol column (red).

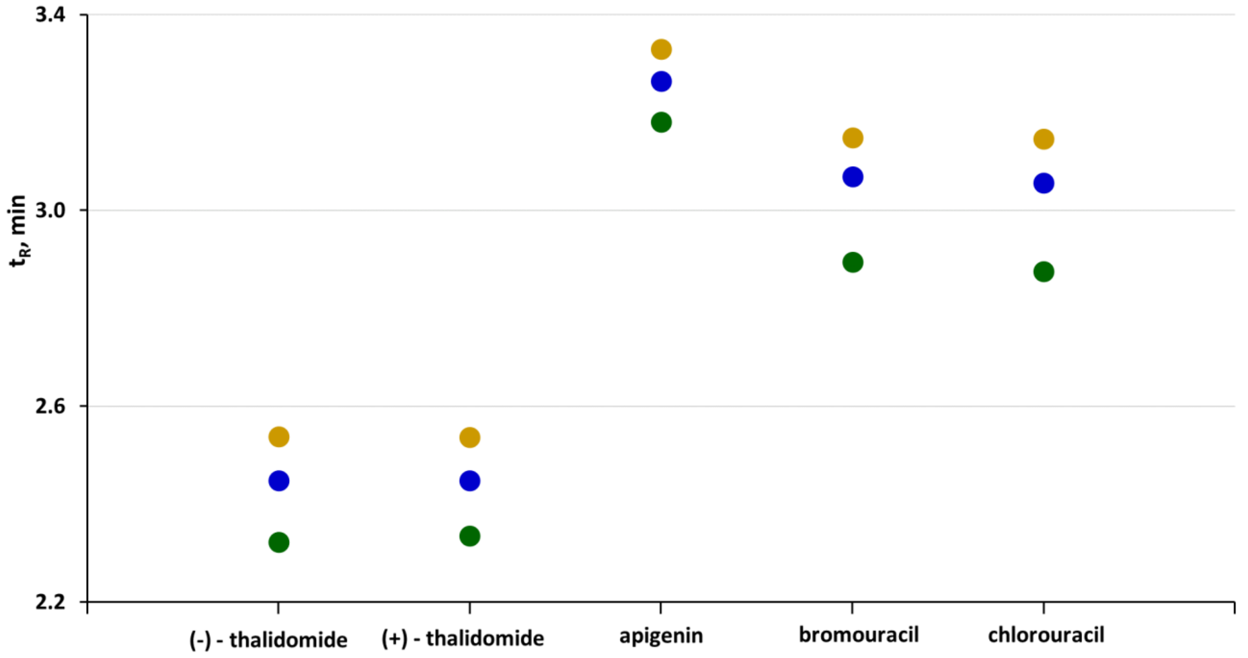

Figure S10. Retention time of the 5 analytes with the highest values of FMF measured on silica column using MeOH (blue), MeOH+2%H_2O (yellow), and MeOh+10 mmol/L NH_3 (green).

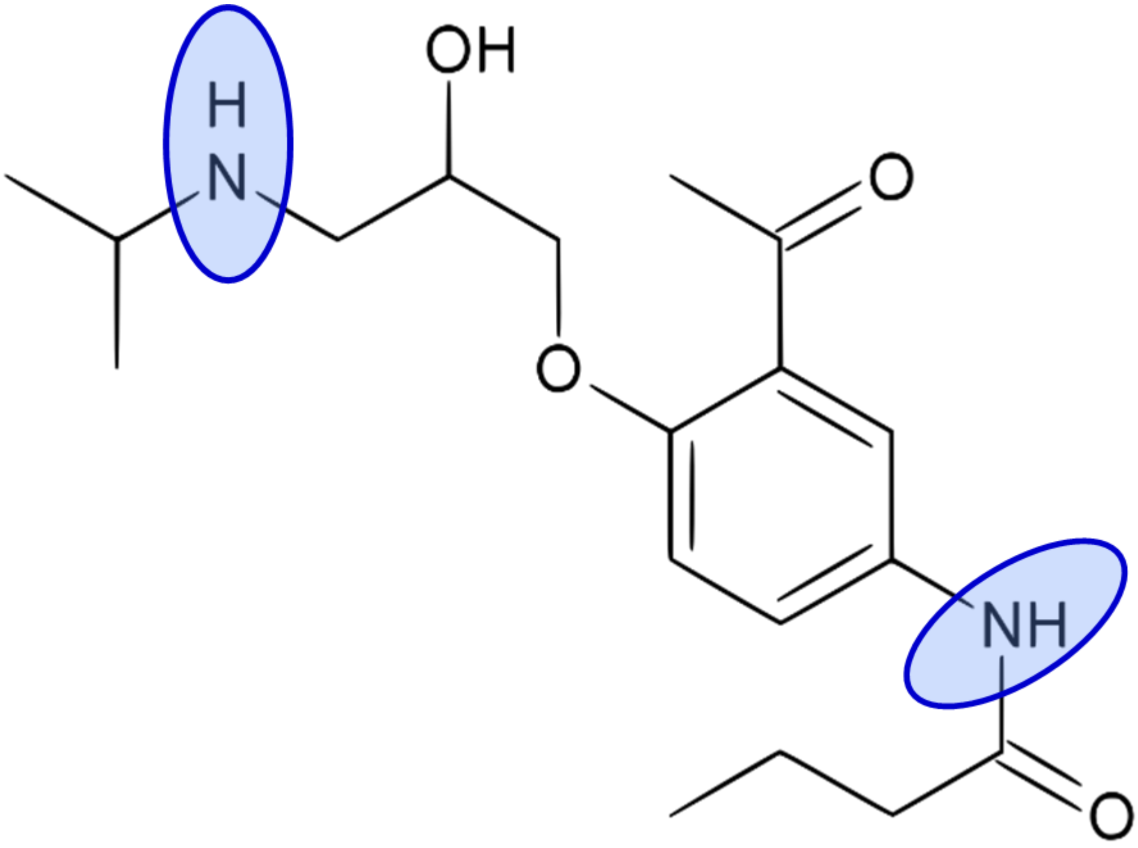

Figure S11. Chemical structure of acebutolol with highlighted -NH- groups.

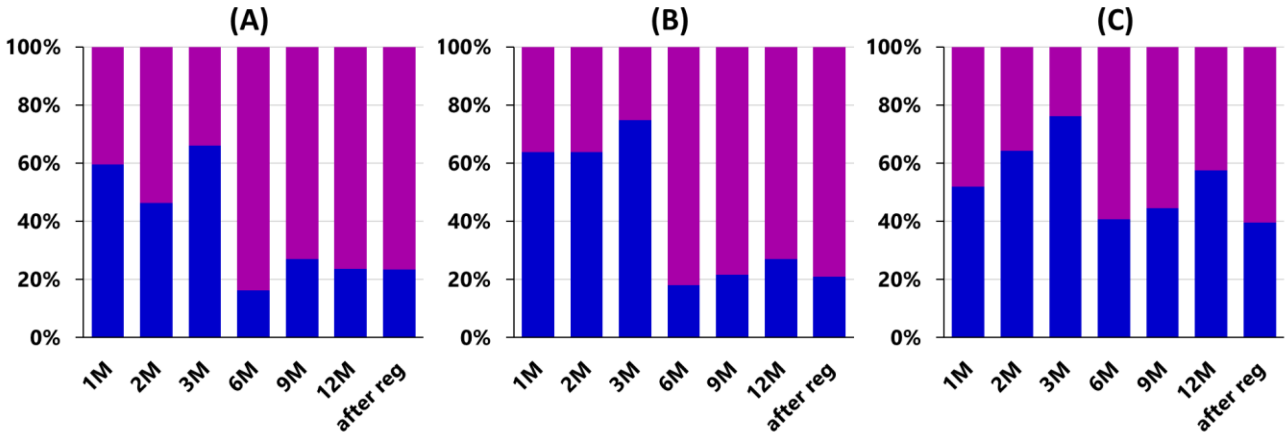

Figure S12. Percentage of analytes with increased (purple) and decreased (blue) retention times at selected data points when compared to the 1st injection on diol column using (A) MeOH, (B) MeOH + 2% H_2O, and (C) MeOH + 10 mmol/L NH_3 as organic modifier.

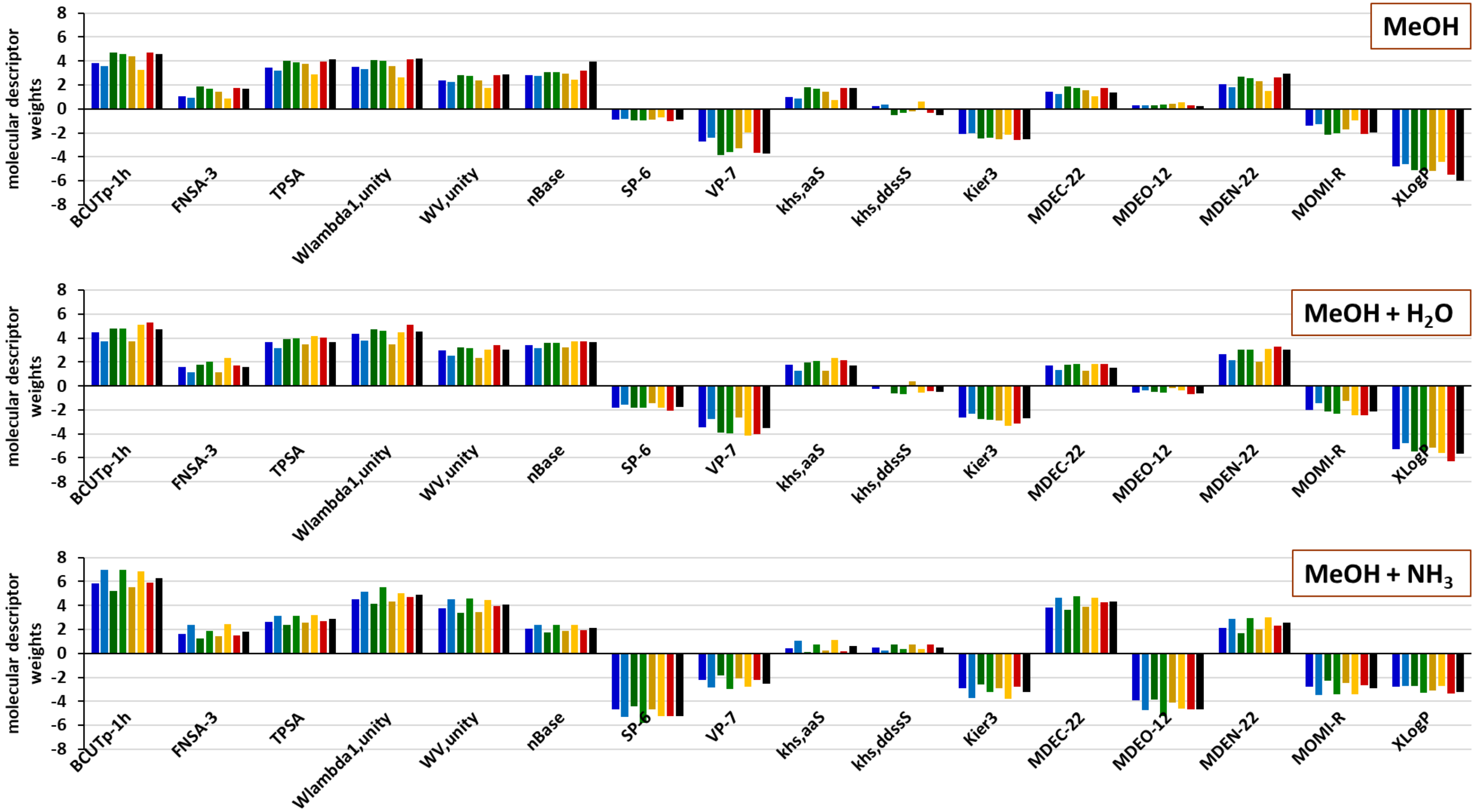

Figure S13. Comparison of weights of the molecular descriptors most affected over time on diol column. Weights determined by ANN based on analysis at 1st injection (dark blue), month 1 (light blue), month 2 (dark green), month 3 (light green), month 6 (dark yellow), month 9 (yellow), and month 12 (red).

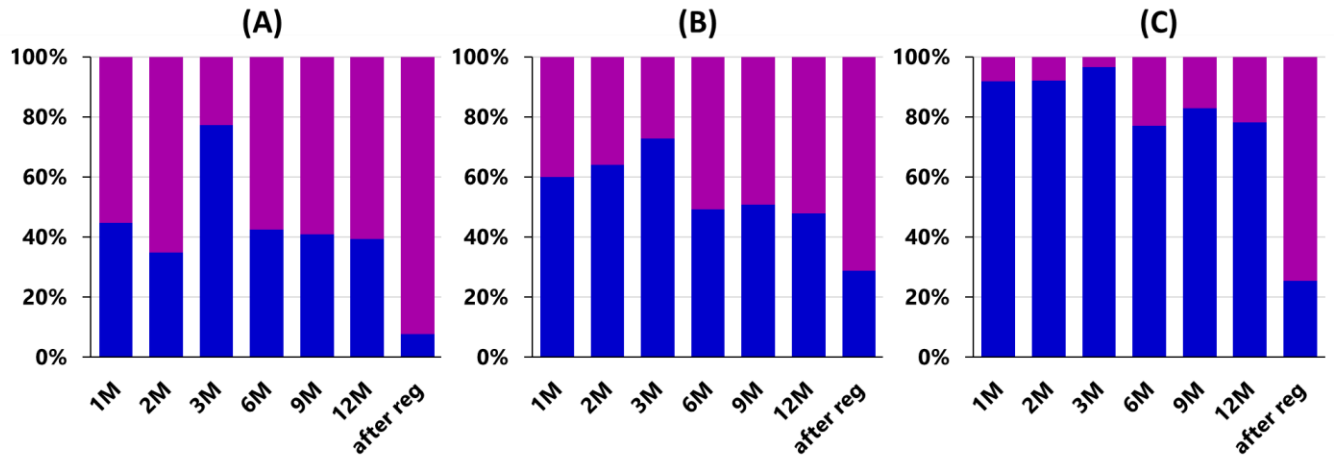

Figure S14. Percentage of analytes with increased (purple) and decreased (blue) retention times at selected data points when compared to the 1st injection on BEH column using (A) MeOH, (B) MeOH + 2% H_2O, and (C) MeOH + 10 mmol/L NH_3 as organic modifier.

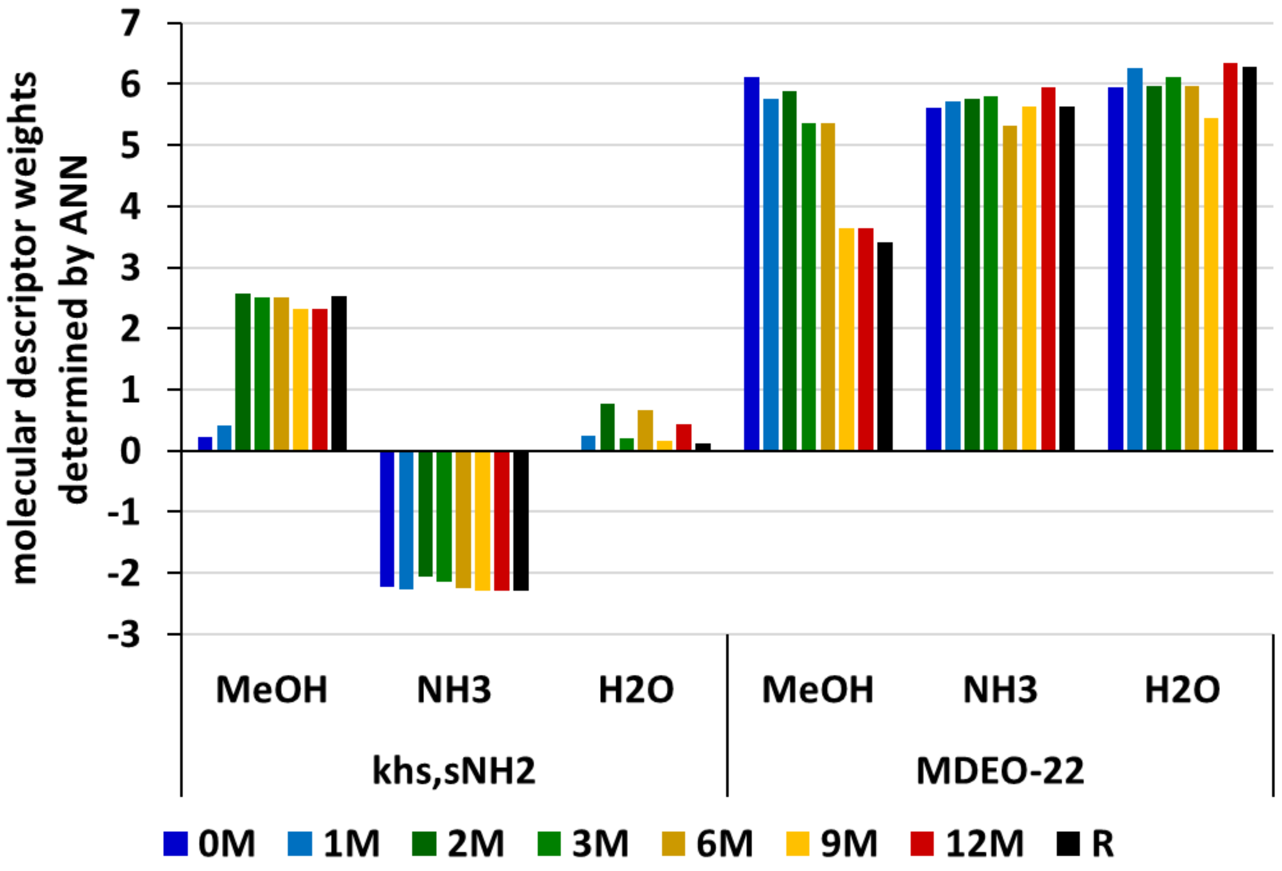

Figure S15. Weights of khs.sNH2 and MDEO-22 molecular descriptors determined by ANN based on the analyses on BEH column at the 1st injection (0M), after 1, 2, 3, 6, 9, and 12 months (M), and after regeneration (R).

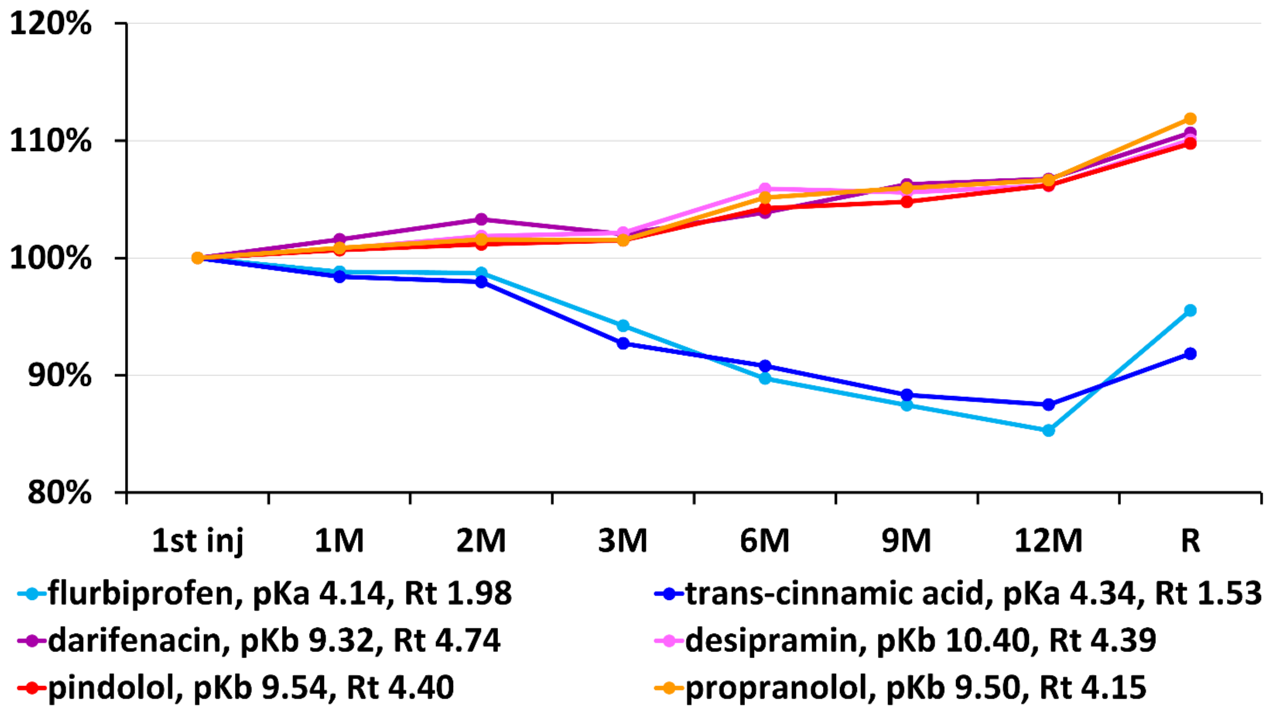

Figure S16. Comparison of retention time shifts over time for alkaline and acidic compounds analyzed on a BEH column using MeOH as organic modifier.

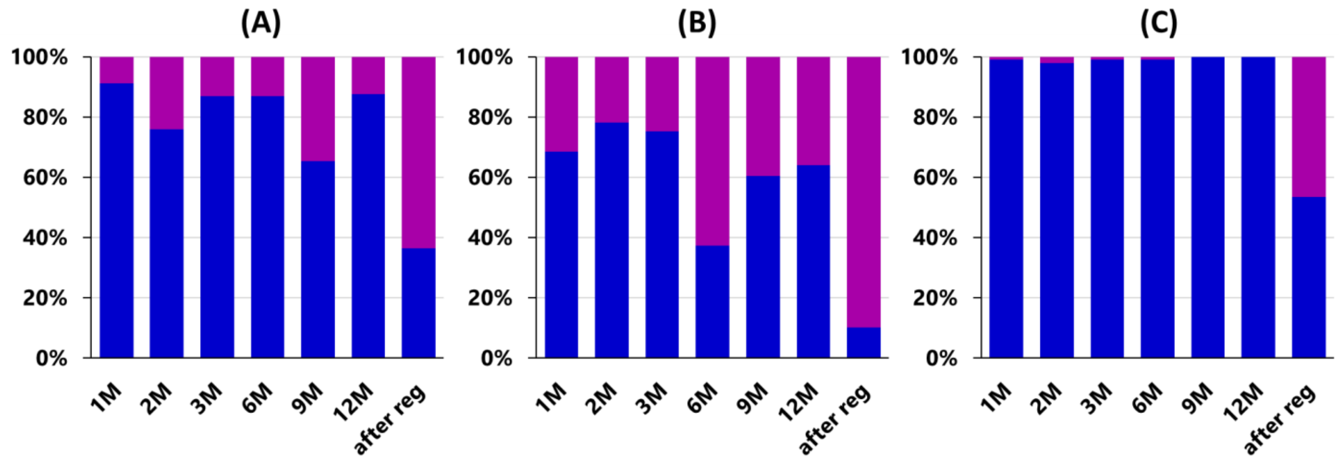

Figure S17. Percentage of analytes with increased (purple) and decreased (blue) retention times at selected data points when compared to the 1st injection on silica column using (A) MeOH, (B) MeOH + 2% H_2O, and (C) MeOH + 10 mmol/L NH_3 as organic modifier.

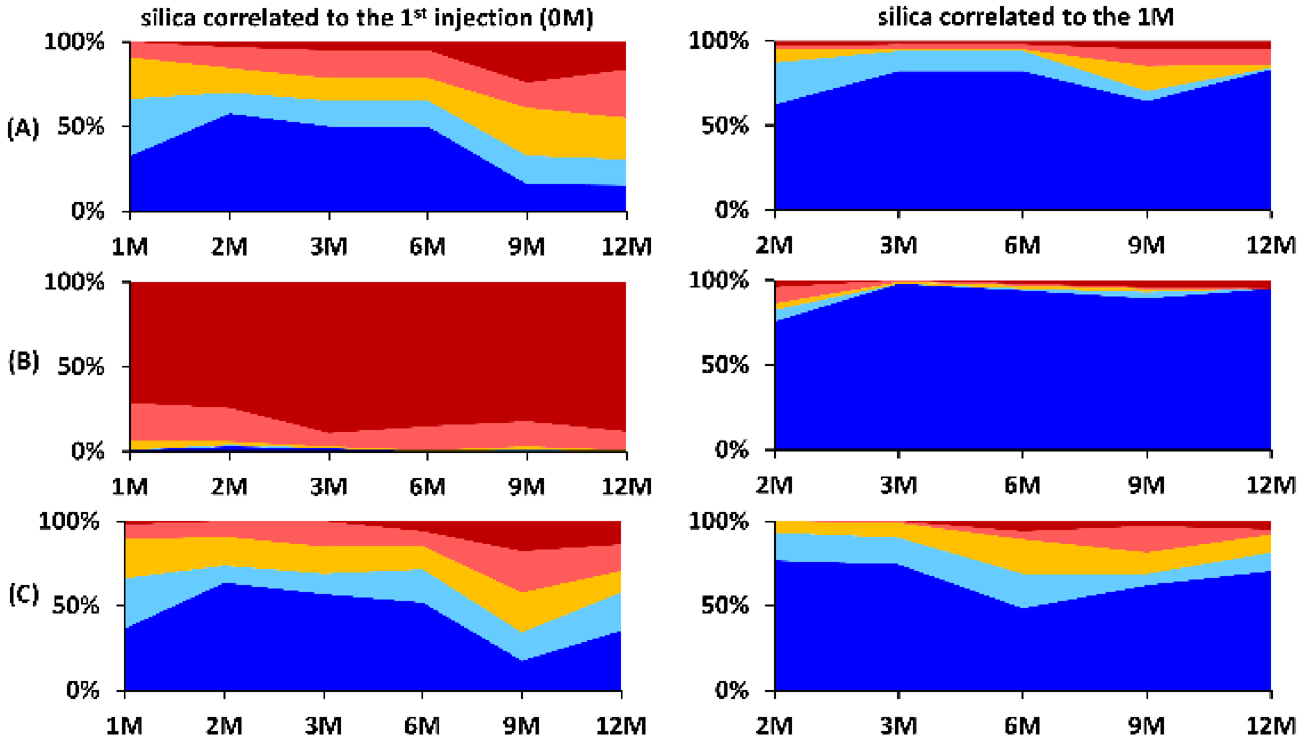

Figure S18: Comparison of retention time shifts over time on silica column using (A) methanol, (B) MeOH+10 mmol/L NH_3, and (C) MeOH+ 2% H_2O as organic modifier.

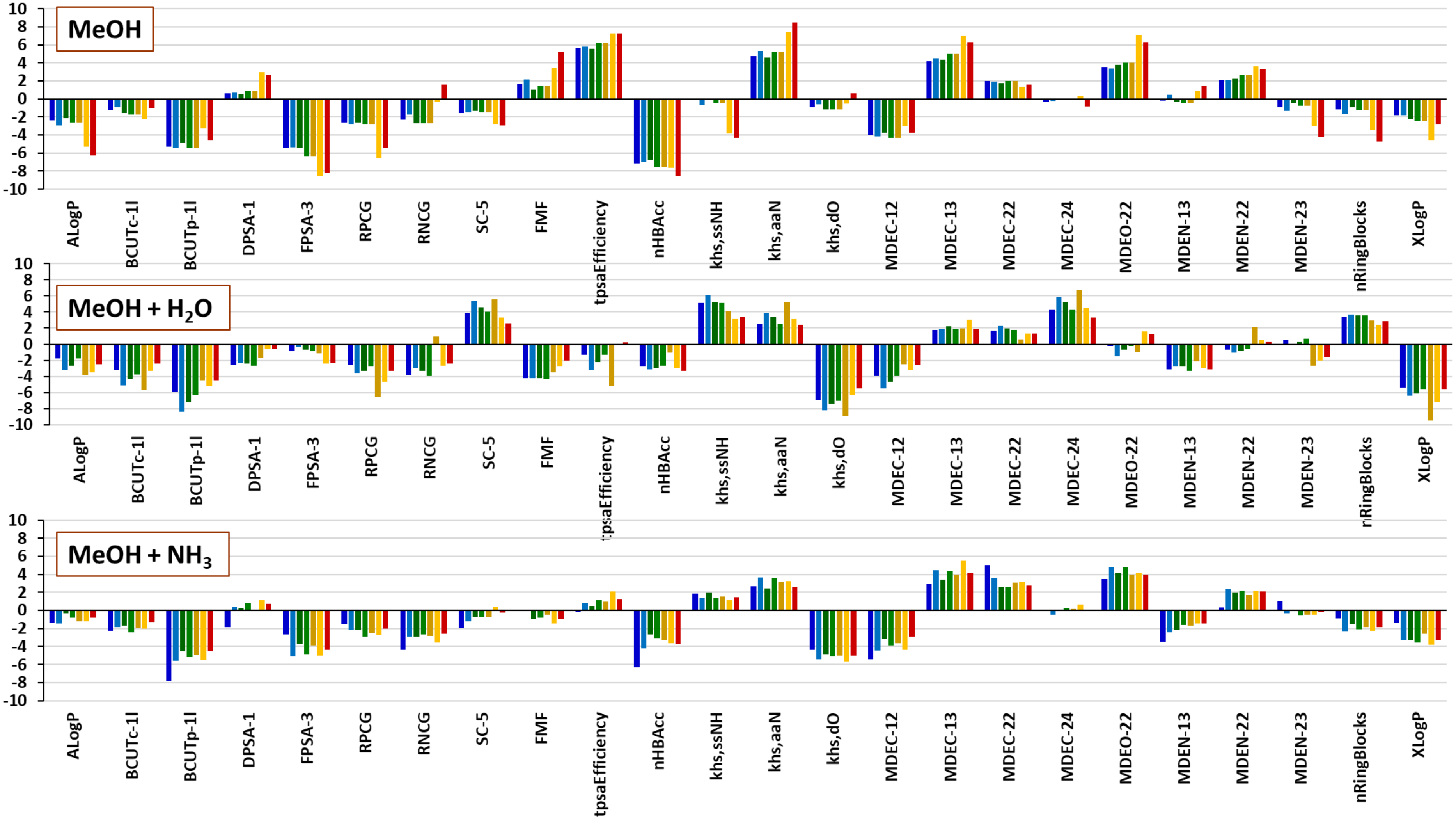

Figure S19. Comparison of weights of the molecular descriptors most affected over time on silica column. Weights determined by ANN based on analysis at 1st injection (dark blue), month 1 (light blue), month 2 (dark green), month 3 (light green), month 6 (dark yellow), month 9 (yellow), and month 12 (red).

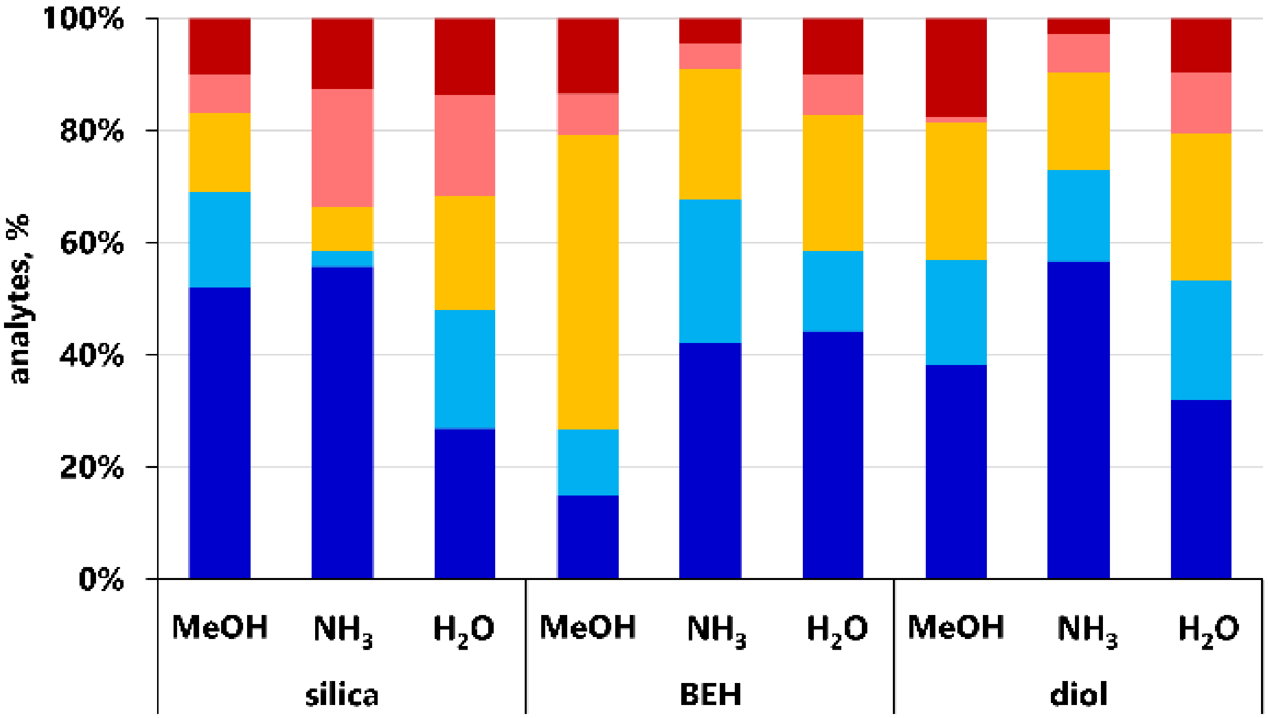

Figure S20: Comparison of % errors calculated between t_R after regeneration and t_R at the first injection on silica, BEH, and diol columns. %-error less than 0.5% (dark blue), 0.5 – 1.0% (light blue), 1.0 -2.0% (yellow), 2.0 -5.0% (pink), and over 5% (red).

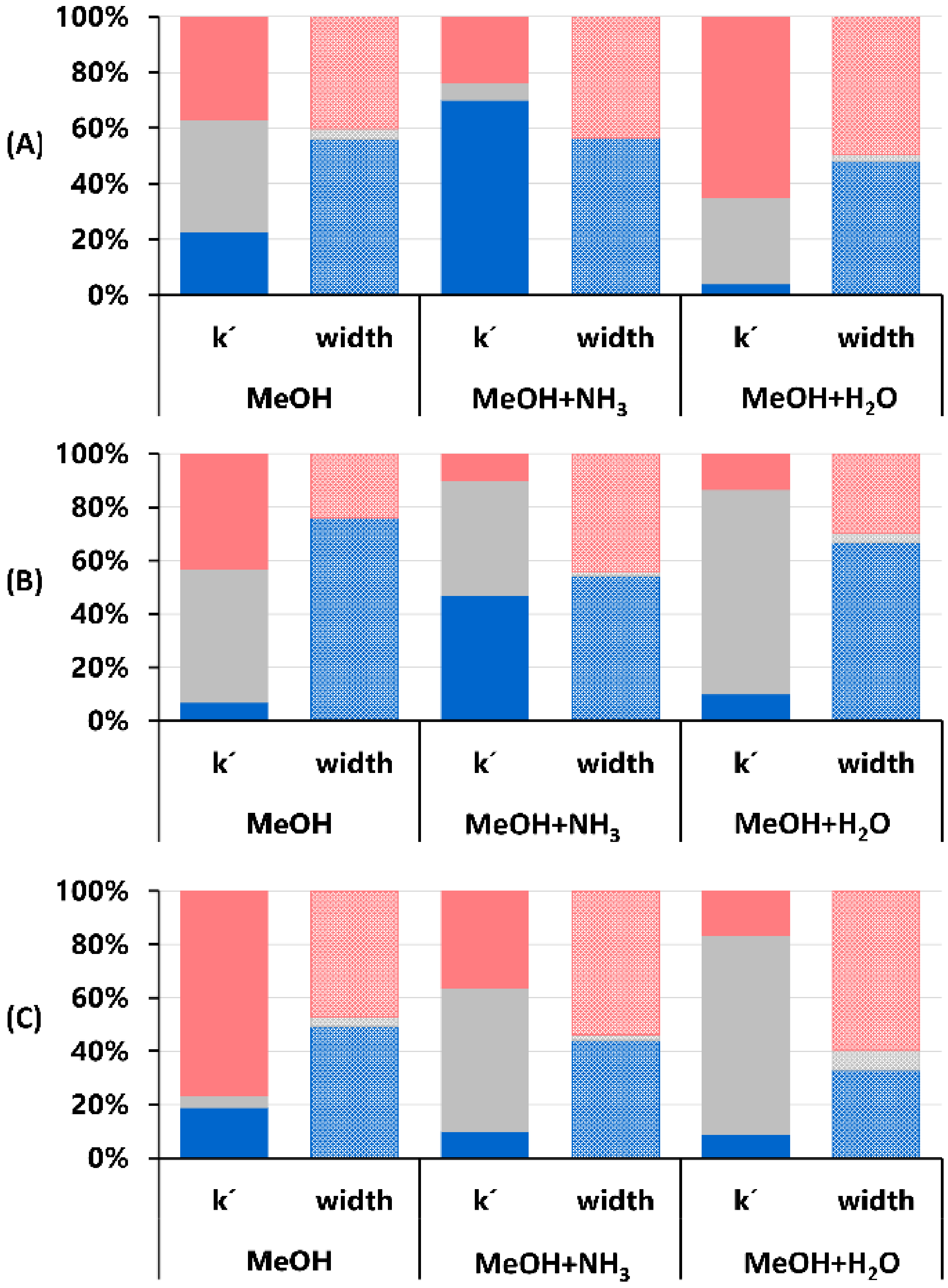

Figure S21: Comparison of changes in retention time and peak width after regeneration procedure. k´ closer to the value of the first injection after regeneration (blue) vs. after 12 months (red). Gray color represents k´ within +/- 2%. (A) – silica column, (B) – BEH column, (C) – diol column.

References

(1) West, C.; Lemasson, E.; Bertin, S.; Hennig, P.; Lesellier, E. *Journal of Chromatography A* **2016**, *1440*, 212-228, DOI: https://doi.org/10.1016/j.chroma.2016.02.052
(2) Gros, Q.; Molineau, J.; Noireau, A.; Duval, J.; Bamba, T.; Lesellier, E.; West, C. *J Chromatogr A* **2021**, *1639*, 461923, DOI: 10.1016/j.chroma.2021.461923
